# The E3 ligase *Ta*E3V-B1 ubiquitinates proteins encoded by the vernalization gene *TaVRN1* and regulates developmental processes in wheat

**DOI:** 10.1093/plphys/kiae606

**Published:** 2024-11-18

**Authors:** Tian Li, Ragupathi Nagarajan, Shujuan Liu, Juan C Luzuriaga, Wenxuan Zhai, Shuanghe Cao, Haiyan Jia, Brett F Carver, Liuling Yan

**Affiliations:** Department of Plant and Soil Sciences, Oklahoma State University, Stillwater, OK 74078, USA; Institute of Crop Sciences, Chinese Academy of Agricultural Sciences, Beijing 100081, China; Department of Plant and Soil Sciences, Oklahoma State University, Stillwater, OK 74078, USA; Institute of Crop Sciences, Chinese Academy of Agricultural Sciences, Beijing 100081, China; Department of Plant and Soil Sciences, Oklahoma State University, Stillwater, OK 74078, USA; Department of Plant and Soil Sciences, Oklahoma State University, Stillwater, OK 74078, USA; Department of Plant and Soil Sciences, Oklahoma State University, Stillwater, OK 74078, USA; Institute of Crop Sciences, Chinese Academy of Agricultural Sciences, Beijing 100081, China; Department of Plant and Soil Sciences, Oklahoma State University, Stillwater, OK 74078, USA; Department of Plant and Soil Sciences, Oklahoma State University, Stillwater, OK 74078, USA; Department of Plant and Soil Sciences, Oklahoma State University, Stillwater, OK 74078, USA

## Abstract

In wheat (*Triticum aestivum*), early maturity is desired to avoid the hot and dry summer season, especially in view of climate change. Here, we report that *Ta*E3V1, a C3H2C3 RING-type E3 ligase that interacts with *Ta*VRN1, is associated with early development. Aside from its RING domain, *Ta*E3V1 does not harbor any domains that are conserved in other RING-type or other E3 ligase proteins. *Ta*E3V-B1b, encoded by the functional *TaE3V1* allele, interacts with and ubiquitinates *Ta*VRN1. In contrast, *Ta*E3V-B1a, encoded by a natural nonfunctional *TaE3V1* allele, neither interacts with *Ta*VRN1 nor has E3 ligase activity. *Ta*E3V-B1b activity decreases with plant age under warmer temperatures, but not under the low temperatures required for vernalization. We employed a gene editing method to simultaneously inactivate the 3 homoeologous *TaE3V1* genes to validate their functions. Overall, our results suggest that the naturally mutated and edited *TaE3V1* alleles can accelerate wheat development and aid adaptation to warming climates.

## Teaser

A RING-type E3 ligase physically interacts with and ubiquitinates a vernalization regulator, thereby altering developmental processes in wheat.

## Introduction

Wheat (*Triticum aestivum*, 2*n* = 6*x* = 42, AABBDD) growth and development comprise 3 critical stages: stem elongation (joining), heading date (flowering time), and physiological maturity (seed production) (McMaster 2005; Chen et al. 2010). Early maturity of wheat enables it to avoid the hot and dry summer season, which is particularly desirable as the global climate shifts toward warmer temperatures (Kerr 2007). Wheat plants, grown over approximately one-third of available arable land in various environmental conditions, have evolved a complex network incorporating internal and external cues to ensure timely flowering and maturity (Yan 2009; Amasino 2010; Srikanth and Schmid 2011). In Arabidopsis (*Arabidopsis thaliana*), nearly 400 genes are known to regulate flowering time, and these genes are involved in several genetically distinct pathways that each perceive signals from vernalization, photoperiod, plant age, phytohormones, and sugar synthesis and utilization (Bernier and Périlleux 2005; Bouché et al. 2016; Xu et al. 2019). Previous studies in wheat have identified a dozen genes that modulate flowering in response to different genetic and environmental cues.

Four wheat vernalization genes involved in the vernalization pathway have been cloned using positional cloning approaches: *VRN1* (the ortholog to Arabidopsis *APETALA 1* [*AP1*]) (Yan et al. 2003), *VRN2* (consisting of the 2 genes *Zinc finger and CCT domain 1* [*ZCCT1*] and *ZCCT2*, with no ortholog in Arabidopsis) (Yan et al. 2004b), *VRN3* (the ortholog to Arabidopsis *FLOWERING LOCUS T* [*FT*]) (Yan et al. 2006), and *VRN4* (the homolog of wheat *VRN1*) (Kippes et al. 2015). These 4 *VRN* genes were cloned based on the qualitative differences in the vernalization requirement between winter wheat cultivars, which require a period of low temperature to accelerate flowering, and spring wheat cultivars, which lack this requirement (Chouard 1960; Yan 2009). Exposure to low temperatures upregulates the transcript levels of *VRN1*, *VRN3*, and *VRN4* and promotes flowering (Danyluk et al. 2003; Murai et al. 2003; Trevaskis et al. 2003; Yan et al. 2006; Kippes et al. 2015), whereas transcript levels of *VRN2*, encoding a dominant repressor of flowering, are downregulated by low temperatures, thus accelerating flowering (Yan et al. 2004a, 2004b). Based on the duration of low temperatures needed to fully satisfy the vernalization requirement, winter wheat cultivars are classified into 3 types: weak, semiwinter, and strong (Crofts 1989). The positional cloning of a major quantitative trait locus responsible for the difference in duration of the low-temperature requirement between semiwinter and strong winter wheat cultivars revealed that 2 recessive alleles (*Tavrn-A1a* in cultivar ‘Jagger’ and *Tavrn-A1b* in cultivar ‘2174’) control the trait at the protein level (Li et al. 2013). The VRN1 protein promotes flowering by directly binding to the CArG-box present in the promoter of the *FT* or *VRN3* genes in wheat (Tanaka et al. 2018).

In the photoperiodic pathway, the cloning of the photoperiod gene *PHOTOPERIOD-H1* from barley (*Hordeum vulgare* L.) (Turner et al. 2005) provided information about how the wheat ortholog *Ppd1* responds to photoperiod (Beales et al. 2007; Wang et al. 2009b). Wheat cultivars can be divided into sensitive and insensitive types based on their responses to photoperiod. Using a positional cloning approach, *TaOGT1*, which encodes an *O*-linked *N*-acetylglucosamine (GlcNAc) transferase, was cloned from a segregating population generated by crossing 2 winter wheat cultivars containing the same alleles for known vernalization and photoperiod genes (Fan et al. 2021). *Ta*OGT1 catalyzes the posttranslational addition of a GlcNAc group to the glycine-rich protein 2 (*Ta*GRP2), a negative regulator of flowering via its direct binding to *TaVRN1* pre-mRNA (Xiao et al. 2014). *Ta*VRN1 was reported to have a physical interaction with VEGETATIVE TO REPRODUCTIVE TRANSITION 2 (*Ta*VRT2) as a repressor of wheat development, which was also regulated during vernalization (Kane et al. 2005; Xie et al. 2019), establishing a flowering time pathway. Signals for flowering can be transmitted from other pathways to *TaVRN1*, which acts as a central regulator of heading date in different wheat species (Yan et al. 2006; Li et al. 2013). For example, low nitrogen conditions promote flowering by upregulating *TaVRN1*, suggesting that flowering time is regulated by signals from the nutrient assimilation pathway (Lei et al. 2018). In the plant physiological maturity pathway, *TaVRN1* is upregulated under ambient conditions rather than low temperatures due to insertions or deletions in the promoter region. These mutations result either in the loss of a repressor recognition site (Yan et al. 2004a, 2004b; Pidal et al. 2009) or the gain of a binding site for the microRNA *Ta*miR1123 (Yu et al. 2014). Repressor binding sites are also located in *TaVRN1* intron 1 (Fu et al. 2005) and its 5′ untranslated region (Chu et al. 2011). In addition, the *TaOGT1b* allele from the cultivar ‘Billings’ has a 168-bp insertion within its intron 1, where a MADS-box protein binding site is located (Fan et al. 2021).

Flowering time in wheat is regulated not only at the transcript level but also at the protein level. For example, multiple chromatin modifications and methylation sites are present across the regulatory regions of *TaVRN1*, and flowering time is regulated by Constans-like B5 (*Ta*Col-B5), a protein that is phosphorylated by the protein kinase *Ta*K4 (Zhang et al. 2022b). Another posttranslational modification affecting flowering time involves BENZOIC ACID HYPERSENSITIVE 1 (*Ta*BAH1, TraesCS5B01G373000), a RING-type E3 ligase that adds multiple ubiquitin (UB) molecules to a target protein, promoting its degradation. *TaBAH1* transcript levels are upregulated during floret primordia development; *Ta*BAH1 interacts with and polyubiquitinates *Ta*SAHH1 (Kim et al. 2021). Histone H2B monoubiquitination enzyme 2 (*Ta*HUB2), another Really Interesting New Gene (RING)-type E3 ligase, ubiquitinates histone H2B in vernalized wheat plants, suggesting that *Ta*HUB2 is also involved in vernalization and may be required for flowering or heading in wheat (Kim et al. 2021). Protein ubiquitination requires the sequential contribution of 3 enzymes: a ubiquitin-activating enzyme (E1), a ubiquitin conjugation protein (E2), and a ubiquitin–protein ligase (E3) (Hellmann and Estelle 2002; Sullivan and Deng 2003; Pickart 2004; Stone et al. 2005).

In this study, we identified an E3 ubiquitin ligase, *Ta*E3V1, that interacts with and mediates the ubiquitination of the vernalization regulator *Ta*VRN1. The genetic analysis of a natural nonfunctional allele in *TaE3V-B1* and gene-edited plants carrying mutant alleles for all 3 *TaE3V1* homoeologs elucidated the contribution of protein degradation via ubiquitination to the vernalization response in wheat and provides germplasm for breeding climate-resilient crops.

## Results

### Interactions of *Ta*VRN-A1 with a RING-type E3 ligase

We previously used *Ta*VRN1 as a bait to screen a yeast two-hybrid (Y2H) library constructed from cDNA prepared from total RNA extracted from 2 winter wheat cultivars of *T. aestivum*, ‘Jagger’ and ‘2074’ (Cao and Yan 2013). From this screen, we identified *Ta*HOX1 (Homeobox 1), *Ta*VRT2, and *Ta*SOC1 (SUPPRESSOR OF OVEREXPRESSION OF CO 1) as interactors of *Ta*VRN1, the central factor in determining the duration of vernalization requirement and heading date (Li et al. 2013; Xie et al. 2019). Two other sets of clones defined additional interacting proteins, but the lack of a published wheat genome at the time prevented their characterization. The recent release of genome sequences for the cultivar ‘Chinese Spring’ (CS) (by the International Wheat Genome Sequencing Consortium; International Wheat Genome Sequencing Consortium (IWGSC) 2018) facilitated the functional exploration of these 2 interacting proteins in the present study.

**Figure 1.**
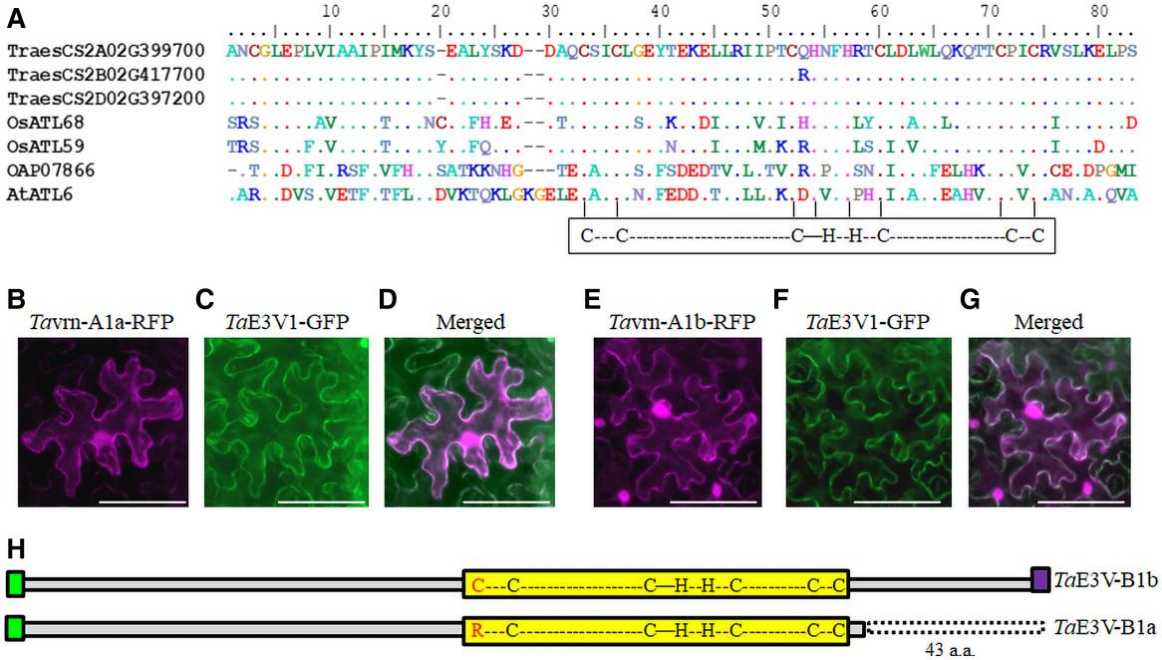
Analyses of *Ta*E3V1 and *Ta*VRN1 proteins. **A)** Multiple sequence alignment of *Ta*E3V1 and related proteins in different plant species. Alignment of the proteins encoded by TraesCS2A02G39970 and its 2 homoeologs, TraesCS2B02G417700 and TraesCS2D02G397200, with *Os*ATL68 (GenBank #XP_015634004), *Os*ATL59 (GenBank #Q9SN27) from rice, and ATL28 (UniProt #Q6NKR1) and ATL6 (GenBank #AAD33584) from Arabidopsis. The structure of the conserved C3H2C3 domain (X2-C-X2-C-X15-C-H-X3-H- X2-C-X10-C, where X is any residue) is shown. **B** to **D)** The colocalization of *Ta*E3V1 and *Ta*vrn-A1a on the membranes. **E** to **G)** The colocalization of *Ta*E3V1 and *Ta*vrn-A1b on the membrane. Constructs encoding *Ta*E3V1-GFP and *Ta*vrn-A1-RFP were coexpressed in *N. benthamiana* leaves via *Agrobacterium*-mediated infiltration. *Ta*E3V1 is encoded by the allele of TraesCS2B02G417700. Scale bars, 100 *μ*m. **H)** Diagram showing the differences between *Ta*E3V-B1a and *Ta*E3V-B1b. *Ta*E3V-B1b in cultivar ‘2174' is functional, while *Ta*E3V-B1a in ‘Jagger' is nonfunctional. The first Cys (C) highlighted in red in the C3H2C3 domain ‘2174' is changed to Arg (R) in the ‘Jagger' cultivar. The loss of 43 amino acids in *Ta*E3V-B1a due to a 2-bp deletion in the ‘Jagger’ genomic sequence is shown.

Of the first set of 5 clones (Supplementary Fig. S1), 4 matched the sequence of TraesCS2A02G399700 in CS, mapping to the long arm of chromosome 2AL. The fifth clone corresponded to TraesCS2B02G417700, one homoeolog of TraesCS2A02G399700, located on the long arm of chromosome 2BL (Supplementary Fig. S2). Another homoeolog is TraesCS2D02G397200 on the long arm of chromosome 2DL. These 3 homoeologous genes consist of 3 exons and 2 introns covering more than 5 kb from the translation start codon to the stop codon and encode proteins of 198 or 199 amino acids that are annotated as putative RING finger proteins (Supplementary Fig. S3A). A RING domain-containing protein may have a Cys/His-rich (C/H) zinc-chelating domain that mediates both protein–protein and protein–DNA interactions (Freemont et al. 1991; Stone et al. 2005; Metzger et al. 2014). This domain is characterized by the C3H2C3 sequence (Fig. 1A; Supplementary Fig. S3B).

The 3 wheat homoeologous RING finger proteins shared the highest identity with ARABIDOPSIS TOXICOS EN LEVADURA 68 (*Os*ATL68) and *Os*ATL59 from rice (*Oryza sativa*) and ATL28 and ATL6 from Arabidopsis (*A. thaliana*) over the short region covering the C2H2 domain. However, the rice or Arabidopsis proteins were not functionally characterized. Many RING finger proteins are E3 ubiquitin ligases (Bocock et al. 2011), and the ATL proteins were suggested to have E3 ligase activity (Stone et al. 2005). Furthermore, the second set of *Ta*VRN1-interacting proteins matched ubiquitin, raising the possibility that these RING finger proteins are E3 ubiquitin ligases. Therefore, we named these wheat E3 proteins interacting with *Ta*VRN1 as *Ta*E3V1. Most RING domain-containing proteins in plants are localized in the nucleus and cytoplasm (Cao et al. 2008; Sun et al. 2019). However, we identified no clear classic nuclear localization signal in these 3 RING finger proteins (Supplementary Fig. S3C).

We determined the localization of the *Ta*E3V1 protein encoded by TraesCS2B02G417700 as a fusion to the yellow fluorescent protein (YFP), which revealed a location at the plasma membrane (Supplementary Fig. S4). The *Ta*VRN1-YFP fusion was previously detected predominantly in the nucleus and the plasma membrane (Li et al. 2013), which was confirmed in this study (Fig. 1, B and E). To verify the colocalization of *Ta*VRN1 and *Ta*E3V1, *Ta*E3V1-GFP and *Ta*VRN1-RFP fusion proteins were coexpressed in plant cells, and their localizations overlapped at the plasma membrane (Fig. 1, B to G).

### Differential interactions of naturally mutated *TaE3V-B1* proteins with *Ta*vrn-A1 proteins

We identified 2 alleles for *TaE3V-B1* (TraesCS2B02G417700 on chromosome 2B) in the 2 winter wheat cultivars, ‘Jagger’ and ‘2174’. The ‘Jagger’ allele is referred to as *TaE3V-B1a*, and the ‘2174’ allele is referred to as *TaE3V-B1b*. The letters a and b were added to the names of the 2 alleles to be consistent with the allele names described in previous studies involving multiple genes (Li et al. 2013). ‘2174’ has the same *TaE3V-B1b* allele as CS, but ‘Jagger’ has a single nucleotide polymorphism (SNP) in an exon causing a Cys-to-Arg change (C92R), together with a 2-bp deletion that causes a shift in the reading frame (Fig. 1H; Supplementary Fig. S5). *Ta*E3V-B1b in 2174 has a Cys residue at position 92, corresponding to the first C in the C3-H2-C3 domain, which is replaced with Arg (R) in *Ta*E3V-B1a in ‘Jagger’. This C92R substitution may thus influence its E3 ubiquitin ligase activity or protein–protein interactions. The 2-bp deletion in the *TaE3V-B1a* allele is predicted to result in a loss of 43 residues (from residue 157 to residue 199) at the C-terminus of *Ta*E3V-B1a (Fig. 1H).

**Figure 2.**
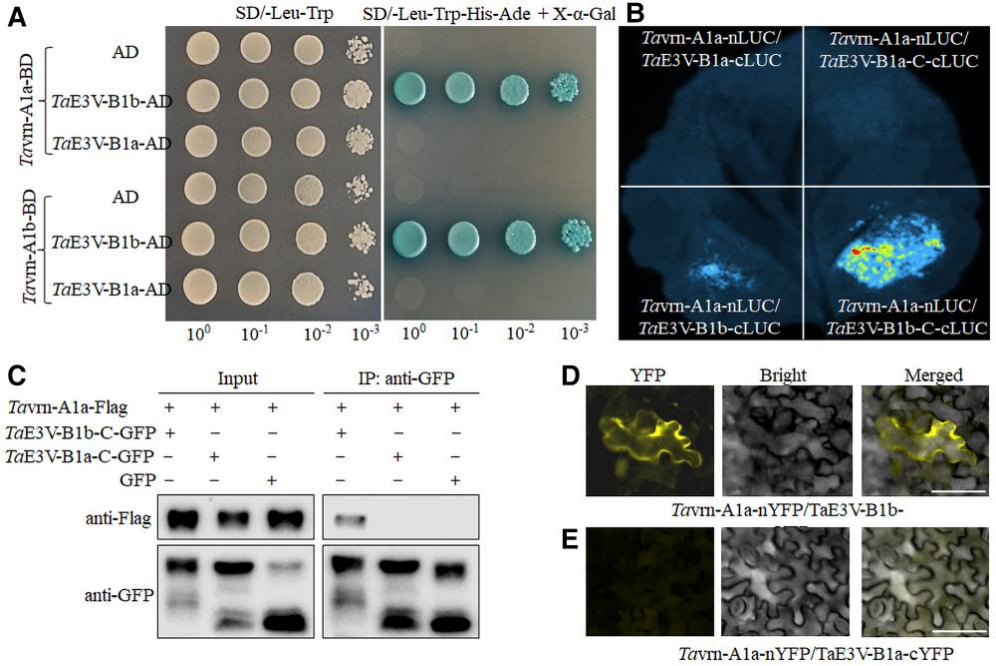
Protein analyses of *Ta*E3V-B1 and *Ta*VRN1. **A)** Interaction of *Ta*E3V-B1 and *Ta*VRN1 in a Y2H assay. *Ta*vrn-A1a was from ‘Jagger', *Ta*vrn-A1b was from ‘2174', *Ta*E3V-B1a was from ‘Jagger', and *Ta*E3V-B1b was from ‘2174'. Cotransformed cells were grown on a SD medium lacking Leu and Trp (SD/-Leu/-Trp) and on an SD medium lacking Leu, Trp, His, and Ade (SD/-Leu/-Trp/-His/-Ade). Four 10-fold dilutions from each yeast colony were spotted on the same plates. AD, activation domain; BD, binding domain. **B)** Interaction of *Ta*E3V-B1 and *Ta*vrn-A1a in a LCI assay. The LCI assay was performed in *N. benthamiana* leaves. *Ta*E3V-B1a from ‘Jagger' and *Ta*E3V-B1b from ‘2174': the full-length protein with cLUC; *Ta*E3V-B1-C the truncated protein including the C-terminus (from residue 78 to the C-terminus). nLUC, N-terminal portion of LUC; cLUC, C-terminal portion of LUC. The 4 combinations of *Ta*E3V-B1 and LUC were tested on the same leaf. **C)** Co-IP assays of potential interactions between *T*aE3V-B1 and *Ta*vrn-A1a. Total proteins were extracted and incubated with anti-GFP-conjugated magnetic agarose beads (MBL) for IP. Proteins before (input) and after IP were detected with anti-GFP and anti-Flag antibodies. *Ta*E3V-B1-C, the truncated protein including the C-terminus (from residue 78 to the C-terminus). **D**, **E)** Interaction of *Ta*E3V-B1 and *Ta*VRN1 in a BiFC assay. The BiFC assays were performed in epidermal cells of *N. benthamiana* leaves. *Ta*E3V-B1a from ‘Jagger'; *Ta*E3V-B1b from ‘2174’. nYFP, N-terminal portion of YFP; cYFP, C-terminal portion of YFP. Scale bars: 50 *μ*m.

We tested *Ta*E3V-B1a and *Ta*E3V-B1b (from residue 78 to the end of C-terminus) against 2 *Ta*vrn-A1 proteins, *Ta*vrn-A1a from ‘Jagger’ and *Ta*vrn-A1b from ‘2174’. These *Ta*vrn-A1 proteins differ only by 2 substitutions, L117F and A180V (Li et al. 2013). We observed the interaction of *Ta*vrn-A1a and *Ta*vrn-A1b with *Ta*E3V-B1b from ‘2174’ but not with *Ta*E3V-B1a from ‘Jagger’ in the Y2H system (Fig. 2A).

We further confirmed the interaction between *Ta*E3V-B1 and *Ta*vrn-A1 through the firefly luciferase complementation imaging (LCI) assay in *Nicotiana benthamiana* leaves. The results showed that the full-length *Ta*E3V-B1b with LUC (*Ta*E3V1b-cLUC) and the truncated *Ta*E3V-B1b from residue 78 to the end of the C-terminus (*Ta*E3V-B1b-C) with LUC (*Ta*E3V1b-C-cLUC) interacted with *Ta*vrn-A1a in plant cells, but the full-length *Ta*E3V-B1a and its truncated form did not interact with *Ta*vrn-A1a in plant cells (Fig. 2B). Immunoblot analysis using an anti-LUC antibody confirmed all these proteins were expressed in plant cells (Supplementary Fig. S6A), ruling out the possibility that the interaction was false due to variations in protein expression levels. Notably, the truncated *Ta*E3V-B1b had much more robust interactions with *Ta*vrn-A1 than the full-length *Ta*E3V-B1b, probably because the N-terminal 77 a.a. containing the transmembrane domains hindered their interactions. The differential interactions of *Ta*E3V-B1a and *Ta*E3V-B1b proteins with the *Ta*vrn-A1a protein were validated in their interactions with the *Ta*vrn-A1b protein (Supplementary Fig. S6, B and C). In addition, a coimmunoprecipitation (Co-IP) assay showed that the *Ta*vrn-A1a-Flag protein was coprecipitated with the *Ta*E3V-B1b-C-GFP protein but not with the *Ta*E3V-B1a-C-GFP protein and the GFP control (Fig. 2C), further confirming the in vivo interaction of *Ta*vrn-A1a with *Ta*E3V-B1b.

The direct interaction between the full-length *Ta*E3V-B1b and *Ta*vrn-A1 was validated using the bimolecular fluorescence complementation (BiFC) assay. YFP signals were observed in the plasma membrane in the cells coexpressing *Ta*E3V-B1b-nYFP and *Ta*vrn-A1a-cYFP (Fig. 2D), but no YFP signals were observed in the cells coexpressing *Ta*E3V-B1a-nYFP and *Ta*vrn-A1a-cYFP (Fig. 2E). The differential interactions of *Ta*E3V-B1a and *Ta*E3V-B1b proteins with the *Ta*vrn-A1b protein were also observed (Supplementary Fig. S7). The appearance of the *Ta*E3V-B1b and *Ta*vrn-A1 interaction signals in the plasma membrane was consistent with their colocalization.

### *Ta*UBV1 was the ubiquitin of *Ta*E3V1 ubiquitinating the *Ta*VRN1 proteins

The second set of *Ta*VRN1-interacting proteins included 7 clones corresponding to ubiquitin (UB) (Supplementary Fig. S8), a small (8.5 kDa) regulatory protein present in almost all tissues of eukaryotic organisms and mediating the degradation of their target proteins. These UB-encoding clones comprised 12.5% of the total 56 positive clones obtained (Supplementary Fig. S8), suggesting that they represent true interactions rather than false positives. The clones matched TraesCS6A01G136600, but this gene was annotated as encoding a protein distinct from UB in the HighConf database in IWGSC RefSeq v2.1 on the opposite strand of the sequences (Supplementary Fig. S9). Two clones represented a genomic region on chromosome 7D, in which no gene was annotated, but this region was only 12 kb away from TraesCS7D02G443100, which was annotated as UB and contained 3 copies with 100% identity to the UB encoded by our Y2H clones.

These UBs encoded by the genes on chromosome 7D are 100% identical to their homoeologs on chromosome 6A. The last Y2H clone represented TraesCS5B02G080200 on chromosome 5B, which also encodes UB and has 2 identical homoeologs (Supplementary Fig. S10). We identified over 93 genes encoding UB with high sequence similarity to the identified UB across the wheat genome. Except for a few minor differences, all the UB proteins identified here have the same sequence, including the lysine residues K6, K11, K27, K29, K33, K48, and K63, which are known to be responsible for their attachment to their targets (Supplementary Fig. S10) (Xu et al. 2009). We, therefore, refer to these wheat UB related to wheat *Ta*VRN1 as *Ta*UBV1. The interactions of *Ta*VRN1 with *Ta*E3V1 and *Ta*UBV1 raised the possibility that *Ta*E3V1 might mediate the ubiquitination of *Ta*VRN1 by adding *Ta*UBV1. To test this model, we explored the enzymatic activity of *Ta*E3V1b.

### Enzymatic activity of *Ta*E3V-B1b

**Figure 3.**
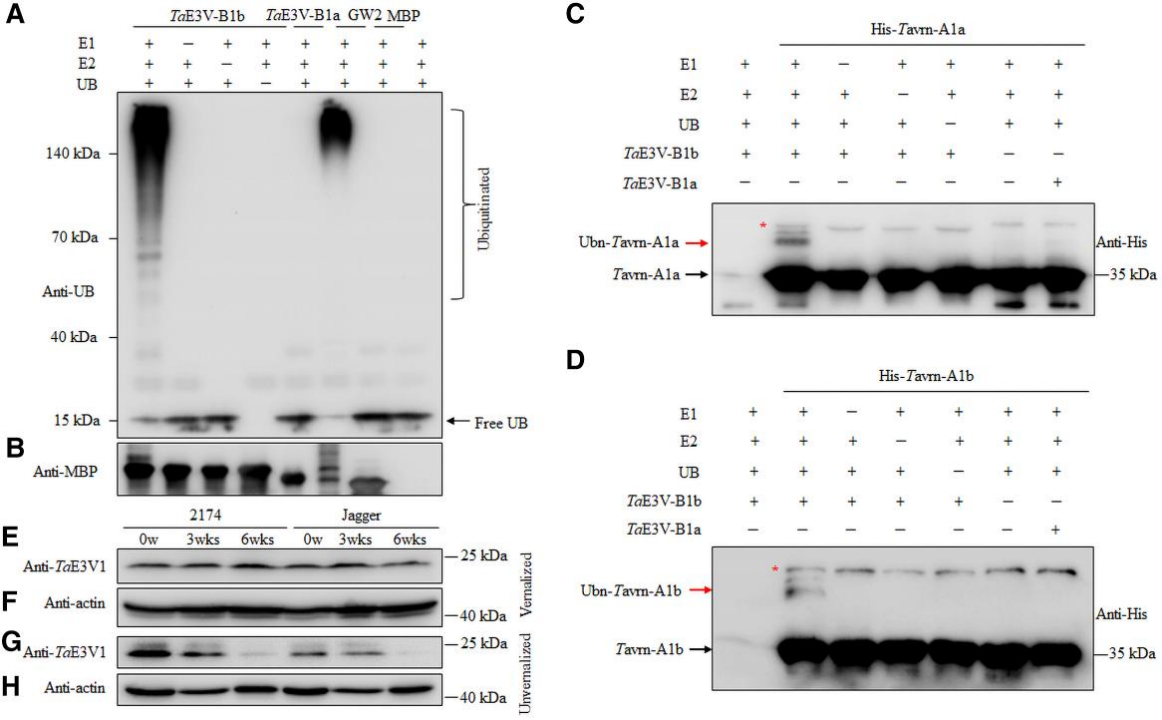
UB ligase activity and protein levels of *Ta*E3V1. **A**, **B)** In vitro ubiquitination assay. Recombinant MBP-*Ta*E3V1 was assayed for UB ligase activity in combinations of E1, E2, and UB. In the presence of all 3 components, *Ta*E3V-B1b encoded by ‘2174' showed UB ligase activity, but *Ta*E3V-B1a encoded by ‘Jagger' did not. The numbers on the left denote protein molecular mass markers. GW2 was used as a positive control, and MBP was used as a negative control. An anti-UB antibody was used to detect ubiquitinated proteins (**A**, upper panel), and an anti-MBP antibody was used to detect MBP (**B**, lower panel). **C**, **D)***Ta*E3V1-mediated ubiquitination of *Ta*VRN1. Recombinant MBP-*Ta*E3V-B1 and His-*Ta*VRN1 were used in the in vitro ubiquitination assay in combinations of E1, E2, and UB. *Ta*E3V-B1b from ‘2174' could ubiquitinate *Ta*vrn-A1a from ‘Jagger' and *Ta*vrn-A1b from ‘2174', but *Ta*E3V-B1a from ‘Jagger’ could not. An anti-His antibody was used to detect both nonubiquitinated and ubiquitinated Tavrn-A1 proteins. The red arrow showed the ubiquitinated Tavrn-A1 (Ubn-Tavrn-A1), and the red asterisk indicates the nonspecific band. **E**, **F)***Ta*E3V1 abundance in nonvernalized plants (0w) and plants vernalized for 3 wk (3wks) or 6 wk (6wks), as determined with an anti-*Ta*E3V1 antibody. Actin was used as a loading control. **G**, **H)***Ta*E3V1 abundance in nonvernalized plants, as determined with an anti-*Ta*E3V1 antibody. Actin was used as a loading control.

We conducted an in vitro ubiquitination assay to determine whether *Ta*E3V-B1 had UB ligase activity. We cloned full-length sequences of *TaE3V-B1b* and *TaE3V-B1a* into a maltose-binding protein (MBP) fusion vector for expression in *Escherichia coli*. We then tested the recombinant purified MBP-tagged proteins for self-ubiquitination activity by incubating them with recombinant E1, E2, and UB, followed by immunoblot analysis with an anti-UB antibody (Fig. 3A) and with an anti-MBP antibody as a control (Fig. 3B). In the presence of all 3 components, we detected ubiquitinated proteins when *Ta*E3V-B1b was also present but did not find any ubiquitinated proteins when *Ta*E3V-B1a was present. By contrast, in the absence of single components (E1, E2, or UB), we observed no polyubiquitinated conjugates (Fig. 3A). In the same assay in the presence of E1, E2, and UB, we employed the E3 ligase GRAIN WIDTH and WEIGHT2 (GW2) as a positive control (Zhang et al. 2022a, 2022b), while MBP was used as a negative control. These results indicate that *Ta*E3V-B1b functions as an E3 ligase but that *Ta*E3V-B1a is nonfunctional.

Since *Ta*E3V-B1b is a functional E3 ligase and directly interacts with *Ta*VRN1 protein, we hypothesize that *Ta*E3V-B1b can mediate the ubiquitination of *Ta*VRN-A1 protein. We performed an in vitro ubiquitination assay using recombinant MBP-*Ta*E3V-B1 and His-*Ta*VRN1 proteins to test this hypothesis. The assay showed that *Ta*E3V-B1b from ‘2174’ could ubiquitinate His-*Ta*vrn-A1a from ‘Jagger’ in the presence of E1, E2, and UB, while *Ta*E3V-B1a from ‘Jagger’ did not exhibit this activity (Fig. 3C). The absence of any components, including E1, E2, UB, or *Ta*E3V-B1b, abolished the ubiquitination of the *Ta*vrn-A1a protein in the assay (Fig. 3C). The capability of *Ta*E3V-B1b ubiquitinating the His-*Ta*vrn-A1b from ‘2174’ was also confirmed (Fig. 3D). We also confirmed the ubiquitination of the *Ta*vrn-A1a protein by *Ta*E3V-B1b by transient expression assays in *N. benthamiana* leaves. After immunoprecipitation (IP) using anti-Flag antibody, ubiquitinated *Ta*vrn-A1a-Flag protein can be detected in the coexpression with *Ta*E3V-B1b-C, not *Ta*E3V-B1a-C (Supplementary Fig. S11). These results provided direct evidence that *Ta*E3V-B1b mediated the ubiquitination of the *Ta*VRN1 protein.

Using an anti-*Ta*E3V1 antibody, we performed an immunoblot assay to determine whether *Ta*E3V-B1b and *Ta*E3V-B1a abundance is altered by vernalization or as the plant ages. Notably, we observed no changes in the levels of *Ta*E3V-B1b or *Ta*E3V-B1a following 3 or 6 wk of vernalization treatment (Fig. 3, E and F). However, *Ta*E3V-B1b and *Ta*E3V-B1a levels decreased as plants aged without vernalization (Fig. 3, G and H).

### Association of allelic variants for *TaE3V-B1* with heading date

**Figure 4.**
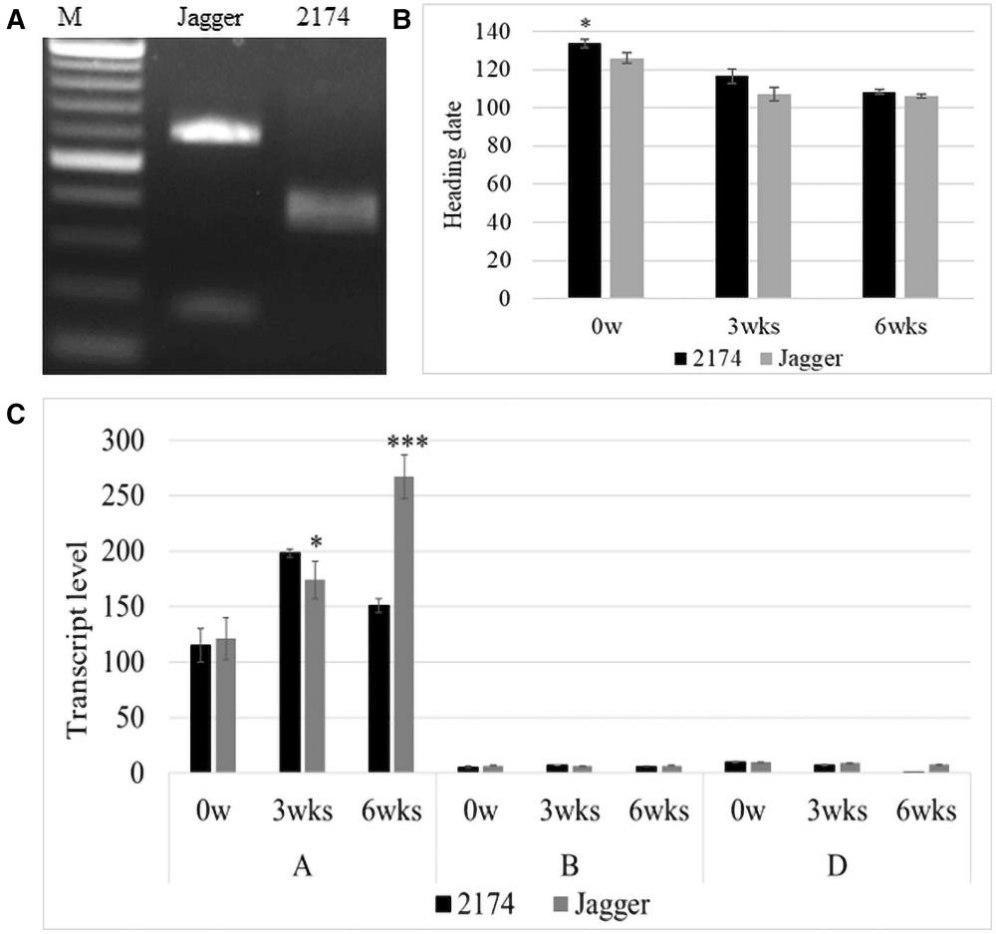
Allelic variation in *TaE3V-B1* and its phenotypic effect. **A)** PCR marker for genotyping of *TaE3V-B1* alleles. A marker for the SNP involving the C92R substitution differing between *Ta*E3V-B1a and *Ta*E3V-B1b was developed. The digested PCR products were resolved on a 2% (w/v) agarose gel. M, DNA ladder. **B)** Effects of the different *TaE3V1* alleles on heading dates of wheat plants. ‘Jagger' plants carrying the *TaE3V-B1a* allele and ‘2174’ carrying the *TaE3V-B1b* allele were vernalized for 3 weeks (3wks) or 6 weeks (6wks); plants without vernalization (0w) were used as controls. **C)** The transcript levels of homoeologous *TaE3V1* genes by RT-qPCR (*n* = 9). Transcript levels were normalized to *Actin* as a reference transcript. **B**, **C)** Values are means ± se. A *t* test was conducted to determine the significance levels: *P < 0.05 and ***P < 0.001.

*Ta*vrn-A1a in ‘Jagger’ and *Ta*vrn-A1b in ‘2174’ caused different vernalization requirement durations (Li et al. 2013). We had phenotyped a recombinant inbred line (RIL) population generated by crossing ‘Jagger’ with ‘2174’ for numerous traits in previous studies, including the required duration of vernalization, stem elongation, heading date, and physiological maturity. We developed a genetic marker to genotype the SNP behind the C92R substitution in *Ta*E3V-B1a (Fig. 4A). When plants from this population were grown in the greenhouse, the mutation in the *TaE3V-B1a* allele was associated with the heading date. Without vernalization, plants carrying the *TaE3V-B1a* allele exhibited a heading date that was 8 d earlier than those carrying the *TaE3V-B1b* allele, a significant difference (*P* < 0.03). By contrast, the heading date of plants having the *TaE3V-B1a* allele was not significantly different from that of plants having the *TaE3V-B1b* allele when the population was vernalized for 3 wk (*P* = 0.07) or 6 wk (*P* = 0.18) (Fig. 4B). These results indicated the development process under the condition without vernalization was associated with a genetic factor at the *TaE3V-B1* locus, suggesting that plants carrying the nonfunctional *TaE3V-B1a* allele or an unknown gene at this locus promoted the development process. If *TaE3V-B1b* was the causal allele, it encoded a repressor of the development process in wheat, possibly by degrading *Ta*VRN1.

### Effects of editing *TaE3V1* genes

The 3 homoeologous *TaE3V1* genes showed significant differences in their transcript levels. *TaE3V-A1* transcript levels were more than 10-fold higher than those of *TaE3V-B1* and *TaE3V-D1*, regardless of vernalization and vernalization durations (Fig. 4C). These unexpected results may explain why we obtained more Y2H clones from the homoeolog on chromosome 2A than those on chromosomes 2B and 2D.

To test the functional consequences of losing all 3 homoeologs, we designed a single guide RNA (sgRNA) targeting exon one, 84 bp downstream from the translation start codon. The sgRNA had the same sequence as *TaE3V-A1* and *TaE3V-D1* but one mismatch with the sequence of *TaE3V-B1*. However, we identified editing events in all 3 homoeologous genes following clustered regularly interspaced short palindromic repeat (CRISPR)/CRISPR-associated nuclease 9 (Cas9)-mediated editing in the ‘Bobwhite’ cultivar using the *TaE3V1* sgRNA editing construct.

**Figure 5.**
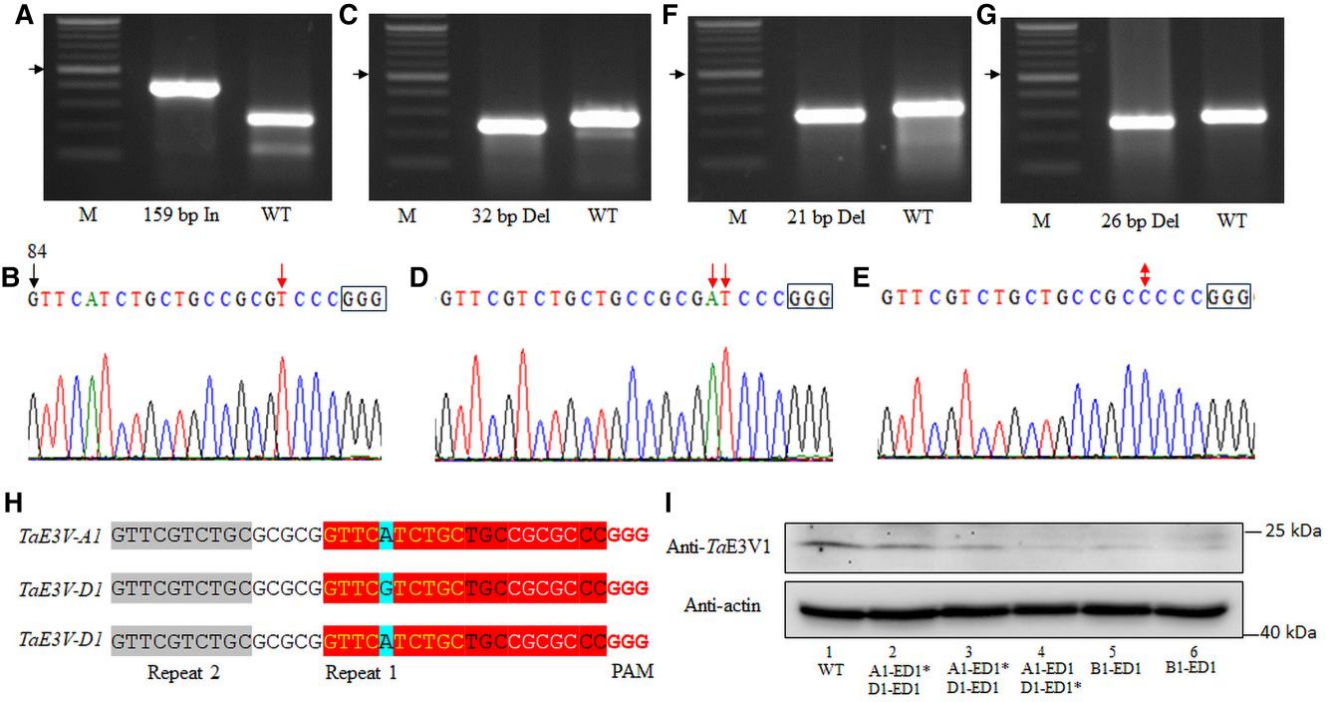
Editing of *TaE3V1* genes in transgenic plants. **A)** Genotyping PCR of *TaE3V-A1-ED1*. The expected size of the PCR product using primers E3S1-AF and E3S1-AR is 245 bp in wild type (WT). *TaE3V-A1-ED1* has a 159-bp insertion, resulting in a 404-bp PCR product. **B)** The edited sequence in *TaE3V-A1-ED2*. The chromatogram shows the sequences of the 20-bp sgRNA and downstream PAM sequence (GGG, black square); 84 is the position of the first nucleotide of the *TaE3V1* sgRNA counting from the translation start codon. The red arrow indicates the 1-bp insertion in *TaE3V-A1-ED2*. **C)** Genotyping PCR of *TaE3V-B1-ED1*. The expected size of the PCR product using primers E3S1-BF and E3S1-BR is 274 bp in WT. *TaE3V-B1-ED1* has a 32-bp deletion, resulting in a 242-bp PCR product. **D)** The edited sequence in *TaE3V-B1-ED2*. The red arrows indicate the 2-bp insertion. **E)** The edited sequence in *TaE3V-B1-ED3*. The 2-headed arrow indicates the 1-bp nucleotide change from G to C. **F)** Genotyping PCR of *TaE3V-D1-ED2*. The expected size of the PCR is 287 bp in WT. *TaE3V-B1-ED1* has a 21-bp deletion, resulting in a 266-bp PCR product. Genotyping PCR of *TaE3V-D1-ED1*. **G)** The expected size of the PCR product using primers E3S1-DF and E3S1-DR is 287 bp in WT. *TaE3V-B1-ED1* has a 26-bp deletion, resulting in a 261-bp PCR product. **H)** Analysis of sgRNA target sequences in *TaE3V1* homoeologous copies. The sgRNA target sequences of *TaE3V1* are highlighted with a red background. One SNP within the sgRNA in the 3 homoeologous genes is highlighted in cyan. The 10 bp in yellow letters within the sgRNA (repeat 1) are identical to the upstream 10 bp highlighted in a gray background (repeat 2). The 5 bp with no background within the sgRNA is identical to the 10 bp between repeat 1 and repeat 2. **I)** Immunoblot analysis using an anti-TaE3V1 antibody showing *Ta*E3V1 abundance in the samples from the edited plants. 1, WT Bobwhite; 2, A1-2-3 (heterozygous *TaE3V-A1-ED1* and homozygous *TaE3V-D1-ED1*); 3, A1-2-5 (heterozygous *TaE3V-A1-ED1* and homozygous *TaE3V-D1-ED1*); 4, A1-2-7 (homozygous *TaE3V-A1-ED1* and heterozygous *TaE3V-D1-ED1*); 5, B3-4-1 (homozygous *TaE3V-B1-ED1*); 6, B3-4-4 (homozygous *TaE3V-B1-ED1*). The asterisk on the figure indicates “heterozygous.” The proteins were extracted from the leaves of plants 2 weeks after planting in the greenhouse. An antiactin antibody was used as a loading control.

We obtained 2 editing events in *TaE3V-A1*, which were *TaE3V-A1-ED1* and *TaE3V-A1-ED2*. *TaE3V-A1-ED1* had a 159-bp insertion between the 4th and 5th nucleotides upstream of the PAM (Fig. 5A; Supplementary Fig. S12). *TaE3V-A1-ED2* had a 1-bp insertion at position 4 upstream of the PAM (Fig. 5B). For *TaE3V-B1*, we obtained 3 editing events. *TaE3V-B1-ED1* had a 32-bp deletion, including 10 bp upstream of the sgRNA target site, 20 bp of the complete sgRNA target site, and 2 bp downstream (Fig. 5C; Supplementary Fig. S13). *TaE3V-B1-ED2* had a 2-bp insertion at position 4 upstream of the PAM (Fig. 5D). In *TaE3V-B1-ED3*, a C was replaced by a G at position 4 upstream of the PAM, resulting in a substitution of the wild-type Ala residue with a Pro residue in the protein encoded by the edited gene (Fig. 5E). We identified 2 editing events in *TaE3V-D1*, and these were named *TaE3V-D1-ED1* and *TaE3V-D1-ED2*. *TaE3V-D1-ED1* had a 21-bp deletion that included 15 bp of the sgRNA target site, the PAM, and 3 bp downstream of the sgRNA target site (Fig. 5F; Supplementary Fig. S14). *TaE3V-D1-ED2* had a 26-bp deletion that included the entire 20 bp sgRNA target site, the PAM, and 3 bp from upstream or downstream (Fig. 5G), where it has the same GCG (Fig. 5H; Supplementary Fig. S14).

We identified many more editing events at the sgRNA target site in *TaE3V1*, compared to the *DES1* (*Death of the entire seedling 1*) (Jia et al. 2021) and *TaCol-B5* (Zhang et al. 2022a, 2022b) genes that we tested in the same gene editing system with their respective sgRNAs. The sgRNA consisted of a direct repeat of 10 bp in the *TaE3V1* sgRNA and an indirect repeat of 5 bp upstream of the sgRNA. These repeats formed a palindromic structure on the same strand of DNA. The 2 repeated sequences have only one nucleotide mismatch (Fig. 5H), resulting in the mistaken recognition of the sgRNA and, thus, multiple editing events. We tested the accumulation of *Ta*E3V1 proteins encoded by all 3 homoeologs by immunoblot analysis using an anti-TaE3V1 antibody and an antiactin antibody as a loading control. In total protein extracts, *Ta*E3V1 protein levels were markedly lower in the edited plants compared to the wild-type plants (Fig. 5I). *TaE3V-A1* transcript levels were more than 10-fold higher than those of *TaE3V-B1* and *TaE3V-D1* (Fig. 4C), but the protein level of *Ta*E3V1 in the plants (lanes 2 to 4), in which *Ta*E3V-A1 was edited alleles, were higher than that in the plants (lanes 5 to 6), in which TaE3V-B1 was edited. It is not uncommon for the protein levels to show inconsistency with the transcriptional levels, possibly due to the different modifications and degradations of different TaE3V1 proteins.

**Figure 6.**
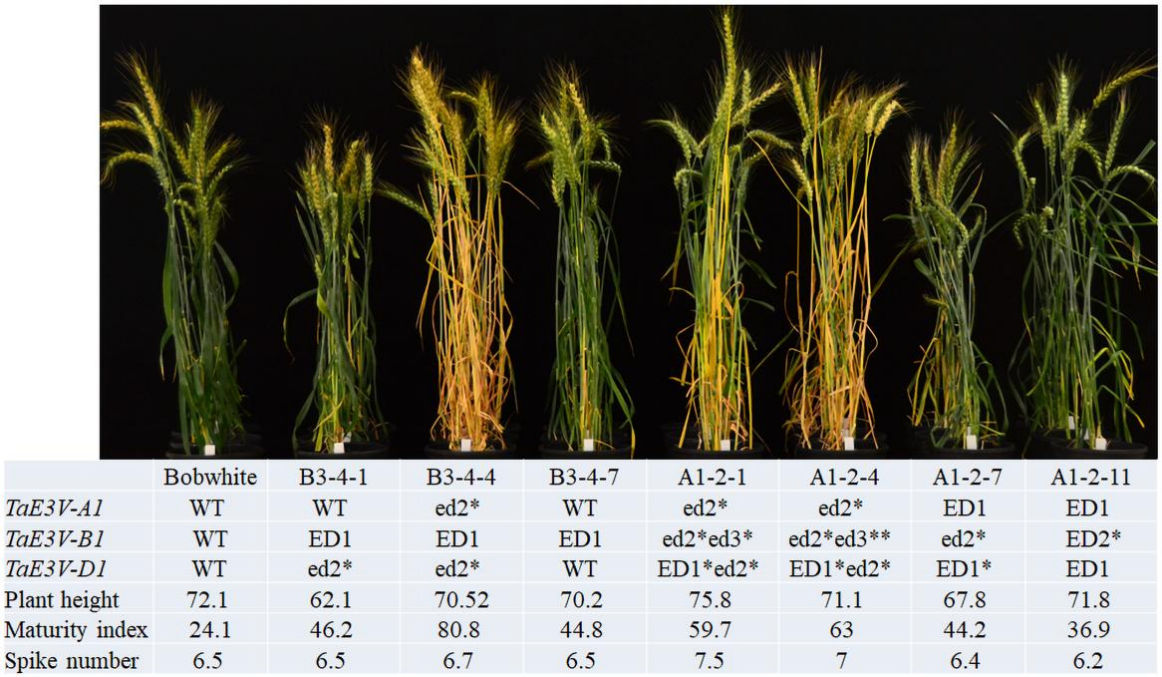
Effects of edited *TaE3V1* alleles in transgenic plants. Photograph showing phenotypic differences among WT Bobwhite and 7 lines harboring edited *TaE3V1* alleles in the T_2_ families. Three T2 families (B3-4-1, B3-4-4, and B3-4-7) were generated from the T1 plants of B3, and 4 T_2_ families (A1-2-1 and A1-2-4 are edited at *TaE3V-D1-ED1*; A1-2-7 and A1-2-11) were generated from the T_1_ plants of A1. WT is the wild type of each *TaE3V-A1*, *TaE3V-B1*, and *TaE3V-D1* in Bobwhite. “ED” indicates the editing events that were identified in the T_1_ plants, and “ed” indicates the editing events that were newly identified in the T_2_ plants. The asterisk indicates “heterozygous.” The phenotypic data were the average of 12 to 24 plants in these T_2_ families.

We followed the above editing events in different plants of the T_1_ and T_2_ generations. We obtained 2 positive T_0_ plants (A1 and B3) but found no editing event. The T_1_ plants generated from A1 carried edited alleles at *TaE3V-A1* and *TaE3V-D1*, with the mutations *TaE3V-A1-ED1* with a 159-bp insertion and *TaE3V-D1-ED1* with a 21-bp deletion. The T_1_ plants generated from B3 had a mutation in *TaE3V-B1*, with *TaE3V-B1-ED1* with a 32-bp deletion. We selected 4 T_1_ plants from the A1-2 line to generate T_2_ lines: A1-2-1 and A1-2-4 (heterozygous for *TaE3V-D1-ED1*), A1-2-7 (heterozygous for *TaE3V-D1-ED1* and homozygous for *TaE3V-A1-ED1*), and A1-2-11 (homozygous for *TaE3V-D1-ED1* and *TaE3V-A1-ED1*) (Fig. 6). Similarly, we selected 3 T_1_ plants from the B3-4 lines (B3-4-1, B3-4-4, and B3-4-7) that were all homozygous for *TaE3V-B1-ED1* to generate T_2_ lines (Fig. 6).

We grew all 7 T_2_ lines in the same greenhouse under photoperiod- and temperature-controlled conditions; we noticed the segregation of several visible phenotypes, including differences in flowering time and maturity, number of spikes per plant, and plant height, among and within some lines. In addition to the known edited genes identified in the T_1_ lines, we detected new editing events in the T_2_ lines, including *TaE3V-A1-ED2*, *TaE3V-B1-ED2*, and *TaE3V-B1-ED3*. To assess the effect of genotypes on the development process, we genotyped these T_2_ lines and concluded that T_2_ plants edited at one gene (for example, A1-2-4 plants carrying *TaE3V-D1-ED1* and B3-4-4 plants having *TaE3V-B1-ED1*) showed earlier maturity than plants from the wild-type ‘Bobwhite’. These 2 edited lines showed no significant differences in plant height (cm) (A1-2-4, 70.5 ± 0.8, *n* = 50; wild type, 72.2 ± 0.6, *n* = 12, *P* = 0.295; B3-4-4, 71.1 ± 0.8, *n* = 50, *P* = 0.467). However, for T_2_ plants with more *TaE3V1* genes edited, some lines showed less of an effect on the time of maturity, but significant effects on plant height, for example, A1-2-7 (67.8 ± 0.9, *n* = 24; wild type, 72.2 ± 0.6, *n* = 12, *P* = 0.002) and B3-4-1 (62.1 ± 0.9, *n* = 29, *P* = 9.3E^−09^).

## Discussion

Semiwinter wheat cultivars like ‘Jagger’ require 3 weeks of vernalization, whereas strong winter wheat cultivars like ‘2174’ require 6 weeks to satisfy their full cold exposure requirements (Li et al. 2013). This difference in vernalization duration was used as the basis for the positional cloning of *Tavrn-A1* (Li et al. 2013). *Ta*vrn-A1 differed by 1 amino acid between the 2 cultivars: Ala-180 in ‘Jagger’ and Val-180 in ‘2714’, with the A180V substitution resulting in altered interaction between *Ta*vrn-A1 and *Ta*HOX1. However, it was unclear how the A180V substitution affected the duration of the vernalization requirement in winter wheat. In this study, we discovered that the E3 ligase activity of *Ta*E3V-B1b, another interactor of *Ta*vrn-A1, decreases with plant age under warmer temperatures but not under low temperatures. We propose that when a semiwinter wheat cultivar such as ‘Jagger’ is exposed to low temperatures for 3 weeks to satisfy its vernalization requirement but if the plants remain at this low temperature after the vernalization, then they will not flower due to the high E3 ligase activity of *Ta*EV3-B1 and the subsequent ubiquitination of *Ta*vrn-A1. When the semiwinter wheat is fully vernalized after 3 weeks of cold treatment and then moved to warmer temperatures, *Ta*E3V1 activity gradually decreases, leading to lower ubiquitination of *Ta*vrn-A1 and, thus, earlier flowering. Our previous study showed that the flowering time of semiwinter and strong winter wheat cultivars did not significantly differ if they were vernalized for 6 weeks. The flowering time of oververnalized winter wheat will not be accelerated but delayed, and fully vernalized winter wheat will respond to ambient temperature as spring wheat (Snape et al. 2001; Li et al. 2013).

In this study, we discovered and demonstrated that wheat *Ta*VRN1 is a substrate for *Ta*E3V1-mediated ubiquitination. In Arabidopsis, several proteins interact with RING-type E3 ligases to be targeted for their degradation (Lim et al. 2010; Park et al. 2018). The transcription of *FLOWERING LOCUS C* (*FLC*) is accelerated by the methylation of histone H3 Lys residues by HISTONE MONOUBIQUITINATION1 (HUB1) and HUB2, the Arabidopsis homolog of the yeast E3 enzyme monoubiquitinated H2B (H2Bub1) in *Saccharomyces cerevisiae* (Cao et al. 2008). HUB2 is highly similar to 3 wheat proteins (encoded by TraesCS3D02G466100, TraesCS3A02G467300, and TraesCS3B02G511800) that share no conserved domain with *Ta*E3V1, which is identified in this study. *Ta*HUB2 is a UB RING-type E3 ligase, *Ta*H2B (histone H2B) being a target substrate protein of *Ta*HUB2. The effect of H2Bub1 in vernalized plants and the levels of H2Bub1 significantly decrease after vernalization (Kim et al. 2021). Wheat does not have an ortholog of *FLC*, and the Arabidopsis ortholog of wheat *TaVRN1* is *AP1*, which does not participate in the vernalization pathway in Arabidopsis. Investigating when and how *Ta*E3V1 ubiquitinates *Ta*VRN1 in wheat plants will be intriguing.

Ubiquitination of proteins involves the concerted action of an E1 ubiquitin-activating enzyme, an E2 ubiquitin-conjugating enzyme, and an E3 ubiquitin–protein conjugate, with UB being transferred from E1 to E2, E2 to E3, and E3 to a substrate (Hellmann and Estelle 2002; Sullivan and Deng 2003; Pickart 2004; Stone et al. 2005). This study demonstrated that *Ta*E3V1, acting as an E3, physically interacts with *Ta*VRN1 as its substrate. Furthermore, *Ta*VRN1 interacted with a single UB, which has the typical lysine residues present in UB molecules from plants and other organisms (Dreher and Callis 2007; Wang et al. 2009a, 2009b; Li and Schmidt 2010). The addition of *Ta*UBV1 to wheat *Ta*VRN1 by *Ta*E3V1 should be validated in future experiments.

To explore the phenotypic consequences of the loss of *TaE3V1* in wheat, we turned to CRISPR/Cas9-mediated gene editing. A sgRNA designed against *TaE3V-B1* produced several independent editing events, with large insertions and deletions in the 3 homoeologous genes and small insertions and single nucleotide changes. This study obtained 7 edited events in the 3 homoeologous *TaE3V1* genes. The stacking and pyramiding of edited alleles in the same plant without any crosses are now possible. However, the dosage-dependent effect of *Ta*E3V1 function on the development process was unclear, as we did not observe all genetic combinations of edited genes. We did, however, observe differences in phenotypes between plants homozygous for 1 or 2 edited genes, suggesting a complex regulatory hierarchy among homoeologs and potential compensatory mechanisms. A thorough phenotypic investigation of all possible genotypes (wild type, 3 single mutants, 3 double mutants, and 1 triple mutant) with *Cas9* transgene removed will be intriguing in future studies.

The previously cloned and characterized vernalization- and photoperiod-related genes confer dramatic phenotypic differences in heading date that are as large as a few months between spring and winter wheat (for vernalization) and up to 2 mo between cultivars with sensitivity or insensitivity to photoperiod (Chouard 1960; Yan 2009; Srikanth and Schmid 2011). However, the genes in other flowering pathways tend to be associated with relatively minor effects on heading date. For example, *Ta*OGT1 in winter wheat cultivars alters flowering time by about 2 d in the field by posttranslationally adding GlcNAc to *Ta*VRT2. In spring wheat cultivars, *Ta*Col-B5, which can be phosphorylated by *Ta*K4, alters flowering time by a few days (Zhang et al. 2022a, 2022b). Although *TaE3V1* in the ubiquitination pathway changes maturity time by only a few days in controlled conditions, it is a critical regulator in the field, as an earlier maturity time can lessen exposure to the hot and dry summer season as the global climate shifts toward warmer temperatures. The natural allele and edited alleles of *TaE3V1* identified in this study represent key germplasm resources that can now be used to accelerate flowering time in wheat cultivars by several days, helping them to adapt to warming climates.

## Materials and methods

### Plant materials and growth conditions

‘Jagger’ and ‘2174’, 2 winter wheat cultivars, were crossed to generate a population of 96 F_6:7_ RILs by single-seed descent from the F_2_ generation (Chen et al. 2010). This RIL population was tested for the developmental process with and without vernalization (Li et al. 2013).

The *Ta*E3V1 editing construct was transformed into the host plant cultivar Bobwhite’. Positive T_0_ plants were self-pollinated to develop transgenic T_1_ lines that were tested in the greenhouse, where a stable temperature and photoperiod (16-h light at 25 ± 2 °C and 8-h darkness at 20 ± 2 °C) throughout the lifecycle of the plants was maintained to lessen any environmental effects on the phenotypes characterized in this study (Li et al. 2013). The *TaE3V1-*edited T_1_ plants were used to develop transgenic T_2_ lines tested in the same greenhouse. The F_2_ families were manually phenotyped for development and agronomic traits in the same greenhouse.

### The maturity index

Leaves, spikes, and peduncles were scored using a 0 to 100 scale (intervals of 10), in which 0 for plants with no maturity (>90% green tissue) and 100 for plants at the end of maturity (>90% yellow tissue). The maturity index of the T_2_ population was scored at the initiation of the flag leaf yellowness (10 to 20 score) of ‘Bobwhite’ (WT control), which started 96 d from the date of planting under the greenhouse conditions.

### Identification of genes corresponding to the sequences in the Y2H clones

The sequences of positive Y2H clones obtained using the *Ta*VRN1 bait were used as queries against the International Wheat Genome Sequencing Consortium (IWGSC) ‘Chinese Spring’ genome. The full-length coding sequences and genomic sequences for each candidate were extracted from the CS genomic sequence to determine the complete gene sequence and exon and intron structure. The coding sequences for *TaE3V1* alleles were amplified from ‘Jagger’ and cloned into a Y2H vector and compared to the CS sequences to determine allelic variants. The genomic sequences were also used as a query against 10 resequenced wheat genomes (‘Jagger’ and other 9 cultivars) to detect polymorphisms among these cultivars (GrainGenes database: https://wheat.pw.usda.gov). The predicted protein sequences for the above genes were used as queries in a BLASTP search against the NCBI nonredundant protein databases to find homologous proteins. The 3 wheat homoeologous proteins were aligned to related proteins sharing the same conserved domain from rice and Arabidopsis for *Ta*E3V1 and rice, Arabidopsis, *E. coli*, and human for *Ta*UBV1.

### A PCR marker for 2 *TaE3V1* alleles

A PCR marker was developed to detect 2 allelic variations between *TaE3V1a* from ‘Jagger’ and *TaE3V1b* from ‘2174’. The primers TaE3-MF3 5′-CCACTTCGTGACACAAACCAAACA-3′ and TaE3-MR3 5′-TCCCGCTTTGTTCTCTTCCATAA-3′ were designed to detect the SNP involving in the Cys^92^/Arg^92^ substitution. The cycling conditions were 94 °C for 3 min, 40 cycles of 94 °C for 30 s, 57 °C for 30 s, and 72 °C for 30 s, followed by a final extension at 72 °C for 5 min. The PCR products were digested with *Hha*I to produce 408 and 362 bp fragments for the ‘Jagger’ *TaE3V1a* allele but 603 and 169 bp fragments for the ‘2174’ *TaE3V1b* allele.

### Subcellular localization and colocalization analysis

For subcellular localization analysis, the full-length coding sequences (CDS) of *TaE3V-B1* were cloned into the vector pEG101-YFP, transformed into *Agrobacterium tumefaciens* strain GV3101, and infiltrated into the leaves of *N. benthamiana* plants, as described previously (Li et al. 2013). For subcellular colocalization analysis, the full-length CDSs of *TaE3V-B1* and *TaVRN1* were cloned into the pCAMBIA1300-35S-GFP and pCAMBIA1300-35S-RFP vectors, respectively, transformed into *A. tumefaciens* strain GV3101 and then coinfiltrated into the *N. benthamiana* leaves. After 72-h incubation, the fluorescence signals of both GFP and RFP were observed using a fluorescence microscope (Axio Imager.Z2, Carl Zeiss, Germany). Excitation wavelengths were 488nm for GFP and 532 nm for RFP, respectively.

### Interactions of *Ta*VRN1 with *Ta*E3V1

The full-length CDSs of *Tavrn-A1a* from ‘Jagger’ and *Tavrn-A1b* from ‘2174’ were amplified with the gene-specific primers TaVRN1-BD-F and TaVRN1-BD-R and were cloned into the Y2H vector pGBKT7. Similarly, the cDNAs coding regions encoding residue 78 to the C-terminus were amplified with gene-specific primers TaE3V1-AD-F combined with TaE3V1-AD-R1 for *TaE3V-B1b* from ‘2174’ and with TaE3V1-AD-R2 for *TaE3V-B1a* from ‘Jagger’, and the PCR products were cloned into pGADT7. The appropriate pairs of bait and prey constructs were cotransformed in the Y2H system. Positive transformants were selected for growth on a synthetic defined (SD) medium lacking Trp and Leu (SD/-Trp/-Leu). Protein interaction was determined based on the growth and blue coloration of transformants on SD medium lacking Trp, Leu, His, and Ade (SD/-Leu-Trp-His-Ade) but containing X-α-gal. All primers used for cloning in this study are provided in Supplementary Table S1.

For the LCI assay, the full-length and truncated *Ta*E3V-B1a and *Ta*E3V-B1b were fused with the N-terminal part of the luciferase (LUC) reporter, and the full-length *Ta*vrn-A1a and *Ta*vrn-A1b were fused with the C-terminal part of LUC. The resulting constructs were introduced into *A. tumefaciens* strain GV3101 and coinfiltrated into *N. benthamiana* leaves. After 60 h of cultivation, LUC activities were imaged and analyzed using the NEWTON7.0 Bio, Plant Imaging System (Vilber, France).

For the Co-IP assay, the coding regions encoding *Ta*E3V-B1a-C and *Ta*E3V-B1b-C were cloned into p1300-35S-GFP to generate the *Ta*E3V-B1a-C-GFP and *Ta*E3V-B1b-C-GFP constructs, and the full-length *Tavrn-A1a* CDS was cloned into p1300-35S-Flag to generate the *Ta*vrn-A1a-Flag construct. These constructs were transformed into *A. tumefaciens* strain GV3101 and then coinfiltrated into *N. benthamiana* leaves, which were harvested 72 h after infiltration. A Co-IP assay was performed as described previously (Ma et al. 2023). Anti-GFP-conjugated magnetic agarose beads (MBL, Japan) were used for the IP, and anti-GFP and anti-Flag antibodies (1:4,000 dilutions; ABclonal, China) were used for immunoblotting analysis.

For the BiFC assay, the full-length *Ta*E3V-B1 and *Ta*vrn-A1 were individually fused to the N- and C-terminus of YFP and transformed into *A. tumefaciens* strain GV3101, and coinfiltrated into *N. benthamiana* leaves. The YFP fluorescence signal was observed 72 h after infiltration under a fluorescence microscope (Axio Imager.Z2, Carl Zeiss, Germany).

### Immunoblot analysis of *Ta*E3V1 proteins

Total plant proteins were extracted as previously described (Jia et al. 2021). Briefly, leaves of wild-type and *TaE3V1*-edited plants, which, 2 wk after planting, were homogenized in an extraction buffer containing 50 mm Tris–HCl, pH 7.5, 150 mm NaCl, 10% (v/v) glycerol, 0.1% (v/v) Nonidet P-40, 1 mm DTT, 1 mm PMSF, and 1× protease inhibitor cocktail (Roche). After centrifugation and determination of protein concentration, the extracted total proteins (40 *µ*g per lane) were separated by 12% (w/v) SDS-PAGE and transferred onto PVDF membranes (Millipore). Immunoblot analysis was performed as previously described (Jia et al. 2021). The primary antibody was generated against the E3 protein sequence RVSLKELPSGKAAIAPSC from amino acids 134 to 151 (product number AP23031, ABclonal, Woburn, Massachusetts, United States), and a horseradish peroxidase-conjugated goat antirabbit IgG (ABclonal, Woburn, Massachusetts, United States) was used as the secondary antibody for detecting *Ta*E3V1 proteins. The signal on the membranes was developed with Clarity Western ECL substrate (Bio-Rad, Hercules, California, United States). Digital images were captured using a FluorChem System (ProteinSimple, San Jose, California, United States).

### Transcript levels of *TaE3V1* genes

Total RNA was extracted from the leaves of ‘Jagger’ and ‘2174’ plants grown in the greenhouse using RNAzol reagent (Molecular Research Center, Inc., Ohio, United States) and used to synthesize first-strand cDNA with a SuperScript II Reverse Transcriptase kit (Thermo Fisher Scientific, Waltham, Massachusetts, United States). The following primers were used to amplify the homoeologous genes: *Ta*E3V-A1F1-RT and *Ta*E3V-A1F1-RT for *TaE3V-A1*; *Ta*E3V-B1F1-RT and *Ta*E3V-B1F1-RT for *TaE3V-B1*; and *Ta*E3V-D1F1-RT and *Ta*E3V-D1F1-RT for *TaE3V-D1*. Transcript levels were determined by RT-qPCR with iQ SYBR Green Supermix (Bio-Rad Laboratories, Hercules, California, United States). The cycling conditions were 2-min polymerase activation at 95 °C and 39 cycles of 95 °C for 15 s, 64 °C for 30 s, and 72 °C for 31 s, with the addition of 4% (v/v) DMSO to the final reaction. The level of *Actin* expression was measured as an endogenous control. Transcript levels were calculated using the 2^−ΔΔCT^ method (Liu et al. 2018).

### Ubiquitination assay

The PCR products from TaE3V1-AD-F combined with TaE3V1-AD-R1 for *TaE3V-B1b* from ‘2174’ and with TaE3V1-AD-R2 for *TaE3V-B1a* from ‘Jagger’ were cloned into pMAL-c2× vector (*EcoR*I and *BamH*I sites) to express MBP-tagged proteins. The in vitro ubiquitination assay was carried out as described previously (Zhang et al. 2022a, 2022b). In brief, 0.15 *µ*g E1, 0.25 *µ*g E2, 9 *µ*g UB, and 1 *µ*g purified MBP-*Ta*E3V-B1b, MBP-*Ta*E3V-B1a, MBP-GW2, or MBP alone were incubated in a 30-µL reaction buffer containing 40 mM Tris–HCl (pH 7.5), 5 mM MgCl_2_, 2 mM ATP, and 2 mM DTT. The reaction was performed at 30 °C for 3 h and then stopped with 5× SDS-PAGE loading buffer, followed by boiling at 100 °C for 5 min. Samples (10 *µ*L) were separated by 12% (w/v) SDS-PAGE, and ubiquitinated proteins were detected by immunoblotting with an antiubiquitin antibody (R&D Systems, CAT# 104). Human E1 (UBE1, CAT# E2-305), E2 (UbcH5a, CAT# E2-616), and UB (CAT# U-100H) were purchased from R&D Systems (Minneapolis, Minnesota, United States).

For the *Ta*E3V-B1-mediated ubiquitination assay of *Ta*vrn-A1, His-tagged *Ta*vrn-A1a and *Ta*vrn-A1b recombinant proteins were expressed and purified as described (Li et al. 2013). Next, a 30-µL reaction mixture containing 0.15 *μ*g E1, 0.25 *μ*g E2, 9 *μ*g UB, 2.0 *μ*g purified MBP-*Ta*E3V-B1 (*Ta*E3V-B1a or *Ta*E3V-B1b), and 2.0 *μ*g His-*Ta*vrn-A1 (*Ta*vrn-A1a or *Ta*vrn-A1b) were incubated at 30 ℃ for 3 h. Then, the samples were separated by 12% SDS-PAGE, and the presence of ubiquitinated *Ta*vrn-A1 proteins was detected through immunoblotting with an anti-His tag antibody (ABclonal).

In vivo ubiquitination assay was performed in *N. benthamiana* leaves. *A. tumefaciens* strain GV3101 carrying *Ta*vrn-A1a-Flag and *Ta*E3V-B1a-C or *Ta*E3V-B1b-C constructs were coinfiltrated into *N. benthamiana* leaves, which were harvested 72 h after infiltration. An IP assay was performed using an Anti-Flag-conjugated magnetic agarose beads (MBL, Japan), and anti-Flag and antiubiquitin antibodies were used for immunoblotting analysis.

### Generation and effects of multiple *TaE3V1* editing events in transgenic wheat

A sgRNA was designed to target *TaE3V1* using the sgRNA Scorer 1.0.33 program, and TaE3V1-CRISPR-F (5′-GTTCATCTGCTGCCGCGCCC-3′) and TaE3V1-CRISPR-R (5′-GGGCGCGGCAGCAGATGAAC-3′) oligos were synthesized for cloning. The sgRNA sequence was used to search the IWGSC RefSeq v2.1 CS genome sequence to ensure that it has no potential off-target matches. The sgRNA sequence was cloned into the pBUN421 vector, which harbors the *Cas9* gene codon-optimized for maize (*Zea mays*) and pGTR14 (sgRNA–tRNA units) for genome editing (Zhang et al. 2022a, 2022b). The construct was transformed into cultivar ‘Bobwhite’ embryos using gold particle bombardment. Primers (TaU3-F1/R1 and zCAS9F1/R1) were used to identify positive plants with the editing construct in the wheat genome (Jia et al. 2021; Zhang et al. 2022a, 2022b; Fan et al. 2023). Specific primers were developed to amplify the genomic region covering the sgRNA target site in *TaE3V-A1* and *TaE3V-D1* from the positive plants, E3A-S1F and E3A-S1R for *TaE3V-A1*, E3B-S1F and E3B-S1R for *TaE3V-B1*, and E3D-S1F and E3D-S1R for *TaE3V-D1*. The PCR products were sequenced directly by Sanger sequencing.

### Accession numbers

Sequence data from this article can be found in the GenBank/EMBL data libraries under the following accession numbers: *TaVRN1*, TraesCS5A02G391700; *TaE3V1*, TraesCS2B02G417700.

## Supplementary Material

kiae606_Supplementary_Data

## Data Availability

All data supporting the findings of this study are available within the paper and within its supplementary data published online.

## Supplementary data

The following materials are available in the online version of this article.

**Supplementary Figure S1.** The cDNA sequences of 5 clones in the first set from the Y2H library using *Ta*VRN1 as bait.

**Supplementary Figure S2.** The gDNA sequences of 3 homoeologous genes in ‘Chinese Spring’.

**Supplementary Figure S3.** The sequences and conserved domains of proteins deduced from the cDNA sequences of the Y2H clones that were identified using *Ta*VRN1 as a probe.

**Supplementary Figure S4.** Localization of *Ta*E3V1 in *N. benthamiana* leaves.

**Supplementary Figure S5.** *TaE3V-B1* of ‘Jagger’ and ‘2174’.

**Supplementary Figure S6.** Interaction analysis of *Ta*E3V-B1 and *Ta*vrn-A1b in a LCI assay.

**Supplementary Figure S7.** Interaction analysis of *Ta*E3V-B1 and *Ta*vrn-A1b in a BiFC assay.

**Supplementary Figure S8.** The cDNA sequences of 7 clones in the second set from the Y2H library using *Ta*VRN1 as bait.

**Supplementary Figure S9.** The gDNA sequence of the gene corresponded to the sequences of 4 Y2H cDNA clones.

**Supplementary Figure S10.** Ubiquitin sequences deduced from Y2H cDNA clones.

**Supplementary Figure S11.** *In vivo* ubiquitination assay of *Ta*vrn-A1a mediated by *Ta*E3V-B1 in *N. benthamiana* leaves.

**Supplementary Figure S12.** Editing events in the *TaE3V-A1* gene.

**Supplementary Figure S13.** Editing events in the *TaE3V-B1* gene.

**Supplementary Figure S14.** Editing events in the *TaE3V-D1* gene.

**Supplementary Table S1.** Primers used in this study.

## Dive Curated Terms

The following phenotypic, genotypic, and functional terms are of significance to the work described in this paper:

HUB2 Gramene: AT1G55250
HUB2 Araport: AT1G55250

ATL6 Gramene: AT3G05200
ATL6 Araport: AT3G05200

PPD1 Gramene: AT4G15510
PPD1 Araport: AT4G15510

protein CHEBI: CHEBI:36080

VRN2 Gramene: At4g16845
VRN2 Araport: At4g16845

CDS Gramene: Coding sequence
CDS Araport: Coding sequence
