# Supplementary material for: The E3 ligase TaE3V-B1 ubiquitinates proteins encoded by the vernalization gene TaVRN1 and regulates developmental processes in wheat
Source: Plant Physiol. 2024 Nov 18;197(1):kiae606. doi: 10.1093/plphys/kiae606 (PMC11663705; doi:10.1093/plphys/kiae606)
Supplement: kiae606_Supplementary_Data [file kiae606_supplementary_data.zip › suppdata.docx]

>LY691-T7 sequence exported from chromatogram file

CCCACAAAACGATGGCATCACATTACGACGTACCAGCTTACGTTTAATATGGCCTGCAGGCCGTGAATTCCACCCAAGCAGTGGTATCAACGCAGAGTGGCCATTATGCCGGGTCTCGTTGAAAGAGTTGCCTAGCGGCAAAGCTGCCATAGCACCTTCATGTAGCAATCCTCAAGTGCGCCCCCGCACTGAGAACTCTGTTAATCCAGCACCTGACTGGCTCCTCCCTGTTCATCATTCTCACAGAGGTCAACAGAATAGTTCAGACACACAAGCATCAGTAGAAGTGGTTATTGAGATACGCCAATAAGCACATCATGAGATTGCTATGAAAGAGAACAAAGCGGGAATATGTAATAGGTTTCCTGCCTCGCTGCATTGTTGCAGCATCCTAATTGGATCGACATTGTATGCAATCTCGTCGCAGGTAATGTGTCAACATTTGTTGTACATTTCGCATTGTAGGTAAGCATATTGTGTTATGGCACATAAACACTTTCAATGTTCTTTTCTAGTGCTCTGTATATCATAAAAATGGCAAAAAAAAAAAAAAAAAAAAAAA

>LY701-T7 sequence exported from chromatogram file

CGCCAGCCCGCCTGGAATACCCTACGACGTACCGATTACGCTCATATGGCCTGGAGGCTAGTGAATTCCACCCAAGCAGTGGTATCAACGCAGAGTGGCCATTATGCCGGGTCTCGTTGAAAGAGTTGCCTAGCGGCAAAGCTGCCATAGCACCTTCATGTAGCAATCCTCAAGTGCGCCCCCGCACTGAGAACTCTGTTAATCCAGCACCTGACTGGCTCCTCCCTGTTCATCATTCTCACAGAGGTCAACAGAATAGTTCAGACACACAAGCATCAGTAGAAGTGGTTATTGAGATACGCCAATAAGCACATCATGAGATTGCTATGAAAGAGAACAAAGCGGGAATATGTAATAGGTTTCCTGCCTCGCTGCATTGTTGCAGCATCCTAATTGGATCGACATTGTATGCAATCTCGTCGCAGGTAATGTGTCAACATTTGTTGTACATTTCGCATTGTAGGTAAGCATATTGTGTTATGGCACATAAACACTTTCAATGTTCTTTTCTAGTGCTCTGTATATCATAAAAATGGTAAAGAAATATTGGATGTTAGGTAAATTCCTCAAAAAAAAAAAAAAAAAAAAAA

>LY707-T7 sequence exported from chromatogram file

GGGGAGGCCGACTGGTATCCCTACGACGTACCGATTACGCTCATATGGCCTGGAGGCCGTGAATTCCACCCAAGCAGTGGTATCAACGCAGAGTGGCCATTATGCCGGGTCTCGTTGAAAGAGTTGCCTAGCGGCAAAGCTGCCATAGCACCTTCATGTAGCAATCCTCAAGTGCGCCCCCGCACTGAGAACTCTGTTAATCCAGCACCTGACTGGCTCCTCCCTGTTCATCATTCTCACAGAGGTCAACAGAATAGTTCAGACACACAAGCATCAGTAGAAGTGGTTATTGAGATACGCCAATAAGCACATCATGAGATTGCTATGAAAGAGAACAAAGCGGGGATATGTAATAGGTTTCCTGCCTCGCTGCATTGTTGCAGCATCCTAATTGGATCGACATTGTATGCAATCTCGTCGCAGGTAATGTGTCAACATTTGTTGTACATTTCGCATTGTAGGTAAGCATATTGTGTTATGGCACATAAACACTTTCAATGTTCTTTTCTAGTGCTCTGAAAAAAAAAAAAAAAAAAAAAAAAAAAAAAAAAAAAAAAAAAAAAAAAAA

>LY709-T7 sequence exported from chromatogram file

TCGACAGCGCCTGGTATACCCTACGACGTACCGATTACGCTCATATGGCCTGGAGGCCAGTGAATTCCACCCAAGCAGTGGTATCAACGCAGAGTGGCCATTATGCCGGGTCTCGTTGAAAGAGTTGCCTAGCGGCAAAGCTGCCATAGCACCTTCATGTAGCAATCCTCAAGTGCGCCCCCGCACTGAGAACTCTGTTAATCCAGCACCTGACTGGCTCCTCCCTGTTCATCATTCTCACAGAGGTCAACAGAATAGTTCAGACACACAAGCATCAGTAGAAGTGGTTATTGAGATACGCCAATAAGCACATCATGAGATTGCTATGAAAGAGAACAAAGCGGGAATATGTAATAGGTTTCCTGCCTCGCTGCATTGTTGCAGCATCCTAATTGGATCGACATTGTATGCAATCTCGTCGCAGGTAATGTGTCAACATTTGTTGTACATTTCGCATTGTAGGTAAGCATATTGTGTTATGGCACATAAACACTTTCAATGTTCTTTTCTAGTGCTCTGTATATCATAAAAATGGCAAAAAAAAAAAAAAAAAAAAAAA

>LY736-T7 sequence exported from chromatogram file

GCAGCCAACCGACTGGTAAACCCTACGACGTACCGATTACGCTTATATGGCCTGGAGGCCAGTGAATTCCACCCAAGCAGTGGTATCAACGCAGAGTGGCCATTATGCCGGGTCTCGTTGAAAGAGTTGCCTAGTGGCAAAGCTGCCATAGCACCTTCATGTAGCAACCCTCAAGTGCGCCCCCGCACTGAGAACTCTGTTAATCCAGCACCTGACTGGCTCCTCCCTGTTCATCATTCTCACAGAGGTCAACAGAACAGTTCAGACACACAAGGATCAGTAGAAGTGGTTATTGAGATACGCCAATAAGCACAGCATGAGATTGTTATGGAAGAGAACAAAGCGGGAATATGTAATAGGTTTCCTGCCTCGCTGCATTGTTGCAGCATCCTAATTGGATCGGCATTGTACGCAATCTCGTCGCAGGTAATGTGTCAACATTTGTTGTACATTTCGCATTGTATGTAAGCATATTGTGTTATGGCACATAAACACTTTCAATGTTCTTTTCTAACGCTCTGTATATCGTAAAAATGGTAAAGAAATATTGGATGTTAGAAAAAAAAAAAAAAAAAAAAAAAAAAA

**Supplementary Figure S1.** **The cDNA sequences of five clones in the first set** **from the Y2H library using *Ta*VRN1 as bait.** The first four clones with different lengths corresponded to the gene *TraesCS2A02G399700* on chromosome 2A. The last one corresponded to the homoeologous gene *TraesCS2B02G417700* on chromosome 2B. The poly‘A’ sequences in the cDNAs are highlighted in yellow.

>TraesCS2A02G399700

ATGATGTTCGGGTCGGGGATGAATCTCCTCAGCGCGGCGCTCGGCTTCGGCATGACCGCCGCCTTCGTCGCGTTCGTCTGCGCGCGGTTCATCTGCTGCCGCGCCCGGGACGCGGACGATGGCGCCCCATCGCCGGTGGACTTTGACGTTGACTTCCCAGCAGATCTCGAACGCCCGGTGCGTGTCATACCCTCCTACTATGGCTCCTCTGCCTCCACAACGATCGATATTCTGCTGCTATGAACTACGTATTTGCTAGTGCCACAGCGCGTACATAACTGAAGTGTCTATGTTTTGTTTTTATAATTACTGAAAATAGGAAGATTCGATACTCAATTTCTTTGTTCGCGGGCACATGAGCAGTGCCTTTTATTTTTGGCTATTACTGAAGTGTATATGTTTTGTTTTTATATAGTTATTAGTGATGAAAATAGGAAGATTCTAGGAGTACTATTTTATATCTGTGTTCATGGGCACATGGGAGTGCCTTTCCTGTTCAAGCCAAAAAAAAATTGCTAATTAGTCCTTTTACTAAATTTGTACTTTTTGGCTATTGCACTGATACCAAGTCTTAAATATTAGTATATCTGATAATCGACCACTTTAATAGATATTAGAAAATAGAGATTCAAAAGCTTTAGCTTATATGTATTTCGCTAACTTTGGTCTTCTCCCATTTTAGACTGGTATTTGTGTGCCTCTGTTTTAGTGATAGGCCATGAGCTCTAGTTAGATTCTTAGGGTTACACTTTCCCCAATAATTGTCTCTTGATCGCATACCTTACAGTATCTCTGTATCAGCTTTCAATTAGTCTGTGTATAACTACAAGCCTGCAACGTAGCAATATTCCTTGGTCCAAGTTCAAATAACAGTTAAATCATCCTTAGTCCCTACACATTTAAAGAAGTAAATAGGTTTAAATGACTAGGCGATGAGCTCTAGTTAGATTCATGAGGTTACATTTTACCCCAATAATAGTTGCTCAATACGTAGCTTGTATCTTTGTTTCTGCTTCAAGTTAGTCTGTGTATGACTAGAAGCCTGCAACTTAACAATATTCCCTGGTCCAAATATAAATAACAGTTACATGATCCTTATTCCTTGCACATTTTAAGAAGGAAGAAGATATCACATTCTAATTCACAGTCTAAAACCAATGCGTTTTGTAGTCCCACAAGGAAATACTAATAAGCTAGAAACTGAAACACCAAAGGGTGGGAGCACTAAAGGAACTACAGAAATCCCAGATTTTGTATGATGTCTTTAAGCTATAATCTATTGGATAATTTGCTAGCTTGGATACAAATTCAACAATGCTCTTTGATGATACCCGTCAGTTCAGCCTCGTTTCTTTAGGGTTAAAATTTTGGGCATCTCCCTTTTTGAATCCTATATACCTAAATAGTTCCCCACTAACTCAATTTTCTTAACATGCAACTATGCCAACTCACCATGCCACCATGCACGCCCCACTAAATTTAGTTCTCTCAACATGCAACTATGCCAACTCACCCTGACACTATGCATGGGAAAAGACCCACCTTAACATGTAGCTTGCATGCTACTCATATTCAATAAATATTTTACAACTATATAATGATCAAACATAATAAATTATATGTCCTTTAGTTTCGATTCTCATATCACCAATCCAAATACTAAAGTTTTATACTGCCAATTTTCGTAGAAATAGCTGCATCCAATTCCCGCAGCAATGCGCGGGGTATCGTCTCTAGTAAACTTGATAACTAAGTGCACATGGAGTATTCTCTGTTCCTTGGTATAGAATTTTATTTTCGCAAATGTCAAGTGAACAAACATTTCTTATGAAGTTGCTCTTCCCAGTTGTACAAGGTGTCTCCTTTTGAAAGTTAATGTAGTGATCGCTGTTATGTTTCGCGCCTCTGGGCTAGGGCCGGCGCCAACGCGATCAGTAACATAATCCCGGAGAACTAGGGTAGGATGTAGAGGGGATGAGCAACCCCATCTGTTTGCTCGGCGGCCACTTCCATGCCTTCCTTGTGTAGATGGCATAGTGCGTTGTCACAACCTCATCTGTACAACTCCCCTTCTATCAATGGAAAGATACGCATCCTTAGCGTATTCGCGAAAAAAGATAGCTGTTATGTTTATAGTTATGTACGCATAGTTGACTACCTTTTTTTTTCCTTTACTGTTACATCTAAAAGTGTTACTTGTTAGTGTATGTTCAACAAATACTTTTTTAATTCACATGCAAGTTTGAACAATCAAGGCGATGTAGTCAATGCAGATGTCATATCACTATCTTTCGTACTTAAGTTAGTGCCGTACTTTTTCAGGTAGAGGATGCTAATTGTGGGTTGGAACCTTTGGTTATTGCTGCAATTCCTATTATGAAGTACTCTGAGGCTTTATATTCAAAGGATGATGCCCAGTAAGTGCAACTATCTGTATTAACCGACAATTACCCAACTTTACTGTTTTCTGCTGTAATGTATATAATTTCATCCACCTCTGAAATGTCTGGCCACTGGGATTCATGGGAATATCCAAACAGTGGGCCAAGTAGCTTGTCACATGGAAATTGGTAGACCCTGTTGCAGTATCACCTTATTGTAAATACGGGTATCCAGAATAGTTTCTAAACAGTTATGTGCGAGTTATTGAAACCACATTACTGCTGATATGCATATGCTGAGACTCTTAGAAAGTCACAAGAGTCTTTTCTTTTCTTTGTTGTCATATAGTCCCTAGCCCCATGGTTATCAATTCAATATACCAGTTCCTATGGCCCCCCTCCCCTCCCCGAGCATTTTGAAGCTTACCTGGTAATTTCATTAACCAAAGCATGAGAAAGTATCTGCAAAATGTGTATGCATTTCTGGGGGTGCAGGTCAAAGATCTGCTATGAAGGACATGTTTTATGCTGAAAGAATACTTCCATAGCTGAGTAACAAATGTACAAAATAATTTGAAATCTTGCTGTTAGCTAGAGCAAGTTCATAATGCCAGTTTGTTTTTGCTTAGTCCTTCGAGGAAGGAATGTGTTTTCCTGCCCAGGGGTTCCCACGCCTTCTGTTTGCAAAATAAATAATGTCTTGTTAAAAAAAATACCAATCATGTGTGATCCACAAAACTTTGTTTCTGTGTAAACCTGCAGACTTTTAATATCAAAATTGTCCATACTGTGCGGTACGAAAAATAGTAGTGAACCATCCACTGTATTAATCAGTGCAAGTGAAGTGTCTTTTATCGTATTTGGTTCTTGATACCAGAAATGAATTTGATATTGAAATGTTGCAAGTCTACATAACTTTATTGTGTACTACACTACACACATGGTTAATTTTTATTTGAATTACAAGTACCAGCGCTAACACACCCGCCCCGCTTAAACCACCATACCTATGCACACACACGCCCCATTGTTTACCACTGAACAGGTATTGCTGTGAAGATATGACACACGAACTCTCGATCCAAGGACTGAACCCGGCTGGCAGGCTGCAGCACGTCAATCCTAACCAACGAGACATGGCTTATTTCACACATAATTGAGAAAAACTGCGGTTTTTAAAATCTAAAGTGTTCATTGTGAGGCATTGGAATACTGAGGTATTAGATTACAGCAATTTTCCAAACCTAAGTTTTGATACAGTGCATCATGCACTTTCTGTAAATGGGCGTGCAGGAACTCTGGGGAGCAATGAAATTGTTGAACTGTTAAGGCCATGTTGGGGTTTGCGTTTCTGCTCCATTTCTCGCCGGTTTAGAATACACACCCCGTCCCGTCGTTTTCGTTCTAGGCAAAAAACAAGTGTTGACCAGTAATTTACACCTGTAAAATAAATACAAGTGTATAAATTTACACCCATTCCAAGTGGGGCCTAAGAAGTTTTTACACAAGCAAGTCTATTGCCCTCCAAACTTGTTTGCATAGACTCAACCAAGCTCAAACTCAGCCCAATGTTTAGAAGCTCACAAGACATGAGTTCTTTCTTAACAGTTCTTCTCTTTATTCAACTTATTGAACCTAGGGTGTTGTTCAACTATGCCCTTGTTTGATCGGTTGTTTTGCTTTGACAGGTGCTCCATATGTCTAGGTGAATACACTGAGAAAGAGCTTCTAAGAATCATTCCGACATGTCAACATAACTTTCACCGTACCTGCTTAGACTTATGGTTGCAGAAGCAGACTACTTGCCCAATATGCCGGGTCTCGTTGAAAGAGTTGCCTAGCGGCAAAGCTGCCATAGCACCTTCATGTAGCAATCCTCAAGTGCGCCCCCGCACTGAGAACTCTGTTAATCCAGCACCTGACTGGCTCCTCCCTGTTCATCATTCTCACAGAGGTCAACAGAATAGTTCAGACACACAAGCATCAGTAGAAGTGGTTATTGAGATACGCCAATAA

>TraesCS2B02G417700

ATGATCTTTGGGTCCGGGCTGAATCTCCTCAGCGCGGCGCTCGGCTTCGGCATGACTGCCGTCTTCGTCGCGTTCGTCTGCGCGCGGTTCGTCTGCTGCCGCGCCCGGGGCGCGGACGACAGCGCCCCATCGCCTGTGGACTTTGACGTTGACTTCCCGGCAGATCTCGAACGCCCGGTGCGTGCCATACCCTACTATGGCTCCTCTGCCTCCACACCGATATTCTGCTGCTGTGACCTCCACGCCGTTTATTTGCTAGTGCTACAGCGCGTACATAACTGAAGTGTCTATTTTTTGTTTTTATAATTACTGAAAATAGAAAGATTCGATACTCAATTTCTTTGTTCGTGGGCACATGGGCAGCGCCTTTTAATGTTGGCTATTACTGAAGTGTATATGTTTCGTTTTTATAGTTACTGATGAAAATAGGAAGATTCTACTATTTTATATCTGTGTTCATGGGCACATGGGAGTGCCTTTCCTGTTCAAGCCAAAAAAAAAACCCTGCTAATTAGTCCTTTTACTAAATTTGTACTTTTTGGCTATTGCACTCATACCAAGTCTTAAATATTAGTATATCTGATAATCAACCACTTTAATAGATATTAGAAAATAGAGATTCAAAAGCTTTAGCTTATACTCCCTCTGACCCATAGTATAAGAACGTTTTTGACACTACACTAGTGTCGTTATTATGGGACGGAGGGAGTATGTATTTCGCTAACTTTGGTCTTCTCCCATTTTAGACTGGTACTCCCTTCGTTCCAAAATAGATGACCCAACTTTGTACTAAAGTTAGTACAAAGTTGAGTCATCTATTTTGGAACGGAGGGAGTATTTGTGTGCCTCTGTTTTAGTGATAGGCGATGAGCTCTAGTTAGATTCTTAGGGTTACACTTTCCCCAATAATTGTCTCTCGATCGTGTACCTTACAGGATCTCTGTATCTGCTTTCAATTAGTCTCTATAACTACAGGCCTGCAACGTAGCAATATTCCTTGGTCCAAATTCAAATAACAGTTAAATGATCCTTAGTCCCTACACATTTAAAGAAGTAAATAGGTTTAAATGACTAGGCGATAAGCTCTAGTTAGAGTCATGAGGTTACATTTTACCCCAATAATAGTTGCTCAATACGTAGCTTGTAGTATCTTTGTTTCTGCTTCAAGCCTTCAAGTTAGTCTGTGTATGACTAGAAGCCTGCAACTTAACAATATTCTCTGGTCCAATATAAATAACATTTACACGATCTTTATTCCTTGCACATTTAAAGAAGAAGATATCACATTCTAATTCACAGTCTAAAACCAATGTGTTTTGTAGTCCCACAAGGAAATAATAAGCTCGAAACTGAAACACCAAAAGGTGGGAGCACTAAAGGAACTACATAAATCCCAGATTTTGTATGATGTCTTTTAAGCTATCATCTATTGCATAATTTGCTAGCTTGGATACAAATTCAACAATGTTCTTTGATGATACCCGTCAGTTCAGCTTCGTTTCTCTTGGGTTAAAATTCTGGGCATCTCCCTTTTTGAATCCTATATACCTAAATAGTTCCCCATTAACTCAATTTTCTTAACATGCAACTATGCCAACTCACCATGCCACCATGCATGCCCCACTAAATTTAATTCTTTCAACATGCAACTATGCCAACTCACCCTGACACTATGCATGGGAAAAGACCCACCTTAACATGCAGCTTGCATGCTACTCATATTCAATAAATATTTCACAACTATATAATAATCAAGCATAATAAATTATATGTGCTTTAGTTTTGATTCTCATATCGCCAATCCAAATACTAAAGTTTTATACTGCCAATTATCATAGAAATAGCTGCATCCAATTCCCACAGCAATGCGCGGGGTATCGTCTCTAGTAAACTTGACAACTAAGTGCACATGGAGTATTCTCTGTTCCTTGGTATAGACTTTTATTTTCGCAAATGTCAAGTGAACAAACATTTCTTATGAAGTTGTTCCCAGTTGTACAAGGTGTCTCCTTTTGAAAGTTAATGTAGTGATAGCTGTTATGTTTATAGTTATGTACGCATAGTGACTACCTTTTTTTTCTTTACTGTTACATCTAAAATTGTTAGACTTGTTAGTGTATGTTCTACAAATACTTTTTTAATTCACATGCAAGTTTAAAATATCAAGGCGATGGTCAATGCAGATGTCATATCACTATCTTTCGTACTTAAGTTAGTGCCATACTTTTTCAGGTAGAGGATGCTAATTGTGGGTTGGAACCTTTGGTTATTGCTGCAATTCCTATTATGAAGTACTCTGAGGCTTTATATTCAAAGGATGATGCCCAGTAAGTGCAACTATCTGTATTAACCCATAATTACCTAACTTTACTGTTTTCTTCTCTAATGTATATAATTTCATCCACCTCAGAAATGTCTGGCCACTGGGATTCATGGGAATATCCGAACAGTGGGCCAAGTAGCTTGTCACATGGAAATTGGTAGACCCTGTTGCAGTATCTCCTTATTGTAAATACGGGTATCCAGAATAGTTTCTAAACGGTTGTGTGCGAGTTATTGAAACCACATTACTGCTGATATGCATATGCTGAAACTCTTAGAAAGTCACAAGATATTTTCTTTTCTTTGTTGTCATATAGTCCCTAGCCCCATGGTTATCAATTCAATATACCAGTTCTTATACCCCCGCCCCTCCCCTCCCCGAGCATTTTAAAGCTTACCTGCTAATTTCATTAACCAAAGCATGAGAAAGTATCTGCAAATGTGTATGCATTACTGGGGATGCAGGTCAAAGATCTTTTATGATGGACACATTTTATGCTGAAAGAATACTTCCATAGCTGAATAACAAATGTACAAAATAATTTGAAATCTTGCTGTTAGCTAGAGCAAGTTCATAATGCCAGTTTGTTTTTGTTCAGTCCTTCTAGGAAGGAATGTGTTTTCCTGCCCAGGGTTTTCCACGCTTTCTGTTTGCAAAATAAATAATGCCTTGTTAAAAAAATACCAATCGTGTGTGATCCGCTTAACTTTGTCTTTGTGTAAACCTGCATACTTTTGATGTCAAAATTGTCCATACTGTGCAGTACGAAAAATAGTACTGAACCACACACTATTAATCAGTGCAAGTGAAGTGTCTCTTATTGTTTTTGGTTCTCGATACCAGAAATGAATTTGATATTGAAATGTTGCAAGTCTACATAACTTTATTGTGTACTACACTACACACATGGTTAATTTCTATTTGAATTACAAGTACCAGCGCTAACACACCCGCCGCACTGAAACCACCATATCTATGCACACACACGCCCCACTGTTTACCACAGAAGGGGTATTGCTGTGAAGGTATGACACACGAACTCTCGATCCAAGGATTGAACCCGGCTGGCAGGCTGCACCACGTCAATCCTAACCAATGAGACATGGCTTATTTCACACATAAATTGAGAAAAACTGCGGTTTTTGAAAACTGAAGTGTTCATTGTGAGGCATTGGAATACTGAGGTTTTAGATTACAACAGTTTTCCAAGCCTAAGTTTTGGAACAGTGCATCATGCACTTTCTGTAAGGGTGTGTTTGTTTTGGGAATGGAGTGGAATGGAATGTCATGGTTCCATTCCACTAGAATGGGTCGATTCCATCCTTGTGTTTGGTAGGAGCAATTCGAAAGAATGGAATGGTTACATTTGATGTTTGGTTTTATAGATAGAACGGAATGGGTTTGTTCATATCTCCACTTGTTTTCTTGTGATTATGTACAATATGTTGATCTTTGGCACCAAACACAAACAAGACACATATAGAGGTAAAAAAGATCATCAACACACAGCAAAACTTTCAATTGTAAATGCCCAAAACAAGTGATATATATTGCCCTGTCAGCTATCAGCCACACTTAGCCGTAGAACTTCATCGCTTTTCTGTCATCTGCATACAAAACATAAAATTCAGTGGGAGCACAGAAAGCACCAGCTGGAGCAGCACGACACACAACAATAGGAACAAGTTCAGGCCATGAGCAGCAAGGGCCGGCGACGAGCAGGACGCACGCAAGGGCTGGCTAGCGACAGCAGTAGCAGCACGGGACACAACAACAATAGCAGCAAAGGGCGGCCACGAGCAGGACACACGCGGGGCCGGCCATGAGGGGGCAGTGGGGGAAGGTGAGCGGAGGACCTACCGGCTAGATGGACGCTGCCGTCGTCGGACCTTGACGGATCTGACGGGAAGGGGAGGGAACGGCCACGATCCGGTCGTCGCCGCCGGCGTCAGCGGCGAGTGTGCCGGCGGTGAGGCTGGGAGCAGCGAGTGTCTCCTTCGGGCGCCGCCGTTCGTCTCGTTCGGGCGCCGCCGCCCGCTGCCTGCCAGCTGTGAGGGAGATGCGAGAGAGGGAGATACGCGAGGCGCTGAGGAAGACGCGAGGGCGAGTCGTTCCGCGCGGTCCGCTCGATTCGGAGGAATTGCTCGGTTCCGCATAAACGAGGAATATGCCGTTCCCTGGAATCTGTGGGTTCCGGTCCTTTGACGATCCGAACACGCGAATGGGTCTTGGGAACGGAACCGACCCGTGCCGTTCCACTTCGTGACACAAACCAAACACACCCTAAATGAGCGTGCAGGAACCTCTGGGGAGCAATGAAATGGTTGAACTGTTAAGGCCCTGTTTGGATTCGCTTTTCTGCTCCGTTTCTCGCCGGCTTAGAATACACACCTCGTCCCATCGTTTTCGTTCTAGGCAAAAAAACAAGTGTTGACCAGTAATTTACACCCATTCCAAGCAGGGCCTAAGAAGTTTTTACACAAGCAAGTCTATTGCCCTCCAAACTTGTTTGCACAGACTCAACCAAGCTCAAACTCAGCCCAAGATTTAGAAGCTCACAAGACATGAGTTCTTTCTTAACAGTTCTTCTCTTTATTCAACTTAAACCTAGGGTGTAGTTCAACTAGGCCCTTGTTTGATCGGTTGTTTTGCTTTTACAGGTGCTCCATATGTCTAGGTGAATACACCGAGAAAGAGCTTCTAAGAATCATTCCGACATGTCGACATAACTTTCACCGTACCTGCTTAGACTTATGGTTGCAGAAGCAGACTACTTGCCCAATATGCCGGGTCTCGTTGAAAGAGTTGCCTAGTGGCAAAGCTGCCATAGCACCTTCATGTAGCAACCCTCAAGTGCGCCCCCGCACTGAGAACTCTGTTAATCCAGCACCTGACTGGCTCCTCCCTGTTCATCATTCTCACAGAGGTCAACAGAACAGTTCAGACACACAAGGATCAGTAGAAGTGGTTATTGAGATACGCCAATAA

>TraesCS2D02G397200

ATGATCTTCGGGTCGGGGCTGAATCTCCTCAGCGCGGCGCTCGGTTTCGGCATGACCGCCGTCTTCGTCGCGTTCGTCTGCGCGCGGTTCATCTGCTGCCGCGCCCGGGGCGTGGACGACGGCGCCCCGACGCCGGTGGACTTTGACGTTGACTTCCCGGCAGATCTCGAACGCCCGGTGCGTGTCATACCCTCTACTACTATGGCTCCTCTGCCTCCACACCGATATTCTGCTGCTGTGAACTCCACGCCGTTTACAAAAGGGGAACACATAACTACGCATTTGCTAGTGCCACAGCGCGTACATAACTGAAGTGTCTATGTTTTGTTTTTATAATTACTGAAAATGGAAATATTCGATACTCAATTTCTTTGTTCGTGGCCACATGAGAAGCGCCTTTTATTTTTGGCTATTACTGAAGTGTATATGTTTTGTTTTTATATAGTTATTACTGATGGAAATAGGAAGATTCTAGGAGTACTATTTTATATCTGTGTTCATGGGCACATGGGAGTGCGTTTGCTGTTCAAGCCAAAAAAAAAAACTGCTAATTAGTGCTTTTACTAAATTTGTACTTTTTGGCTATTGCACTCATACCAAGTCTAAAATATTAGTATATTTGATAATCGTCCACTTTAATAGATATTAGAAAATAGAGATTCAAAAGCTTTAGCTTATATGTATTTCGCTAACTTTGGTCTTCTCCCATTTTAGACTGGTATTTGTGTGCCTCTGTTTTATTGACAGGCGATGAGCTCTAGTTAGATTCTCAGGGTTACACTTTCCCCAATAATTGTCTGTCGATCGTGTACCTTACAGTATCTCTGTATCTGCTTTCAATTAGTCTGTGTATAACTACAAGCCTGCAACGTAGCAATATTCCTTGGTCCAAATTCAAATAACAGTTAAATGATCCTTAGTCCCTACACATTTAAGGAAGGAAATAGGTTTGAATGACTAGGCGATGAGTTCTAGTTAGATTCATCAGGTTACATTTTACCTCAATAATAGTTGCTCAATACGTAGCTTGTAGTATCTTTGTTTCTGCTTCAAGTTAGTCTGTGTATGTGTGACTAGAAGCCTGCAACTTAACAATATTCCCTGGTCCAAATATAAATAACAGTTACACGATCCTTATTCCTTGCACATTTAAAGAAGGAAGAAGATATCACATTCTAATTCACAGTCTAAAACCAATGTGTTTTATAGCCCCACAAGGAAATAATAAGCTAGAAACTGAAGCAGCAAAAGGTGGGAGCACTAAAGGAACTACATAAATCCCAGATTTTGTATGATGTCTTGCTATCATCTATTGGATAATTTGCTAGCTTGGATACAAATTCAACAATGTTCTTTGATGATACCCGTCAGTTCAGCCTCGTTTCTCTTGGGTTAAAATTTTGGGCATCTCCCTTTTTGAATCATATATACCTAAATAGTTCCCCACTAACTCAATTATCTTAACGTGCAACTATGCCAACTCACCATGCCACCATGCATGCCCCACTGAATTTAATTCTCTCAACATGCAACTATGCCAACTCACCCTGACACTATCCATGGGAAAAGACCCACCTTAACACGCAGCTTGCATGCTACTCATATTCAATAAATATTTTACAATTATATAATAATCAAACATAATAAATTATATGTGCTTTAGTTTCGATTCTCATACCGTCAATCCAAATACTAAAGTTTTATACTGCCAATTCTCATAGAAATAGCTGCATCCAATTCCCGCAGCAATGCGCGGGGTATCGTCTCTAGTAAACTTGACAACTGAGTGCACATGGAGTATTCTCTTTTCCTTGGTATAGACTTTTATTTTCGCAAATGTCAAGTGAACTAACATTTCTTATGAAGTTGCTGTTCCCAGTTGTACAAGGTGTCTCCTTTTGAAAGTTAATGTAGTGATAGCTGTTATGTTTATAGTTATGTACACATAGTTGACTACCTTTTTTTTTCTTTACTGTTACATCTAAAATTGTTACTTGTTAGTGTATGCTCAACAAATACTTTTTTAATTCACATGCAAGTTTGAACAATCAAGGCGATGTAGTCAATGCAGCTGTCATATCACTATCTTTCATACTTAAGTTAGTGCCATACTTTTTCAGGTAGAGGATGCTAATTGTGGGTTGGAACCTTTGGTTATTGCTGCAATTCCTATTATGAAGTACTCTGAGGCTTTATATTCGAAGGATGATGCCCAGTAAGTGCAACTATCTGTATTAACCGATAATTACCTAACTTTACTGTTTTCTGCTCTAGTATATATAATTTCATCCGCCTCTGAAATGTCTGGCCACTGGAATTCATGGGAATATCCAAACAGTGGGCCAAGTAGCTAGTCACATGGAAATTGGTAGACCCTGTTGCAGTATCACCTCATTGTAAATACTGGTATCCAGAATAGTTTCTAAACAGTTATGTGCGAGTTATTGAAACCACATTACTGCTGATATGCATATGCTGAAACTCTTAGAAAGTCACAAGAGTCTTTTCTTTTCTTTGTTGTCATATAGTCCCTACTCCCATGGTTATCAATTTAATATACCAGTTCTTATACCCCCCCCCCCCCCCTCCCCTCCCCGAGTATTTTGAAGCTTACCTGGTAATTTCATTGACCAAAGCATGAGAAAGTATCTGTAAATGTGTATGCATTTCTGGGGATGCAGGTCAAAGATCTGCTATGAAGGACACGTTTTATGTTGAAAGAATACTTCCATAGCCGAGTAACAAATGTACAAAATTATTTGAAATCTTGCTGTTAGCTAGAGCAAGTTCATAATGCCAGTTTGTTTTTGCTCAGTCCTTCTAGGAAGGAATGTGTTTTCCTGTCAGGGGTTCCCATGCCTTCTGTTTGCAAAATAAATAATGTCTTCTTAAAAAAGTACCAATCATGTGTGATCCACATAACTTTGTCTTTGTGTAAACCTGCAGACTTTTAATGTCAAAATTGTCCATACTGTGCAGTACGAAAAATAGTAGTGAACCACACACTGTATTAATCAGTGCAAGTGAAGTGTCTCTCTTATCGTTTTTGGTTCTCGATACCAGAAATGAATTTGATATTGAAATGTTGCAAGTCTACATAACTTTATTGTGTACTACACTACACACATGGTTAATTTTTACTTGAATTACAAGTACCAGCACTAACACACCCGCCCCACTCAAACCACCATACCCTATGCACACACACGTCCAGTGTTTACCATTGAACCAGTATTGCTGTGAAGGTATGACACACGAACTCTTGATCCAAAAGGATTGAACCCGGCTGGCAGGCTGCACCACGTCAATCCTAACCAATGAAACATGGCTTATTTCACACATAAATTGAGAAAAACTGCAGTTTTTGAAACTGAAGTGTTCATTGTGAGGCATTGGAATACTGAGGTTTTAGATTACAACAGTTTTCCAAACCTAAGTTTTGGAACAGTGCATCATGCACTTTCTGTAAATGGGCGTGCAGGAACCTCTGGGGAGCAATGAAATTGTTGAACTGTTAAGGCCCTGTTTGGATTCGCTTTTCTGCTCTGTTTCTCTCCTGTTTAGAATACACACCTCGTCCTGTCATTTTCGTTCTAGGAAAAAAACTAAGTGTTGACCAGTAATTTACACCTGTAAAATAAATACAGGTGTATAAATTTACACCCATTCCAAGCGGGGCCTAAGAAGTTCTTCACAAGCAAGTCTATTGCCCTCCAAACTTGTTTGCACAGACTCAACCAAGCTCAAACTCAGCCCAATATTTAGAAGCTCACAAGGCATGAGTTCTTTCTTAACAGTTTCTTCTCTTTATTCAACTTAAACCTTGGGTGTTGTTCAACTATGCCCTTGTTTGATCGGTTGTTTTGCGTTGACAGGTGCTCCATATGTCTAGGTGAATACACTGAGAAAGAGCTTTTAAGAATCATTCCGACATGTCAACATAACTTTCACCGTACCTGCTTAGACTTATGGTTGCAGAAGCAGACTACTTGCCCAATATGCCGGGTCTCGTTGAAAGAGTTGCCTAGCGGCAAAGCTGCCATAGCACCTTCATGTAGCAACCCTCAAGTGCGCCCCCGCACTGAGAACTCTGTTAATCCAGCACCTGACTGGCTCCCCCCTGTTCATCATTCTCACAGAGGTCAACAGAATAGTTCAGACACACAAGGATCAGTAGAAGTGGTTATTGAGATACGCCAATAA

**Supplementary Figure S2.** **The gDNA sequences of three homoeologous genes in Chinese Spring.** These sequences corresponded to five cDNA clones (Supplemental Figure S1) identified from the Y2H library. The start codon, the stop codon, and the splicing sites of the intron are highlighted in yellow and red letters. The exon sequences are highlighted in grey.

**A**

>TraesCS2A01G399700.1

MFGSGMNLLSAALGFGMTAAFVAFVCARFICCRARDADDGAPSPVDFDVDFPADLERPVEDANCGLEPLVIAAIPIMKYSEALYSKDDAQCSICLGEYTEKELLRIIPTCQHNFHRTCLDLWLQKQTTCPICRVSLKELPSGKAAIAPSCSNPQVRPRTENSVNPAPDWLLPVHHSHRGQQNSSDTQASVEVVIEIRQ*

>TraesCS2B01G417700.1

MIFGSGLNLLSAALGFGMTAVFVAFVCARFVCCRARGADDSAPSPVDFDVDFPADLERPVEDANCGLEPLVIAAIPIMKYSEALYSKDDAQCSICLGEYTEKELLRIIPTCRHNFHRTCLDLWLQKQTTCPICRVSLKELPSGKAAIAPSCSNPQVRPRTENSVNPAPDWLLPVHHSHRGQQNSSDTQGSVEVVIEIRQ*

>TraesCS2D01G397200.1

MIFGSGLNLLSAALGFGMTAVFVAFVCARFICCRARGVDDGAPTPVDFDVDFPADLERPVEDANCGLEPLVIAAIPIMKYSEALYSKDDAQCSICLGEYTEKELLRIIPTCQHNFHRTCLDLWLQKQTTCPICRVSLKELPSGKAAIAPSCSNPQVRPRTENSVNPAPDWLPPVHHSHRGQQNSSDTQGSVEVVIEIRQ*

**B**


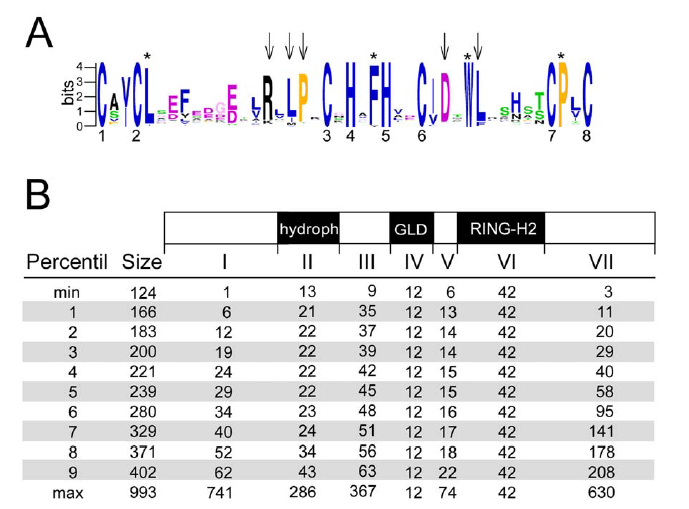


**C**


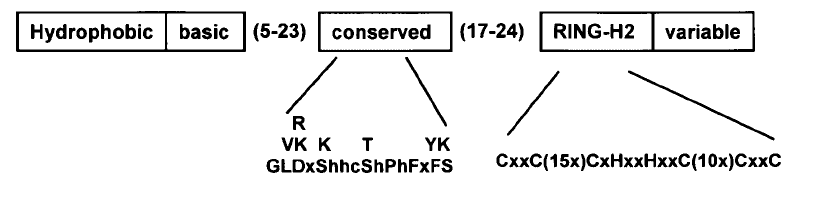


**Supplementary Figure S3.** **The sequences and conserved domains of proteins deduced from the cDNAs of the Y2H clones. A**, The deduced protein sequences in Chinese Spring. **B**, Predicted C3H2C3 domain using the program. The arrows and asterisks indicate conserved amino acids in the domain. **C**, The predicted domains in the ATL protein.

**
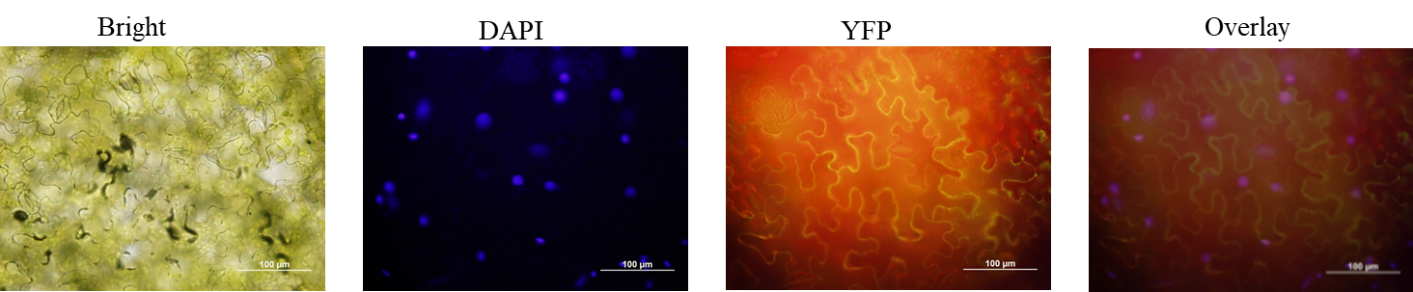
**

**Supplementary Figure S4. Localization of *Ta*E3V1 in *N. benthamiana* leaves.** **A**, A construct encoding a fusion between *Ta*E3V1 (TraesCS2B02G417700) and YFP was expressed in *N. benthamiana* leaves *via* Agrobacterium-mediated infiltration. Nuclei were stained with 4′, 6-diamidino-2-phenylindole (DAPI). Scale bars: 100 μm.

>*TaE3V-B1*-Jagger

ATGATCTTTGGGTCCGGGCTGAATCTCCTCAGCGCGGCGCTCGGCTTCGGCATGACTGCCGTCTTCGTCGCGTTCGTCTGCGCGCGGTTCGTCTGCTGCCGCGCCCGGGGCGCGGACGACAGCGCCCCATCGCCTGTGGACTTTGACGTTGACTTCCCGGCAGATCTCGAACGCCCGGTAGAGGATGCTAATTGTGGGTTGGAACCTTTGGTTATTGCTGCAATTCCTATTATGAAGTACTCTGAGGCTTTATATTCAAAGGATGATGCCCAGCGCTCCATATGTCTAGGTGAATACACCGAGAAAGAGCTTCTAAGAATCATTCCGACATGTCGACATAACTTTCACCGTACCTGCTTAGACTTATGGTTGCAGAAGCAGACTACTTGCCCAATATGCCGGGTCTCGTTGAAAGAGTTGCCTAGTGGCAAAGCTGCCATAGCACCTTCATGTAGCAACCCTCAAGTG..CCCCCGCACTGAGAACTCTGTTAATCCAGCACCTGACTGGCTCCTCCCTGTTCATCATTCTCACAGAGGTCAACAGAACAGTTCAGACACACAAGGATCAGTAGAAGTGGTTATTGAGATACGCCAATAA

>*TaE3V-B1*-2174

ATGATCTTTGGGTCCGGGCTGAATCTCCTCAGCGCGGCGCTCGGCTTCGGCATGACTGCCGTCTTCGTCGCGTTCGTCTGCGCGCGGTTCGTCTGCTGCCGCGCCCGGGGCGCGGACGACAGCGCCCCATCGCCTGTGGACTTTGACGTTGACTTCCCGGCAGATCTCGAACGCCCGGTAGAGGATGCTAATTGTGGGTTGGAACCTTTGGTTATTGCTGCAATTCCTATTATGAAGTACTCTGAGGCTTTATATTCAAAGGATGATGCCCAGTGCTCCATATGTCTAGGTGAATACACCGAGAAAGAGCTTCTAAGAATCATTCCGACATGTCGACATAACTTTCACCGTACCTGCTTAGACTTATGGTTGCAGAAGCAGACTACTTGCCCAATATGCCGGGTCTCGTTGAAAGAGTTGCCTAGTGGCAAAGCTGCCATAGCACCTTCATGTAGCAACCCTCAAGTGCGCCCCCGCACTGAGAACTCTGTTAATCCAGCACCTGACTGGCTCCTCCCTGTTCATCATTCTCACAGAGGTCAACAGAACAGTTCAGACACACAAGGATCAGTAGAAGTGGTTATTGAGATACGCCAATAA

>TraesCS2A02G399700

ATGTTCGGGTCGGGGATGAATCTCCTCAGCGCGGCGCTCGGCTTCGGCATGACCGCCGCCTTCGTCGCGTTCGTCTGCGCGCGGTTCATCTGCTGCCGCGCCCGGGACGCGGACGATGGCGCCCCATCGCCGGTGGACTTTGACGTTGACTTCCCAGCAGATCTCGAACGCCCGGTAGAGGATGCTAATTGTGGGTTGGAACCTTTGGTTATTGCTGCAATTCCTATTATGAAGTACTCTGAGGCTTTATATTCAAAGGATGATGCCCAGTGCTCCATATGTCTAGGTGAATACACTGAGAAAGAGCTTCTAAGAATCATTCCGACATGTCAACATAACTTTCACCGTACCTGCTTAGACTTATGGTTGCAGAAGCAGACTACTTGCCCAATATGCCGGGTCTCGTTGAAAGAGTTGCCTAGCGGCAAAGCTGCCATAGCACCTTCATGTAGCAATCCTCAAGTGCGCCCCCGCACTGAGAACTCTGTTAATCCAGCACCTGACTGGCTCCTCCCTGTTCATCATTCTCACAGAGGTCAACAGAATAGTTCAGACACACAAGCATCAGTAGAAGTGGTTATTGAGATACGCCAATAA

>TraesCS2B02G417700

ATGATCTTTGGGTCCGGGCTGAATCTCCTCAGCGCGGCGCTCGGCTTCGGCATGACTGCCGTCTTCGTCGCGTTCGTCTGCGCGCGGTTCGTCTGCTGCCGCGCCCGGGGCGCGGACGACAGCGCCCCATCGCCTGTGGACTTTGACGTTGACTTCCCGGCAGATCTCGAACGCCCGGTAGAGGATGCTAATTGTGGGTTGGAACCTTTGGTTATTGCTGCAATTCCTATTATGAAGTACTCTGAGGCTTTATATTCAAAGGATGATGCCCAGTGCTCCATATGTCTAGGTGAATACACCGAGAAAGAGCTTCTAAGAATCATTCCGACATGTCGACATAACTTTCACCGTACCTGCTTAGACTTATGGTTGCAGAAGCAGACTACTTGCCCAATATGCCGGGTCTCGTTGAAAGAGTTGCCTAGTGGCAAAGCTGCCATAGCACCTTCATGTAGCAACCCTCAAGTGCGCCCCCGCACTGAGAACTCTGTTAATCCAGCACCTGACTGGCTCCTCCCTGTTCATCATTCTCACAGAGGTCAACAGAACAGTTCAGACACACAAGGATCAGTAGAAGTGGTTATTGAGATACGCCAATAA

>TraesCS2D02G397200

ATGATCTTCGGGTCGGGGCTGAATCTCCTCAGCGCGGCGCTCGGTTTCGGCATGACCGCCGTCTTCGTCGCGTTCGTCTGCGCGCGGTTCATCTGCTGCCGCGCCCGGGGCGTGGACGACGGCGCCCCGACGCCGGTGGACTTTGACGTTGACTTCCCGGCAGATCTCGAACGCCCGGTAGAGGATGCTAATTGTGGGTTGGAACCTTTGGTTATTGCTGCAATTCCTATTATGAAGTACTCTGAGGCTTTATATTCGAAGGATGATGCCCAGTGCTCCATATGTCTAGGTGAATACACTGAGAAAGAGCTTTTAAGAATCATTCCGACATGTCAACATAACTTTCACCGTACCTGCTTAGACTTATGGTTGCAGAAGCAGACTACTTGCCCAATATGCCGGGTCTCGTTGAAAGAGTTGCCTAGCGGCAAAGCTGCCATAGCACCTTCATGTAGCAACCCTCAAGTGCGCCCCCGCACTGAGAACTCTGTTAATCCAGCACCTGACTGGCTCCCCCCTGTTCATCATTCTCACAGAGGTCAACAGAATAGTTCAGACACACAAGGATCAGTAGAAGTGGTTATTGAGATACGCCAATAA

>TaE3V-B1-2174

MIFGSGLNLLSAALGFGMTAVFVAFVCARFVCCRARGADDSAPSPVDFDVDFPADLERPVEDANCGLEPLVIAAIPIMKYSEALYSKDDAQCSICLGEYTEKELLRIIPTCRHNFHRTCLDLWLQKQTTCPICRVSLKELPSGKAAIAPSCSNPQVRPRTENSVNPAPDWLLPVHHSHRGQQNSSDTQGSVEVVIEIRQ*

>TaE3V-B1-Jagger

MIFGSGLNLLSAALGFGMTAVFVAFVCARFVCCRARGADDSAPSPVDFDVDFPADLERPVEDANCGLEPLVIAAIPIMKYSEALYSKDDAQRSICLGEYTEKELLRIIPTCRHNFHRTCLDLWLQKQTTCPICRVSLKELPSGKAAIAPSCSNPQVPPH*

>TraesCS2A01G399700.1

MFGSGMNLLSAALGFGMTAAFVAFVCARFICCRARDADDGAPSPVDFDVDFPADLERPVEDANCGLEPLVIAAIPIMKYSEALYSKDDAQCSICLGEYTEKELLRIIPTCQHNFHRTCLDLWLQKQTTCPICRVSLKELPSGKAAIAPSCSNPQVRPRTENSVNPAPDWLLPVHHSHRGQQNSSDTQASVEVVIEIRQ*

>TraesCS2B01G417700.1

MIFGSGLNLLSAALGFGMTAVFVAFVCARFVCCRARGADDSAPSPVDFDVDFPADLERPVEDANCGLEPLVIAAIPIMKYSEALYSKDDAQCSICLGEYTEKELLRIIPTCRHNFHRTCLDLWLQKQTTCPICRVSLKELPSGKAAIAPSCSNPQVRPRTENSVNPAPDWLLPVHHSHRGQQNSSDTQGSVEVVIEIRQ*

>TraesCS2D01G397200.1

MIFGSGLNLLSAALGFGMTAVFVAFVCARFICCRARGVDDGAPTPVDFDVDFPADLERPVEDANCGLEPLVIAAIPIMKYSEALYSKDDAQCSICLGEYTEKELLRIIPTCQHNFHRTCLDLWLQKQTTCPICRVSLKELPSGKAAIAPSCSNPQVRPRTENSVNPAPDWLPPVHHSHRGQQNSSDTQGSVEVVIEIRQ*

>RING/U-box superfamily protein, NP_192754 Arabidopsis

MSFIDPRTYIPSNSTESQILKFTFIVCVPICVILIVLLVLYIMRRNSNTNVDWSSLGGFVPTNNNLSTAELGLSKDIREMLPIVIYKESFTVNDTQCSVCLGDYQAEEKLQQMPSCGHTFHMECIDLWLTSHTTCPLCRLSLIPKPSVDLSHQSIEIVSSIENTNGGEASTQPDSQSATEAIIHIDDVEEGNRDSIEVVKESEENDRNSVGTSDGCCSCRLGEKA

**Supplementary Figure S5.** *TaE3V-B1* of Jagger and 2174*.* One SNP and a 2 bp deletion is the Jagger allele are highlighted in red. The start codon and the stop codon of the genes in cDNAs are highlighted in yellow and with red letters. The 43 a.a. encoded by the *TaE3V-B1b* allele and highlighted in grey lost in the protein by the *TaE3V-B1a* allele. The 3 a.a., highlighted in purple, gained in the protein by the *TaE3V-B1a* allele.

**C**

**A**

**
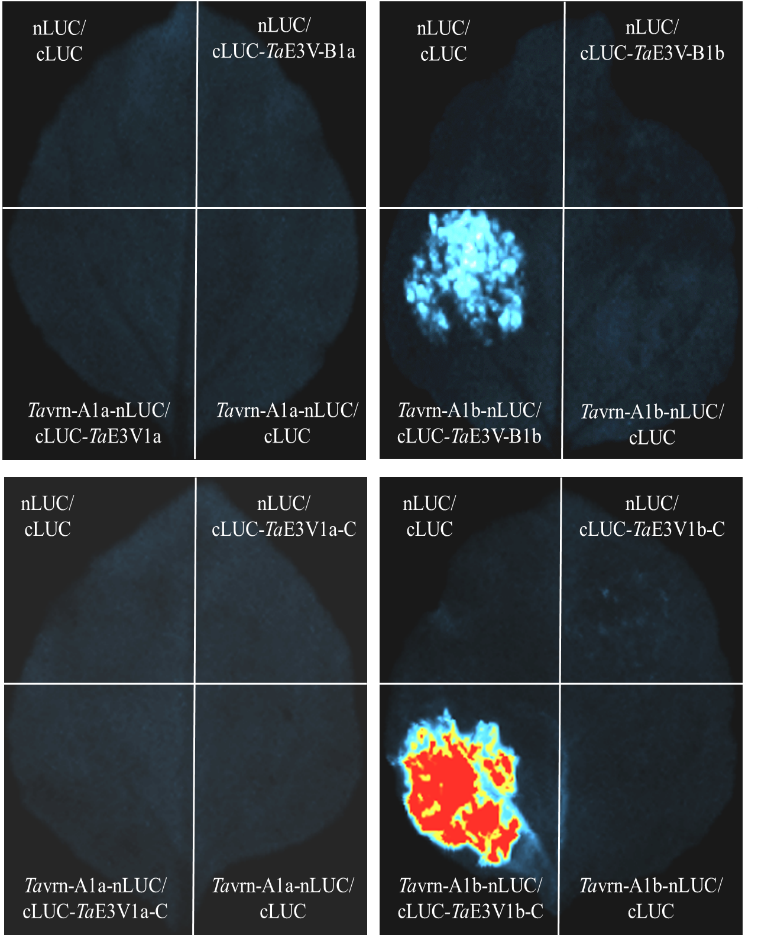
**

**
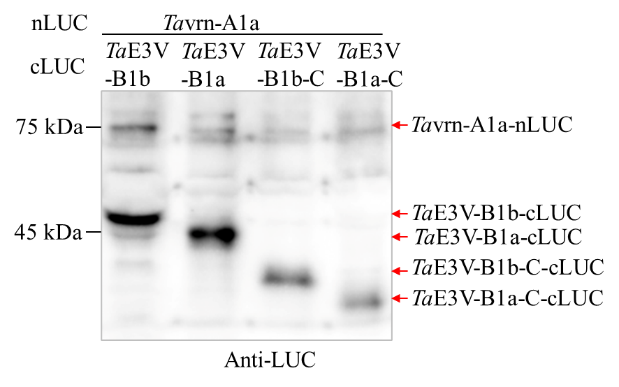
**

**
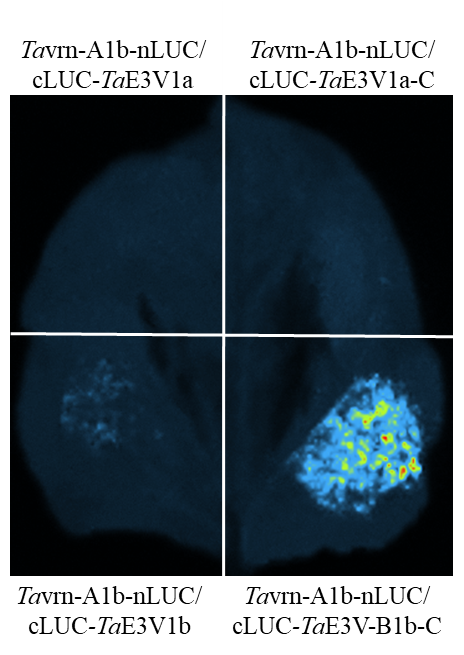
**

**B**

**Supplementary Figure S6. Interaction of *Ta*E3V-B1 and *Ta*vrn-A1b in an LCI assay. A,** Immunoblot detection of protein levels for the LCI assay. Immunoblotting assays were performed on nLUC and cLUC fusion proteins expressed in N. benthamiana, utilizing an anti-luciferase (LUC) antibody. The anti-LUC antibody (Abclonal, CAT# A18259) effectively detects recombinant fusion proteins that include the firefly luciferase sequences from amino acids 311 to 550, encompassing nLUC fusion proteins ranging from amino acids 2 to 416 and cLUC fusion proteins ranging from amino acids 398 to 550. **B**, Interaction of *Ta*E3V-B1 and *Ta*vrn-A1a in a luciferase complementation imaging (LCI) assay. The LCI assay was performed in *N. benthamiana* leaves. *Ta*E3V-B1b from 2174 and *Ta*E3V-B1a from 2174: the full-length protein with cLUC; *Ta*E3V-B1-C the truncated protein including the C terminus (from residue 78 to the C-terminus). nLUC, N-terminal portion of LUC; cLUC, C-terminal portion of LUC. The four combinations of *Ta*E3V-B1 and LUC were tested on the same leaf. **C,** Multiple negative controls for the interactions of *Ta*E3V-B1 and *Ta*VRN1. Since only four samples were tested on a leaf, the negative controls were tested on different leaves.

**
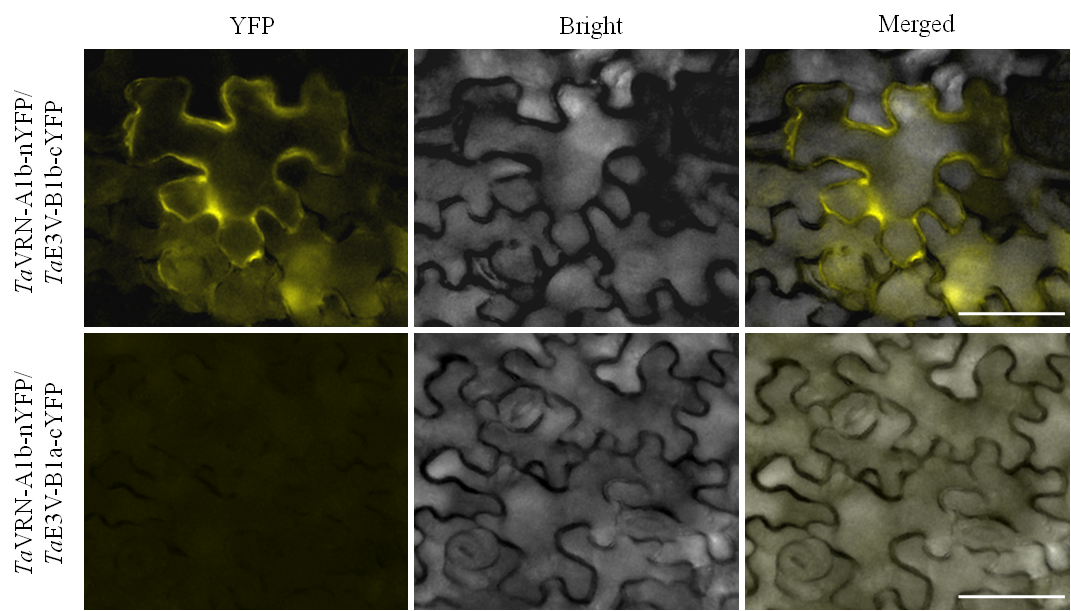
**

**A**

**B**

**
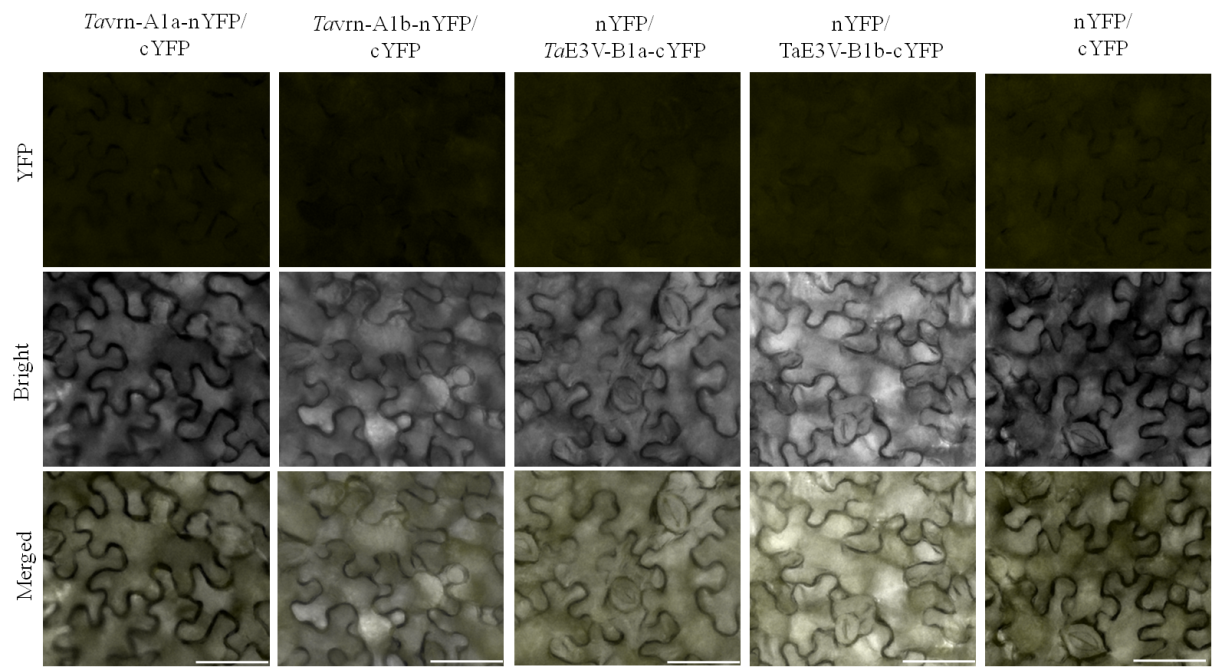
**

**Supplementary Figure S7. Interaction of *Ta*E3V-B1 and *Ta*vrn-A1b in a BiFC assay. A,** Interaction of *Ta*E3V-B1 and *Ta*vrn-A1b in a bimolecular fluorescence complementation (BiFC) assay. The BiFC assays were performed in epidermal cells of *N. benthamiana* leaves. The yellow color on the membranes indicates the signals of protein interactions. **B,** Multiple negative controls for the interactions of *Ta*E3V-B1 and *Ta*VRN1 in the BiFC assay. *Ta*E3V-B1a from Jagger; *Ta*E3V-B1b from 2174. nYFP, N-terminal portion of YFP; cYFP, C-terminal portion of YFP. Scale bars: 50 μm, applicable to all images.

>LY694-T7 sequence exported from chromatogram file

CGACAGCCGATGGTATCCCTACGACGTACCGATTACGCTCATATGGCCTGGAGGCCGTGAATTCCACCCAAGCAGTGGTATCAACGCAGAGTGGCCATTATGGCCGGGAAAGACTATCACCCTTGAGGTTGAGAGCTCTGACACCATCGACAATGTCAAGGCCAAGATCCAGGACAAGGAGGGCATTCCTCCAGACCAGCAGCGTCTCATCTTCGCTGGGAAGCAGCTGGAGGATGGCCGTACCCTCGCTGACTACAACATCCAGAAGGAGTCCACCCTCCACCTTGTCCTGCGTCTGCGTGGTGGCATGCAGATCTTCGTGAAGACCCTCACTGGAAAGACTATCACCCTTGAGGTTGAGAGCTCTGACACCATCGACAATGTCAAGGCCAAGATCCAAGACAAGGAGGGCATTCCCCCGGACCAGCAGCGCCTGATCTTTGCTGGCAAGCAGCTGGAGGATGGTCGCACCCTCGCCGACTACAACATCCAGAAGGAGTCCACCCTTCACCTGGTGCTCCGCCTCCGTGGTGGTCAGTAATTGCCCTCTCACTCGACCTGCTGCTGCTGCTGTACCCTGTGTCGTCCTGTGGGTGCGCCTTTGTTCAAGTGCTGTCTCGTTTGTGTGAAGTCGTACTGTGTCTGGTTTAATGGACCATCGAGTCCTGTGGTTTGGTGCAAACAGTTGCAGGTACATCTGCACCGTTAAGTATGAATAAGTCAACTTTGAAGTAATGTTCCTGTGCAAAAAAAAAAAAAAAAAAAAAAAAAA

>LY696-F1-D-R sequence exported from chromatogram file

TGACGCTCGGAGGCACAAACTGAGTGCGTTGAAGTAGACGGCGGCGACAGGCGGGCCGAGGTTGTAGAGCTCGGCGAAGTCCCTGGTGTTGAAGTTCTGGCGCCACCCGGGAGCGTACACGGTCTGCCGGCCGAGCTGCTGGAAGAGCACGAGCACGAAGCGATGGATCCCCATGGTCGGACGAGGGCTCTCGTAGCACATCACCTCCTGCCCGAAGGATGCACCAGTTGTACCGGGGATATCTGTCACAAGCCAGTGGAGATACTCCCTAAGGTTGGGATCGCTTGGACTTGGAGCATCTGGGTCTACCATCACGAGTGTGTAGAAGGTCCTCATCTCATTGCCGCCCACCTCAACCCTGGGCTGCTGGGCGACCATGGACGGCTTGAGCTCGCAGCCGTTGGACACGGTCCTGTTCCCGAAGGTCACCCTGAGGTTGGTGGTCCAGATGAATAATCCCGCGGCCATGGCGGCCGGGAGCATGCGACGTCGGGCCCAATTCGCCCTATAGTGAGTCGTATTACAATTCACTGGCCGTCGTTTTACAACGTCGTGACTGGGAAAACCCTGGCGTTACCCAACTTAATCGCCTTGCAGCACATCCCCCTTTCGCCAGCTGGCGTAATAGCGAAGAGGCCCGCACCGATCGCCCTTCCCAACAGTTGCGCAGCCTGAATGGCGAATGGACGCGCCCTGTAGCGGCGCATTAAGCGCGGCGGGTGTGGTGGTTACGCGCAGCGTGACCGCTACACTTGCCAGCGCCCTAGCGCCCGCTCCTTTCGCTTTCTTCCCTTCCTTTCTCGCCACGTTCGCCGGCTTTCCCCGTCAAGCTCTAAATCGGGGGCTCCCTTTAGGGTTCCGATTTAGTGCTTTACGGCACCTCGACCCCAAAAAACTTGATTAGGGTGATGGTTCACGTAGTGGGCCATCGCCCTGATAGACGTTTTTCGCCCTTTGACGTTGGAGTCCACGTTCTTTAATAGTGGACTCTTGTTCCAAACTGGAACAACACTCAACCCTATCTCGGTCTATTCTTTGATTAATAAGGGATTTTGCCGATTTCGCCTAATGGGTTAAAAAATGAGCTGATTTAACAAAAATTAAACGCGAAATTCGGCCTCCATGGGCCATATGCAGGTCCTCCTCTGAGATCAGCCTTTCTGCTCCCTCCATGATTGCGGCCTCGCCCTATATTGGGGTTCGTAATACACGAATTTCGCGAATACAGTCCAACTTCATGAACCCTTGGTACTACTCCTCTGCACGATGATATGAGTTCACGCATCACTATATTCCTGA

>LY705-T7 sequence exported from chromatogram file

GGGACAGCGATGGAATCCCTACGACGTACCGATTACGCTCATATGGCCTGGAGGCCAGTGAATTCCACCCAAGCAGTGGTATCAACGCAGAGTGGCCATTATGGCCGGGGACCCTGACCGGCAAGACCATCACCCTGGAGGTCGAGTCCTCTGACACCATCGACAACGTCAAGGCCAAGATCCAGGACAAGGAGGGCATCCCACCGGACCAGCAGCGTCTCATCTTTGCTGGCAAGCAGCTTGAGGATGGCCGCACTTTGGCGGACTACAACATCCAGAAGGAGTCCACACTTCACTTGGTGCTCCGTCTGCGCGGTGGCCAGTAAGCTCCTGGCCATGGATCTGCTTCTGTCTCTGGGTTCACAAGTCTCGTTGTCCTCGGTGTCCTCCAATGGAGTCTGGTCTGTGTCCGTTGATGCCTGAACTGTCTTTGTTTGTATACCATATACTGTGATGCAGTGTTATCGTTTGTATCTGCAAACTTCTGCTGGTGTGCGGAAGCTTTGATGAACTTAAGAATAAGTGAGCCCTGGAGTCGGT

CCATAATTCTGTTTAAAAAAAAAAAAAAAAAAAAAAAAAAAAAA

>LY708-T7 sequence exported from chromatogram file

AGCGACAGCCGATGGAGTCCCTACGACGTACCGATTACGCTCATATGGCCTGGAGGCCGTGAATTCCACCCAAGCAGTGGTATCAACGCAGAGTGGCCATTATGGCCGGGGACCCTGACCGGCAAGACCATCACCCTGGAGGTCGAGTCCTCTGACACCATCGACAACGTCAAGGCCAAGATCCAGGACAAGGAGGGCATCCCACCGGACCAGCAGCGTCTCATCTTTGCTGGCAAGCAGCTTGAGGATGGCCGCACTTTGGCGGACTACAACATCCAGAAGGAGTCCACACTTCACTTGGTGCTCCGTCTGCGCGGTGGCCAGTAAGCTCCTGGCCATGGATCTGCTTCTGTCTCTGGGTTCACAAGTCTCGTTGTCCTCGGTGTCCTCCAATGGAGTCTGGTCTGTGTCCGTTGATGCCTGAACTGTCTTTGTTTGTATACCATATACTGTGATGCAGTGTTATCGTTTGTATCTGCAAACTTCTGCTGGTGTGCGGAAGCTTTGATGAACTTAAGAATAAGTGAGCCCTGGAGTCGGTCCATAATTCTGTTTAAAAAAAAAAAAAAAAAAAAAAAAAAAAAA

>LY712-T7 sequence exported from chromatogram file

TCGCAGCCCGAATGGATCCCTACGACGTACCGATTACGCTCATATGGCCTGGAGGCCAGTGAATTCCACCCAAGCAGTGGTATCAACGCAGAGTGGCCATTATGGCCGGGGGGCAAGACCATCACCCTTGAGGTGGAGTCGTCTGACACCATCGACAATGTCAAGGCCAAGATCCAGGACAAGGAGGGCATTCCTCCAGACCAGCAGCGTCTCATCTTCGCTGGGAAGCAGCTGGAGGATGGCCGTACCCTCGCTGACTACAACATCCAGAAGGAGTCCACCCTCCACCTTGTCCTGCGTCTGCGTGGTGGCATGCAGATCTTCGTGAAGACCCTCACTGGAAAGACTATCACCCTTGAGGTTGAGAGCTCTGACACCATCGACAATGTCAAGGCCAAGATCCAAGACAAGGAGGGCATTCCCCCGGACCAGCAGCGCCTGATCTTTGCTGGCAAGCAGCTGGAGGATGGTCGCACCCTCGCCGACTACAACATCCAGAAGGAGTCCACCCTTCACCTGGTGCTCCGCCTCCGTGGTGGTCAGTAATTGCCCTCTCACTCGACCTGCTGCTGCTGTACCCTGTGTCGTCCTGTGGGTGCGCCTTTGTTCAAGTGCTGTCTCGTTTGTGTGAAGTCGTGCTGTGTCTGGTTTAATGGACCATCGAGTCCTGTGGTTTGGTGCAAACAGTTGCAGGTACATCTGCACCGTTAAGTATGAATAAGTCAACTTTGAAGTAATGTTCCTGTGCTCATTTGTCTTTACAGTTTCTGGGCTGTAGGTCCTAGTTTTTGGATTTTGAATGAATATTGATGCGAACTCGTTAGGTGAAATGCTAGATTTGGATGCTGCTGCGGCTCTAGATGCAAGTTATATTCAAATTTCGTTACTTGTTAAAAAAAAGAAAAAAAAAAAAAAAAAAAAAAAAA

>LY732-T7 sequence exported from chromatogram file

AACTTGGCCGCGTCGCATGCTCCCGGCCGCCTGGCCGCGGGATTGGATCCTGGGGCGGGGGAAGGTGCAGCTGAAGCGGATCGAGAACAAGATCAACCGGCAGGTGACCTTCTCCAAGCGCCGCTCGGGGCTTCTCAAGAAGGCGCACGAGATCTCCGTGCTCTGCGACGCCGAGGTCGGCCTCATCATCTTCTCCACCAAGGGAAAGCTCTACGAGTTCTCCACCGAGTCATGTATGGACAAAATTCTTGAACGGTATGAGCGCTATTCTTATGCAGAAAAGGTTCTCGTTTCAAGTGAATCTGAAATTCAGGGAAACTGGTGTCACGAATATAGGAAACTGAAGGCGAAGGTTGAGACAATACAGAAATGTCAAAAGCATCTCATGGGAGAGGATTTTGAATCTTTGAATCTCAAGGAGTTGCAGCAACTGGAGCAGCAGCTGGAAAGCTCACTGAAACATATCAGATCCAGGAAGAACCAACTTATGCACGAATCCATTTCTGAGCTTCAGAAGAAGGAGAGGTCACTGCAGGAGGAGAATAAAGTTCTCCAGAAGGAACTCGTGGAGAAGCAGAAGGCCCATGTGGCGCAGCAAGATCAAACTCAGCCTCAAACCAGCTCTTCATCTTCTTCCTTCATGCTGAGGGATGCTCCCCCTGCCGCAAATACCAGCATTCATCCAGCGGCAACAGGCGAGAGGGCAGAGGATGCGGCAGTGCAGCCGCAGGCCCCACCCCGGACGGGGCTTCCACCGTGGATGGTGAGCCACATCAACGGGTGCGGATCCAATCACTAGTGCGGCCGCCTGCAGGTCGACCATATGGGAGAGCTCCCAACGCGTTGGATGCATAGCTTGAGTATTCTATAGTGTCACCTAAATAGCTTGGCGTAATCATGGTCATAGCTGTTTCCTGTGTGAAATTGTTATCCGCTCACAATTCCACACAACATACGAGCCGGAAGCATAAAGTGTAAAGCCTGGGGTGCCTAATGAGTGAGCTAACTCACATTAATTGCGTTGCGCTCACTGCCCGCTTTCCAGTCGGGAAACCTGTCGTGCCAGCTGCATTAATGAATCGGCCAACGCGCGGGGGAAGCGTTTGCGTATTGGGCGCTCTTCCGCTTCCTCGCTCCTGACTCGCTGCGCTCGTCGTTCGCTGCGGCGAGCGTATCAGCCTCACTCAAGCGTAAACGTTATCCAAGATCAGGATACGCCAGAAAGACCTGTGACCAAGCAGCAAGCAGAACCGTTAAGGCCCGTGCTGCTTCTAGTCGACGTGCAACTCAAATCGCGCGCAGAGCCGCTCATGATTCG

>LY733-T7 sequence exported from chromatogram file

CAAACCAATGGGCCCGAGTTCGCTGCTCCCGGCCGCCTGGCCGCGGGTTTGGATCCTGCGGACGATCTGCGATGTGTGCGAGGGCGCGCCGGCGGTGCTCTTCTGCGCGGCCGACGAGGCCGCGCTCTGCCGGGCCTGCGACGAGAAGGTACACATGTGTAACAAGCTTGCTAGTCGGCATGTGAGAGTTGGACTTGCAAACTCTAATAAACTTGCCCGCTGTGATATATGTGAAATTTTTCCTGCTTTCTTCCACTGTGAGATAGATGGCACCTCACTTTGCCTGAGCTGTGACATGACTGTTCATGTTGGTGGCAAACGAACCCATGGAAGATACCTGCTCCTAAGGCAAAGTGTTGAATTTCCAGGAGATAAACTAGGCCATATGGATGATGATGTGGCTATGCAAAGCAAAGATCCTGAAAACCAGATAGATCAGAAGAAGCCTCCTCATTCAGCAATAAAGGAGCAAATGGCAAACCACCATAGTAGCTCTGATGATCCAGCATCTGATGGAAACTGCGATGACCAGTTGAACCTTAATTCGAAAATGATCGACCTTAATATGCGACCGGTTCGTACCAATGGACAAGGATCAAATTCCCAGACTCAGGGTGTGGATGTTAGCATGAACAACCATGACTCTCCTGGGGTGGTGCCGACAAGTAATTACGAAGGAGATGCCAACAAGTACGGATCCAATCACTAGTGCGGCCGCCTGCAGGTCGACCATATGGGAGAGCTCCCAACGCGTTGGATGCATAGCTTGAGTATTCTATAGTGTCACCCTAAATAGCTTGGCGTAATCATGGTCATAGCTGTTTCCTGTGTGAAATTGTTATCCGCTCACAATTCCACACACATACGAGCCGGAAGCATAAAAGTGTAAAGCCTGGGGTGCCTAATGAGTGAGCTAACTCACATTAATTGCGTTGCGCTCACTGCCCGCTTTCCATTCGGGAAACCTGCCGTGCCAGCTGCATTAATGAATCGGGCCAACGCGCGGGGAGAGGCCGGTTGCTATTGGGAGCTCTTCGGTTTCCTCGCTACCTAATCCTTCGCTGGGTCGTTCGGCTGCGGGAAGCGGTATCAGCTTACTCAAAGGGGGTAATCGGTATTCCACTAATTAGGGGATTACCTAAGAAAAAACATTGTGACAAAAGGCAACTAAGAGCCGGTACCCTTAAAAGGCCGCGTTGTTGGTCGTTTTTCCATTAGGCTCCCGCCCCCCGTGGCAAGATCTAACAAAAATTCGACCCTCCTAAGTCAAAAGGGTGGGAGAAACCCCGACCGG

**Supplementary Figure S8.** **The cDNA sequences of seven clones in the second set from the Y2H library using VRN1 as a probe.** The sequences, highlighted in yellow, are poly(A) in the cDNA seqeunces. Four clones, LY694 (641 bp), LY696 (641 bp), LY712 (893 bp), and LY732 (756 bp), have the same gene with different lengths.

>TraesCS6A01G136600

ATGTCAAGGCCAAGATCCAGGACAAGGAGGGCATTCCCCCGGACCAGCAGCGCCTGATCTTTGCCGGCAAGCAGCTGGAGGATGGCCGCACCCTTGCGGACTACAACATCCAGAAGGAGTCCACCCTCCACCTGGTGCTCCGCCTCCGTGGTGGTCAGTAATTGTCCTGGCGTTGTACCTGCTGGTTTATCCTTGGTCGTCCGGTTGGTGGTGCCCTGTCGATCGTTTGAACTGTTTTCTCGTTTGTGTCAGTCCTATGTGTTGGTTTGAAAACTATCGAGTCCCCATGTTATGTTGGTTGAAACCGTTGCTGGCACAGCAGTATCCTTAAGTAATGAACTAGCAAAAGGGCCCGTGCGTTGCACCGGGAGAACAAAATCACATGCCCCTAACCCAATCAGAATCAATCTTAGGAATAGTCATGTAATAAGAGTCAATCTGTACACCCACCACAGCTTCTGATCATGGCGTTTTAAACATAGTTGTACACTTAATTCAAAACCCGCAACAACTACCAGGAAGGCAACCAGCTCGTATGAGGTCTTCCACTGTGTTACAGGTGCCAACTTGTCTAAAAGGTTGTCACATCACCACCGATGCCAAATGAAAAAGTTATTACACCGGCTTCACATCATAGGGCACCGCCCAGTCTGAAAGAAGGAACCCAGGTTTAGATAAAACGCTATATAAAATCATCACTGGAGCATCTCGAGCGAGTTCTTCGTCCGCCGAAAATTCCCTTTGTTCTCCAAATCAATTCTTTCTATGGCTGAAACCACCACCCCGTCATATTTTTATGTTTACTTTAGCACACATAGTTTAGTAGTACAACATAGAGAAAAACATAAATGTATTTAGAACTGAAAGTAAATATAAGAAGCATGACCATATTGTTGGAGATTATTAATCCAAGTCCTATAGAAAATAGAAGTGTTGAAGCAGTCTGTACATAAAGTTGCAAACCCATCCGGATGAAGTAGTCTACAAGTAGTTACGGATTGTAACGAGCTGACGTTAAAACTAAAACCAGAGCAGACACACAATTTCTCTCTCAAAAGAAAAAAATATCATTATTATAGCCGACTACATGAATTTATAAAAAGAGAAGTGAAAAGAAAATCAAGTTACAGGTGAATAAATTACAACTACAGATTTAACAATACCTGCACTGCGTGTAAGGTCAAATTGATTCAATTGATACTAACATTCAGCATTTCTTATGAAAATGTCTAACGCTTATAGCCCGAAAGCTGAAAAATTCTTTGCAAATTGTTTTATTTTTTGGAGCTTATACATGTATGGGTGGGTGCCTCAGTTCAGGATTTAACTAAGTAAACATCTTAATGTTGAAACTTTTGGTGAAATCTTATCACAAAGAGGAATACTTATGCAAATTTCATCAGTGTAGAGTGCCTTATTGCTGGCCTTTCCACCATGATTGCAAGAATCAATTTGCCACCAAAAAGAAACCCCAGAGACATGTGTATTTAGACGATGCTAATGTGCACTGTACAATGGCAGAATAAAAATCATCTCAAAATTAGGTTCAACAATACATTAATTAGGTTTCTTTTAGGAGCAATTACCTACTCACGTCCATGTTCACAGTAGTGGAAAAAAGGAATATAATTTCTACTAAAGCAAACAACTATGCTTCCTGTACTAACCTTGGGCATTGCTCGAATGTTTTAGCGGCTGCTTGTGTCATTCTTAGTTGCTAAATACAACCAAAAGTGTAGTATTTAGAAGCAACGGCGTAACTTGCAACAAAATAACTATTTGTATAGTAAATGGCCTAGTTCACTAATCAGGTAGAAACTGTTCAGGCAGGTTGTCCTTGAAGTTCTCCATGTCGTGAGCATAACTTCATTGGCTGAAAAAACTGCTGATACACTTAAAATTAACAACCAACACTATGCTAGTATAGTATGAAAAAACAGTAAGTCTATCTGTTTCACACCAATGTTTCTTCCAACGAAGGATCACCCCCATTGCTATACCAAACTGAGGGCCTGAGGCTATTTCCCTTTGCTATACAGAATAACAAAAAAGGCTCTACACCCAAGCATCACACCACACAATCTTCCAAGGATGCTATACAGAATAAAGCACTACCTTACGAACAATTTCAGATGAGGACACTGACCAACAAATATTGCAGTTAAAATTTTCAAGTTGTGATCTCTGGTGCTCCCCGGCTTGGTTGTGATAGTCGTGGAGCACAGCCAGATCCTAGTCCTCCTGTGGACGCGGACGCGCAGATCCGACCATAGGCGAAAGAGGCCATAAACCAAGAGAAACAAATCAATAGTGGCCTAGACCATGCGAGCCCAGCGTCCTCGATCCCAGGAGCCATGGCCTTAGTGGCGACATGGAGATCATGGTCGATGCCCAGCAGAGGAACACAGCTAGCAAAGAAGGATAAATAGGAACCTATTTCTCACCTTGGAGGTGTTGCTGCTGCTGCACTGAATTGTTTGAACCTCGCTTTCTGTACCTCCTTGGAAACCTGAAATATGCAAATGTTAGAAAGCTTATAATTTGTATATGATTATTTTAAGAGAAGGGGAAAAGAGTATCAAGAATAGATTCATACTTTTGGCTGTTAGCCATTACAGGCTTGAATATATCTATTTCTGTATGCATGTGCAAACTATCATAAATCAATAATAGTATTATCGTTTAGCTATTCCATGGAACAATTTCTCTGAACATTCTTGCTTCAAAGTTGTCAGAAGTTCTAGCTATGACAAAGACATGTACTAAAAGTTCTCACAAATAACCAAAAATAAAAAGGCATATCAAGACAGGAAGATGTTTGTTTGACCAAATAGTTACCACTCTCTGAACCATATTGCCAAAAATTAGCAGGAACATGGTCGTGAACTCATCCCCAGTGATCTCTCTTTAAACCAAACACTCCCTGACATATGGTCATCCCACTCACCTTCCTGCCACTGAAGAAAACAATCTAGATGCAGAACGTCGCGAGAGGTGGGGAATAGCAGGAGCTTGCGAGGGAACCACTCCAAATCGGCCTTGTACACTTTGAACGCGGAGCATCCATGGTTGCTCCTGTAAACGAAGGACGGCGGTGAGATGATAAGAGCTCCGAGAGAGAGAGAGAGAGAGAGAGAGAGAGAGAGAGAGAGAGAGAGAGAGAGAGAGGAGCAAGGGGGGAAGGGGAGAGACACAGGAGACCTGGGCGGCTCCGGCGCGACGGCGCGTGCTGTCCTCCCGCGGCCACGCAGCAGTGGTCGTCGGAACCAGCCCCCGCACGGGATCCCTCCGTCTCCCGCACCCGTCCGCCATCGCCCGGCTCCGGCGAGCTCCGGCCTTCCCTCCGTCTCCCGCGCCCGTCCGCCATCGCCCGGCTCCGGCGAGCTCCGGCCTTCTTCTCTCCGCACCGCTTTTCCTTTGTCCCTTCTTCTCTCCCTCGCCTCCTTGTCTCCCCTCTGCCCAATAGTCGTGGATGCCATGAACCCGCCCGGGAAGCCGCGCTCCGGCCTTCCTCGTCGTCACCGGCGATCCTCGCCACGCCGCCCGCCTCCGGATCCGCCTCCCCAGCCCCTACCGCGCACGGATCCGGCCCCTGCAAGCTCCAGCCGCTGCCGCCGTTCTGCATCGCGCTGCCGTGCCACCGCGTTGACCCGACCCTAATTAATAACGAATCGTTTTTCACTTATCCATGGACTGCGGGTCAAATACTAAAAAGCACACGGACCTTTATGCAAAAACGCCGACGACGTACGGGCAGAAGCACTCCGTGCTTTATTAGTAGGAAAAGATAAGTGAACTTCAGATAAATCTGCTTGCGCTAATTTGCTGTTGGTTCTGTATTGTGCTTGAATTGTTTGCTTGCAGTTTTGATCTGGCAATGATCTTATTGCGGCCTTTCTGTTTATAGATTGTGCTTGAATTGCTTGCTTGATATATTGTGGCTTTCTGTGACTTTCTGGACTATAGATGCTAATTTCCTAGGTTCTGAATGTTCCTGTGAAACCGGTGGCTGCATTGTTCAGATTTGGATGATGCAATTTTATACTTCTATCTTGTTTCTTGAAATCCTTTGTGGGATCCCTGCTGCGTTATTGCGTGGTCGGTTTGTGTTAAAATTCTTGTTCCAGTTTTTTTTTTTTGCGTGTGTAGTCTGCATGACGGCAGGTGAACACAAATATTTCTAAGTTAGATGCATTGTCGTTGATTAGCGGCGCTAACTCATTTGCTGGGTGCCTCTCATTCTTCCCCTAGCTCCTGCTCCACCGCTGTCGAAATATGTTGCCTTGGGGCCGTGGCGCACCAAGGATTTTTGCTGGAATCGGAGGCGATGGATGTTACAGTCGGTGGTTAAGGAATGCTACAACCCATGGTGAGGGAGGCTACAACCGGTGGGTGTTGGTTCTGGAGCGGAGGAGTTGGGGCGACCGCCGCTGGGTGCTGCAACCGGTGATGACAGGGTGATTTTTTTTTAGCGGCGGAGGTTGTTGTGAGGGCAGCGAGGCCGGTGAACCGGGTGGTGTTGTCAACTTGGTTGGTTATGGTGGAAGTTGGCGGTGCTTGTGCCCGGAGCAATTTTTGAGGGAAAGAAGAAGGGCTCAATGTCAGGAGGAGGACGAAGCGAATAGGAGGATGATTCGAAACGGGTCGTTGCGCGCCGCTTAGCACTGTCGTTCATAACATAGCTTGGGGATACTTGGATACTTTTTTTAATGGAAAGCCCTGCACTTTGTTGCATTGCATCGTTCAAAATGGATGCAATATGGATACACCTTGGCAAAACAAGTGCACAAATTCTTGACCCGGCACCATAATCATTATGCGCCTATACTTGTTTAACATGGGAATTATGAGTGGGCATATAGAATGCTTTGGCTTTTTTTGACCGAATCCAAGGTAGCGCCGAGCTGAAATTATAAAACAGCAACACGTCTCATTCACATCGGTTTAGTCTCACATATTTTTTTTTTAAATCCGGCAACGGTCGGCGTTTCATTGATTAAGCAAAATATTATTGTTGCATTGGTCTATTATGCCAACAGATGGCAATCACTAGCCGCCAGTTGCTAATTTTGCACGATATAGTTAGTTGAGTACATTTGCAGCTTCCTGCCGCAATCAAAGTGATGTCGTGTGCATATATGTCCTGCTCTACAAAATACTCTTATCTGTTCCTTAATAAACAGAAAGTTCAATCAAACAATTCACACATGCCCTGGTCCTGCCAATTTTGTATTTCTTGTGAGAGATACGAAAAATATAGTAATATACTGAACTTTGGAGAGATAGGAGTAATACAATCGTAATATGATGTCCAGCGGTTTTCGGGAGAGCAAAAACCTAAAATGTAAAAGTGCAATTTTAGGAAGTACTCCCAACACTTGTCCCTTATCCGTGCCTATAAAAGAGAAAGCAACAAGAGAAAACAAAGGAGGTTTTGGCAACGGGGACATGCATCATTCCTCATTTTCTCCATGTAAGGATGCAAGTAAACCAACAAACTAACCTGTTAAATGTGCAAGTTATGGTTGAGTCCTAACCATTGAGTTGTTTGATTGTTGCGGTACATTTTAACCTAACCTCCCGCATTTTGTGGGATTGAGCGGACCCGCTCTTGCATCCCTACCTCTATGCCTATAGCTCAAACGAGTCCATATGACAAATACTCCCTCCGTAAACTAATATAAGAGTGTTTAGATCACTAAAATAGTGATCTAAACACTCTTATATTAGTTTACAGAGGGAGTATCAGATAAGAACAACCAAGAAATCATTAGAACTCTCTCAGTTCACTATTATAAGATGTTTAGGATACTTTAATATAGACTATATACATACCAAGATGACTGAACAAACATTCTAAAACTTGTCTACATACATCGATTTAGAAAAAAAGTTCGAGTGTCCTATAATAATGAACGGAGGGAGTATTATTTTGTGCAGGTATGCCAAGTTGCCAGCTTGCATTATTCGTTACTTACATGTGGTCCTGTATAAACTCATTCATCCCAGCTAATCGTCCATGAATGTATGTAAAAAATGATCCATATAATATATTTGGAAAGTACCCATTCGTTAAGTATGAATTCACCTGCTCTAGAGTATGCCAAGTGTTTTTTTTTGCAAATAAAAAAATGTGTTCCTTTGCCGTCACAAACTATGCAAAAGTATGAACCCATCAAGTCAAAAGTAGCTCGTTGCGAATCAACCTGTGGTTGAGATGGTTAGATGGACAGTGGTATCCCAAACCCACCAGGATTCAAATCCTGGTGCTCGCATCATTCCTGAATTTATTTCAGGATTTCCGGCGATGCGTTTCAGTGGGAGGAGACGTTCCCGTCGACGACGAGGCGCCTACGGTGACTTCGTAAATCTCAAGATGATATGCCGGCTCAGTCTCTCGGAGGTGCTCATAGGGGTAGGATGTGCGTGTGTGCGTTCATAGGGATGAGTGTATGCGCGTGTATATGAGCGCTTGTGTCTGTACTGATGCTAAAAAAAAGTCAAAAGTAGCTCACTGGAAATTTATTCTTTTCTATACTAGGAGTGGAACAAAATGACACGTTAATTAACATAGCATTTTTTAGTAGAGTTAATTAACATGGCATTTTTTTTTGTAATACTCAACTTACGATCATCACACCTGCACAGACCAGCCCAGCGGGCCATACTTACCAGCGACACGGACAAAACGTCCATCTAACGGCGTGATCGGGTGGCTAACGGCCATCAACCCCGCCCGGGGTAGACGGCCGTCCTCTCTCGAATCCTCCGCGCCCGTCGGATCGTCACGGCCCGGGACTTTTCCCCCACCCGGCCCGTCACCAGCCGCCATAAATACCCGCCCCCTTCCTCGCCTCAGGATTGTAAACTCGCCACCGCTCCCAGGAACGGAACCAACAAATCCCCACCAATCTCGTCTCCAATCGACCCGAGGCTCGTCGAATCCTCCTTCCCCACCGCCTCAAGGTACCGCGCTTGCTCCCCCTCTCCCTGTATCGTCTGGATCTACGTTTCTATGGTGCTCCGTGATAGCTGGACGTCTAGTGCTGTCGAATTTGGCTCCGATCTGTGGTTAGATCGGTGAAGATTTAGCGAATTCGTATGGATTTCGCGTGTGCTAATTTTTTATCAGGATGAATTCCTTGATGACTTGTGGTCTCGTATGTTAATCTATGCATGTTTATGTTCTAGTGCGTCTTCCTGCTTGATAGATTCCCCCAAGTAGCTTGTGTGCCATATAAAATTCTCTCCAGACGCGTGTACTGCCTGATCTGCAGGCCTAAGATTGTGGATTGGTTTTGACCCGAAGATAGGTTTCGTTCTGATAGCACGCCCACCAGCTATATGGAAATCCGTGAGAAAATTGATAGTCTGGATGTGCGTGAACCTGTTTGCTCGCTGCTATATCTCTGATGAAGAAAATATGTCTATTGAGTCTCTGACTTATCTATTTGCGTTGTTAGATTGGTTATAAGCTTTAGCGAATTTGTACGGATTTTGTGTTTATTTTGTAATTTACCAAAAAAGGCTTTCGCCCCGCTTTATATTATAAAGCAACTGCCCAAGCCAAACATCCAACAAGGTTCAAACATACAAACACACGAAGGTCTAGCACAGACAGGTAATCAAAGGGTTAATGCTGAGGGCACAGCTCAACAAGCCCTGAAAAAATTGACAGAAGGGTTAGGTCTATCTGTCTTCCGGGTCAATTCCTGATGCCATATGGTCTCATATGTTAGTCTATGCATGTTTTATCTATTATTACAGTTCCGTTCTGGCTTGGTAAATTTTTTATGATCCTAAATAGTTGCTGCGCGATAAATTTTTCTCCACACACAGATACTGCCTGATCTGTCGCTCTTTATCAGATTGCTGTTGTACCAAGATTGTCCTAGTAGGCCTAGGATTGCAGATTGTTATTTTTCCTGAAGCTTCTCTAATCTTTGAGATAGCGAATACATGTAGATTAGTGTCAGATGTGCTTGATTTATTACCCTGAAGCTAGGTTTCTCTTAGATAATAAGCATGTCTGGCAGCTATGTTAAATCATTAGATCAATGAAATCTGTAAACATTTGTAGAAGCTTGTCCATGCATTTGTGTTTGTTCTTCCATCATTGTGAGCTTGTCCATGCATTAGTATTTGTTGTTCCATCATTGTGAGTTTGCCTGGTCTGCTTCTTTATTCTTACCATCCTATCTGCGATTATTCTTTGCAGATGCAGATCTTTGTGAAGACACTGACGGGCAAGACCATCACCCTTGAGGTGGAGTCGTCTGACACCATCGACAATGTCAAGGCCAAGATCCAGGACAAGGAGGGCATTCCTCCAGACCAGCAGCGTCTCATCTTCGCTGGGAAGCAGCTGGAGGATGGCCGTACCCTCGCTGACTACAACATCCAGAAGGAGTCCACCCTCCACCTTGTCCTGCGTCTGCGTGGTGGCATGCAGATCTTCGTGAAGACCCTCACTGGAAAGACTATCACCCTTGAGGTTGAGAGCTCTGACACCATCGACAATGTCAAGGCCAAGATCCAAGACAAGGAGGGCATTCCCCCGGACCAGCAGCGCCTGATCTTTGCTGGCAAGCAGCTGGAGGATGGTCGCACCCTCGCCGACTACAACATCCAGAAGGAGTCCACCCTTCACCTGGTGCTCCGCCTCCGTGGTGGTCAGTAATTGCCCTCTCACTCGACCTGCTGCTGCTGCTGTACCCTGTGTCGTCCTGTGGGTGCGCCTTTGTTCAAGTGCTGTCTCGTTTGTGTGAAGTCGTACTGTGTCTGGTTTAATGGACCATCGAGTCCTGTGGTTTGGTGCAAACAGTTGCAGGTACAT

>TraesCS6A02G136600

MYLQLFAPNHRTRWSIKPDTVRLHTNETALEQRRTHRTTQGTAAAAGRVRGQLLTTTEAEHQVKGGLLLDVVVGEGATILQLLASKDQALLVRGNALLVLDLGLDIVDGVRALNLKGDSLSSEGLHEDLHATTQTQDKVEGGLLLDVVVRKGAAILQLLAGKDQALLVRGNALLVLDLGLDIVDGVRALNLQSDGLAGEGLHEDLHATTQTQDKVEGGLLLDVVVSEGSAILQLLASKDETLLVRGNALLVLDLGLDIVDGVRRLHLKGDGLARQCLHKDLHLQRRITDRMVSIKEADRQTHNDRTTNTNAWTSFYGSSIFFHLKILMTIGPCM*

**Supplementary Figure S9.** **The gDNA sequence of the gene corresponded to the sequences of four Y2H cDNA clones.** *TraesCS6A02G136600* corresponded to five cDNA clones (Supplemental Figure S7) identified from the Y2H library. The sequences of four cDNA clones (highlighted in grey) corresponded to the genome DNA sequence of *TraesCS6A01G136600*. However, due to a wrong orientation, this gene was annotated to encode a protein. The sequences of protein deduced from *TraesCS6A01G136600* have no identity to the deduced proteins from the four cDNAs.

**A**

>TraesCS6A01G112400.1

MQIFVKTLTGKTITLEVESSDTIDNVKAKIQDKEGIPPDQQRLIFAGKQLEDGRTLADYNIQKESTLHLVLRLRGG

MQIFVKTLTGKTITLEVESSDTIDNVKAKIQDKEGIPPDQQRLIFAGKQLEDGRTLADYNIQKESTLHLVLRLRGGQ*

>TraesCS6A01G119700.1

MQIFVKTLTGKTITLEVESSDTIDNVKAKIQDKEGIPPDQQRLIFAGKQLEDGRTLADYNIQKESTLHLVLRLRGG

MQIFVKTLTGKTITLEVESSDTIDNVKAKIQDKEGIPPDQQRLIFAGKQLEDGRTLADYNIQKESTLHLVLRLRGGQ*

>TraesCS6A01G136700.1

MQIFVKTLTGKTITLEVESSDTIDNVKAKIQDKEGIPPDQQRLIFAGKQLEDGRTLADYNIQKESTLHLVLRLRGG

MQIFVKTLTGKTITLEVESSDTIDNVKAKIQDKEGIPPDQQRLIFAGKQLEDGRTLADYNIQKESTLHLVLRLRGG

Q*

>TaUBV1-6A

xxxxxxxxxxxxxxxxxxxxxxxxxVKAKIQDKEGIPPDQQRLIFAGKQLEDGRTLADYNIQKESTLHLVLRLRGG

MQIFVKTLTGKTITLEVESSDTIDNVKAKIQDKEGIPPDQQRLIFAGKQLEDGRTLADYNIQKESTLHLVLRLRGGQ

>TraesCS7D01G443100.1

MQIFVKTLTGKTITLEVESSDTIDNVKAKIQDKEGIPPDEQRLIFAGKQLEDGRTLADYNIQKESTLHLVLRLRGG

MQIFVKTLTGKTITLEVESSDTIDNVKAKIQDKEGIPPDQQRLIFAGKQLEDGRTLADYNIQKESTLHLVLRLRGG

MQIFVKTLTGKTITLEVESSDTIDNVKAKIQDKEGIPPDQQRLIFAGKQLEDGRTLADYNIQKESTLHLVLRLRGGQ*

>TraesCS5B01G080200.1

MQIFVKTLTGKTITLEVESSDTIDNVKAKIQDKEGIPPDQQRLIFAGKQLEDGRTLADYNIQKESTLHLVLRLRGG

MQIFVKTLTGKTITLEVESSDTIDNVKAKIQDKEGIPPDQQRLIFAGKQLEDGRTLADYNIQKESTLHLVLRLRGGQ*

>TraesCS5B01G080300.1

MQIRLSPSMRSITISSSNGLLDLMKLKTAARHFSYRTVFHTVLILAFLLPFVFILTAVMTLEGFNKCSSLDCLGRRLGPRLLGRGEDGSMRLVRDLYRMLDEINSEEVPVDLKVAESFDDFIWDTKNNDYDLKSFALRLKATMETMDKELRSSRLSEQLNKHYAAIAIPKGLYCLSLRLTDVYSSNALARKQLPPPELVPRLSDNSYFHFVLASDNILAASVVVRSTVRSALKPERIVFHVITDKKTYPAMHSWFALNPLYPAIIEVKGVHQFEWLTKENGPVLEAIEIQHIARSRYHGNHLARTTADDSPRVFAAKLQAGSPTYTSVLNHIRIYLPELFPSLSKVVFLDDDVVVQHDLSSLWDIDLAGKVNGAVETCRGGDSWVMSKRFRNYFNFSHPLIATNFDPLECAWAYGMNIFDLAAWRKTTIKDKYHHWVKENLKSNFTLWRLGTLPPGLIAFKGHVHPIDPSWHLLGLGYQEKTDISSVRKAAVIHYNGQSKPWLDIGFKHLQPFWTKHVNYSNEFVRNCHIMEPQL*

**B**


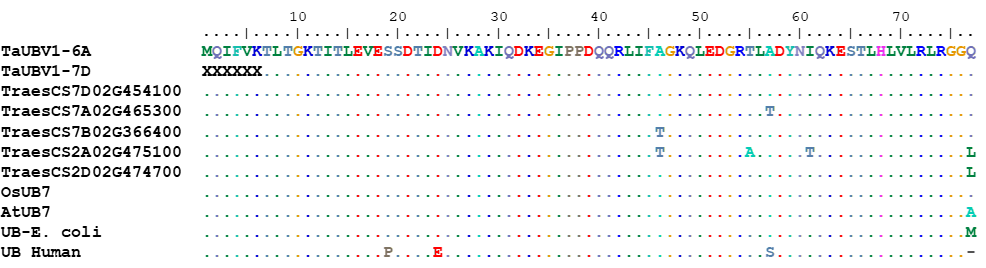


*

*

*

*

*

*

*

*

*

-

-

K6 K11 K27 K29 K33 K48 K63

**Supplementary Figure S10. Ubiquitin sequences deduced from Y2H cDNA clones. A**, Ubiquitin sequences deduced from Y2H cDNA clones. Two clones, LLY705 and LLY708, hit a genomic region where no gene was annotated but was 12 kb far away from *TraesCS7D02G443100* that is annotated as ubiquitin with three copies with 100% identity to our UB encoded by LLY705 and LLY708 sequences. The Y2H clone LY733 hit *TraesCS5B02G080200* on chromosome 5B, which encodes three copies of UB. **B**, Multiple sequence alignment of ubiquitin sequences. The conserved residues K6, K11, K27, K29, K33, K48, and K63 are indicated. Star * indicates stop codon.


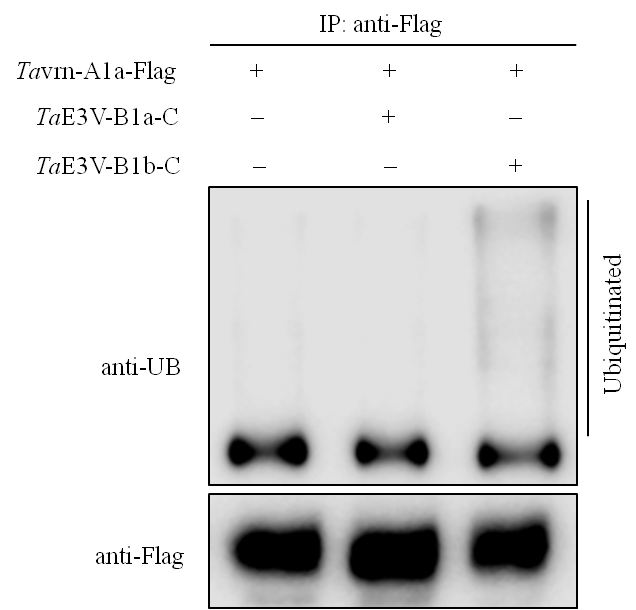


**Supplementary Figure S11. *In vivo* ubiquitination assay of *Ta*vrn-A1a mediated by *Ta*E3V-B1 in *N. benthamiana* leaves.** Total proteins were extracted and incubated with anti-GFP-conjugated magnetic agarose beads (MBL) for immunoprecipitation (IP). Proteins before (Input) and after IP were detected with anti-GFP and anti-Flag antibodies (Abclonal). *Ta*E3V-B1-C, the truncated protein including the C terminus (from residue 78 to the C-terminus ).


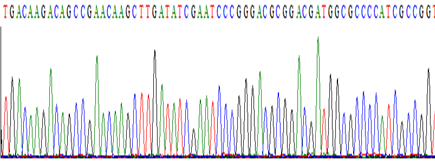

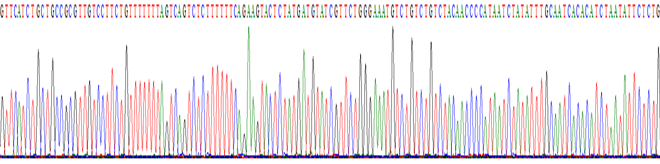

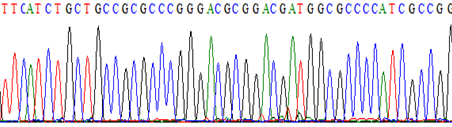


**Supplementary Figure S12. Editing events in the *TaE3V-A1* gene.** The wildtype sequence is shown in the upper figure. The PAM sequence is indicated with a red square. The 159 bp insertion in the *TaE3V-A1-ED1* allele is indicated using two arrows.


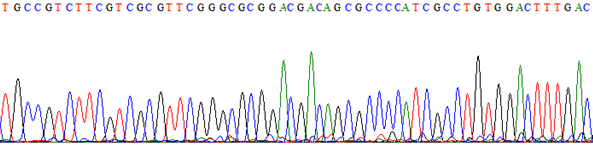


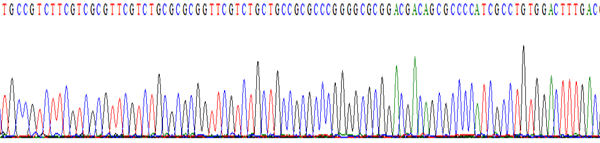


**Supplementary Figure S13. Editing events in the *TaE3V-B1* gene.** The wildtype sequence is shown in the lower figure. The PAM sequence is indicated with a red square. The 32 bp deletion in the *TaE3V-B1-ED1* allele is indicated using two arrows.


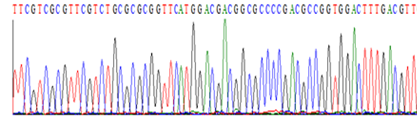


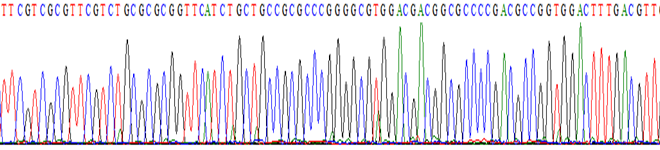


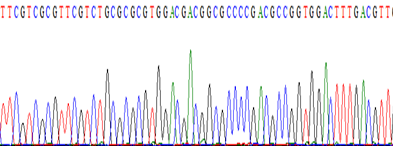


**Supplementary Figure S14. Editing events in the *TaE3V-D1* gene.** The wildtype sequence is shown in the middle. The PAM sequence is indicated with a red square. *TaE3V-D1-ED1* had a 21 bp deletion on the upper, and *TaE3V-D1-ED2* had a 26 bp deletion on the lower.

**Supplementary Table S1. Primers used in this study.**

| **Primer name** | **Primer sequence (5**′**-3**′**)** ^c^ | **Usage** | **Products** |
| --- | --- | --- | --- |
| *Ta*E3V-A1F1-RT | GACTTTGACGTTGACTTCCCA | cDNA | 139 bp |
| *Ta*E3V-A1R1-RT | AGCACTGGGCATCATCCTTT |  |  |
| *Ta*E3V-B1F1-RT | GACTTTGACGTTGACTTCCCG | cDNA | 185 bp |
| *Ta*E3V-B1R1-RT | ATGATTCTTAGAAGCTCTTTCTCG |  |  |
| *Ta*E3V-D1F1-RT | GACTTTGACGTTGACTTCCCG | cDNA | 139 bp |
| *Ta*E3V-D1F1-RT | AGCACTGGGCATCATCCTTC |  |  |
| E3A-S1F | CAGCGATGATGTTCGGGTCG | gDNA | 245 bp |
| E3A-S1R | TAGCAGCAGAATATCGATCGTT |  |  |
| E3B-S1F | CAACGATGATCTTTGGGTCC | gDNA | 274 bp |
| E3B-S1R | CGCTGTAGCACTAGCAAATAAAC |  |  |
| E3D-S1F | CAACGATGATCTTCGGGTCG | gDNA | 287 bp |
| E3D-S1R | ATGCGTAGTTATGTGTTCCCCT |  |  |
| TaVRN1-BD-F | CCGAATTCATGGGGCGGGGGAAGGT | Y2H | 735 bp |
| TaVRN1-BD-R | ACGGATCCTCACCCGTTGATGTGGCT | Y2H |  |
| TaE3V1-AD-F | CGGAATTCATGAAGTACTCTGAGGCTTT | Y2H |  |
| TaE3V1-AD-R1 | CGGGATCCTTATTGGCGTATCTCAATAA | Y2H | 369 bp |
| TaE3V1-AD-R2 | CGGGATCCTCAGTGCGGGGGCACT | Y2H | 249 bp |
| TaVRN1-nLUC-F | CGGGGGACGAGCTCGGTACCATGGGGCGGGGGAAGGT | LCI | 732 bp |
| TaVRN1-nLUC-R | ACGAGATCTGGTCGACCCCGTTGATGTGGCTCACC | LCI |  |
| TaE3V1-cLUC-F | ACGCGTCCCGGGGCGGTACCATGATCTTTGGGTCCGG | LCI |  |
| TaE3V1-cLUC-R1 | AGCTCTGCAGGTCGACTTATTGGCGTATCTCAATAA | LCI | 600 bp |
| TaE3V1-cLUC-R2 | AGCTCTGCAGGTCGACTCAGTGCGGGGGCACT | LCI | 480 bp |
| TaVRN1-nYFP-F | CGATAGTACTGTCGACATGGGGCGGGGGAAGGT | BiFC | 732 bp |
| TaVRN1-nYFP-R | TACCCTCGAGGTCGACCCCGTTGATGTGGCTCACC | BiFC |  |
| TaE3V1-cYFP-F | CGATAGTACTGTCGACATGATCTTTGGGTCCGG | BiFC |  |
| TaE3V1-cYFP-R1 | TACCCTCGAGGTCGACTTGGCGTATCTCAATAACC | BiFC | 597 bp |
| TaE3V1-cYFP-R2 | TACCCTCGAGGTCGACGTGCGGGGGCACTTGA | BiFC | 477 bp |
