# Supplementary material for: The E3 ligase TaE3V-B1 ubiquitinates proteins encoded by the vernalization gene TaVRN1 and regulates developmental processes in wheat
Source: Plant Physiol. 2024 Nov 18;197(1):kiae606. doi: 10.1093/plphys/kiae606 (PMC11663705; doi:10.1093/plphys/kiae606)
Supplement: kiae606_Supplementary_Data [file pp.00519.2024-s05.pdf › pp.00519.2024-s05.pdf]

Plant Physiology®

Regulation of developmental process by TaE3V-B1 mediated ubiquitination of TaVRN1 proteins in wheat

Tian Li, Ragupathi Nagarajan, Shujuan Liu, Juan Luzuriaga, Wenxuan Zhai, Shuanghe Cao, Haiyan Jia, Brett Carver, and Liuling Yan

Review Timeline:

Submission Date:	21-Sep-2023
Editorial Decision:	19-Nov-2023
Resubmission Received:	04-Apr-2024
Editorial Decision:	18-Apr-2024
Revision Received:	31-May-2024
Editorial Decision:	21-Jun-2024
Revision Received:	01-Oct-2024
Accepted:	15-Oct-2024

18-Nov-2023

Prof. Dr. Liuling Yan
Oklahoma State University
Plant and Soil Sciences
Stillwater, Oklahoma 74078

RE: Regulation of wheat maturity time by TaE3V-B1-mediated ubiquitination of TaVRN1 proteins

Dear Dr. Liuling Yan:

Thank you for giving us the opportunity to review your manuscript for publication in the Plant Physiology. The reviewers have found the manuscript of interest, but have raised a number of concerns that will require your attention. As these concerns are of some gravity and will entail substantial changes to the manuscript, you are likely to need additional time to address them. I must therefore reject the manuscript in its present form. Nonetheless, if you are able to address the points raised in review, I would welcome your resubmission of a manuscript that incorporates your responses.

In particular, you are encouraged to provide more direct evidence that TaE3V1 ubiquitinates TaVRN1, and to generate further results to support a physical interaction between the two proteins. Please note that any resubmission will be treated as a new entry to the system and is likely to go out for review.

If you do have any queries or concerns, you are welcome to contact me in the first instance.

Thank you again for allowing Plant Physiology to review your work. We look forward to hearing from you soon.

Sincerely,

Editor
Plant Physiology

-- Reviewer comments:
Reviewer #1 (Comments for the Author):

Thank you for your well and clearly written study. I find your methods adequate and agree with your conclusions.

Reviewer #2 (Comments for the Author):

In this manuscript, the authors identify a C3H2C3 RING-type E3 ligase TaE3V1 by yeast two hybrid screen with TaVRN1 as bait, which is associated with early maturity. The authors also generate many edited alleles of the three homoeologous TaE3V1, which can be used to accelerate maturity time during wheat breeding. This work reports a new protein interacting with TaVRN1 that may participate in regulating wheat maturity time. However, there are major issues that the authors need to address. Major issues:

1. The authors hypothesis that TaE3V-B1b encoded by the functional allele interacted with and ubiquitinated the TaVRN1 protein by adding the wheat ubiquitin TaUBV1. However, there is no evidence to show TaE3V1 could ubiquitinate TaVRN1. As ubiquitination is a common process for protein degradation, it would be better to show the ubiquitinated process of TaVRN1 by TaE3V1 is specific.
2. The authors emphasize the effect of TaE3V1 on the regulation of wheat maturity time, however, there are few results related to the wheat maturity time.
3. In lines 166-167, the partial colocalization of TaVRN1 and TaE3V1 should be supported by your experiment rather than citing the result from a previous study.
4. The evidence for the interaction between TaE3V1 and TaVRN1 in yeast is not strong, more interactions include interactions in vivo are recommended.
5. For association of allelic variants analysis, are there interesting natural variants in TaE3V-A1 and TaE3V-D1? Besides, the significant difference between TaE3V-B1a allele and TaE3V-B1b allele on heading date is weak in the RIL population, and that effect could be caused by other linked genes.
6. It is hard to compare effects of all the edited TaE3V1 alleles by looking at plant pictures in Figure 5, a summarized table or figure are recommended. From lines 300 - 313, are the two alleles of TaE3V-B1-ED1, B3-4-4, B3-4-1 have different effect on maturity and plant height? If yes, how to explain it?

Minor issues:
7. Line 73, Line 75 references were wrong cited.
8. Lines 106 - 113, it seems to me, that is not the introduction of the plant physiological maturity pathway. And in line 112, "a 168-bp insertion within Tavrn1 intron 1, where the repressor binding sites are also located" should be "a 168-bp insertion within its intron 1, where the MADS-box proteins binding sites are also located".
9. The result of Figure 2B is contradictory with the descriptions in lines 214 - 219, which TaE3Va is the one interacts with Tavrn1-a1a and Tavrn1-a1b.
10. Line 294, change "TaEV3-B" to "TaE3V-B". There are two names TaE3V1 and TaE3V in the context, please keep it uniform.
11. Line 305, delete "due to different combinations".

Reviewer #3 (Comments for the Author):

In wheat flowering, TaVRN1 protein play a central role as flowering promoter. In this study, the authors identified TaE3V1, a kind of E3 ligase that interacts with the TaVRN1 protein, is associated with early maturity. They focused on semi-winter cultivar Jagger with TaE3V-B1b and strong winter cultivar 2174 with TaE3V-B1b. They found that TaE3V-B1b functions as an E3 ligase but that TaE3V-B1a is unfunctional. Furthermore, they fund that TaVRN1 is a substrate for TaE3V1-mediated ubiquitination.

The objective of this study is clear, the experiments are wonderfully done, and the results are clear. It has long been recognized that the behavior of the VRN1 protein in the plant is important in wheat flower development, and this is an excellent study that provides a clue to this question. The only weakness of this study is the lack of data on the quantification of VRN1 protein. Of course, this is the next step in the research, but the importance of the variation in the amount of VRN1 protein should be mentioned in the text, e.g., in the Introduction and Discussion Since VRN1 protein is thought to directly activate the Florigen gene, the following paper also should be cited to discuss the importance of the amount of VRN1 protein.

Tanaka, C., T. Itoh, Y. Iwasaki, N. Mizuno, S. Nasuda and K. Murai (2018) Direct interaction between VRN1 protein and the promoter region of the wheat FT gene. Genes Genet. Syst. 93: 25-29.

Reviewer comments:

Reviewer #1 (Comments for the Author):

Thank you for your well and clearly written study. I find your methods adequate and agree with your conclusions.

Reviewer #2 (Comments for the Author):

In this manuscript, the authors identify a C3H2C3 RING-type E3 ligase TaE3V1 by yeast two hybrid screen with TaVRN1 as bait, which is associated with early maturity. The authors also generate many edited alleles of the three homoeologous TaE3V1, which can be used to accelerate maturity time during wheat breeding. This work reports a new protein interacting with TaVRN1 that may participate in regulating wheat maturity time. However, there are major issues that the authors need to address.

Major issues:

1. The authors hypothesis that TaE3V-B1b encoded by the functional allele interacted with and ubiquitinated the TaVRN1 protein by adding the wheat ubiquitin TaUBV1. However, there is no evidence to show TaE3V1 could ubiquitinate TaVRN1. As ubiquitination is a common process for protein degradation, it would be better to show the ubiquitinated process of TaVRN1 by TaE3V1 is specific.

Response: We really appreciate your comments. We performed an *in vitro* ubiquitination assay using recombinant MBP-TaE3V-B1 and His-TaVRN1 proteins. The assay showed that *Ta*E3V-B1b from 2174 could ubiquitinate His-Tavrn-A1a from Jagger in the presence of E1, E2, and UB, while *Ta*E3V-B1a from Jagger did not exhibit this activity (**Figure 3C**). The absence of any components, including E1, E2, UB, or *Ta*E3V-B1b, abolished the ubiquitination of the *Ta*vrn-A1a protein in the assay (**Figure 3C**). The capability of *Ta*E3V-B1b ubiquitinating the His-*Ta*vrn-A1b from 2174 was also confirmed (**Figure 3D**). These results provided direct evidence that *Ta*E3V-B1b mediated the ubiquitination of the *Ta*VRN1 protein.

2. The authors emphasize the effect of TaE3V1 on the regulation of wheat maturity time, however, there are few results related to the wheat maturity time.

Thank you for your comment on the phenotype. We agree that we did not have direct evidence for the specific trait regulated by the *TaE3V1* gene. We reorganized the results and made significant changes to this section.

We changed the title to ‘Regulation of developmental process by *Ta*E3V-B1 mediated ubiquitination of *TaVRN1* proteins in wheat.’ The developmental process included the heading date (flowering time) associated with natural alleles, as shown in Figure 4B, and the maturity stage associated with some editing alleles, as shown in Figure 6, in the *TaE3V1* gene.

3. In lines 166-167, the partial colocalization of TaVRN1 and TaE3V1 should be supported by your experiment rather than citing the result from a previous study.

1) **Response:** Thank you for your suggestion. We redid an experiment with *Ta*VRN1 and *Ta*E3V1 using the same research system. We fused the *Ta*E3V1 protein with a

green fluorescent protein (GFP) and the *Ta*VRN1 protein with a red fluorescent protein (RFP). *Ta*E3V1-GFP and *Ta*VRN1-RFP fusion proteins were co-expressed in plant cells, and their localizations overlapped at the membrane, suggesting that they may have interactions (**Figures B-G**).

4. The evidence for the interaction between TaE3V1 and TaVRN1 in yeast is not strong, more interactions include interactions in vivo are recommended.

Response: We appreciate your comments. We conducted two additional experiments. The new results confirmed the interaction between *Ta*E3V1 and *Ta*VRN1.

1) We experimented with the interaction between *Ta*E3V-B1 and *Ta*vrn-A1 through the firefly luciferase complementation imaging (LCI) assay in *N. benthamiana* leaves. The results showed that the full-length *Ta*E3V-B1b with LUC (*Ta*E3V1b-cLUC) and the truncated *Ta*E3V-B1b from residue 78 to the end of the C-terminus (*Ta*E3V-B1b-C) with LUC (*Ta*E3V1b-C-cLUC) interacted with *Ta*vrn-A1a in plant cells, but the full-length *Ta*E3V-B1a and its truncated form did not with *Ta*vrn-A1a in plant cells (**Figure 2B**). Furthermore, the truncated *Ta*E3V-B1b had much more robust interactions with *Ta*vrn-A1 than the full-length *Ta*E3V-B1b, probably because the N-terminal 77 a.a. containing the transmembrane domains hindered their interactions. In addition, the differential interactions of *Ta*E3V-B1a and *Ta*E3V-B1b proteins with the *Ta*vrn-A1a protein were validated in their interactions with the *Ta*vrn-A1b protein (**Supplemental Figure S6A**). These results proved that the *Ta*E3V1 proteins directly interacted with the *Ta*VRN1 proteins.
2) We further validated the direct interactions between *Ta*E3V1 and *Ta*VRN1 using the bimolecular fluorescence complementation (BiFC) assay. The results are provided in Figures 2, C, and D, and **Supplemental Figure S6B**.

5. For association of allelic variants analysis, are there interesting natural variants in TaE3V-A1 and TaE3V-D1? Besides, the significant difference between TaE3V-B1a allele and TaE3V-B1b allele on heading date is weak in the RIL population, and that effect could be caused by other linked genes.

We agree with this comment. Any gene that is located close to the *TaE3V-B1* gene could be the gene causing the phenotypic variation. Therefore, for the natural alleles, we used ‘associated’ but not ‘caused’ for the phenotypic effect of the *TaE3V-B1* gene.

We did not observe any difference in *the TaE3V-A1 or TaE3V-D1 gene between Jagger and 2174. We did not observe any QTL in the genomic regions covering the TaE3V-A1 and TaE3V-D1 genes. Therefore, we were not able to make any comments on the effects of the TaE3V-A1 and TaE3V-D1 genes*, if any, in the population we used in this study.

6. It is hard to compare effects of all the edited TaE3V1 alleles by looking at plant pictures in Figure 5, a summarized table or figure are recommended. From lines 300 - 313, are the two alleles of TaE3V-B1-ED1, B3-4-4, B3-4-1 have different effect on maturity and plant height? If yes, how to explain it?

Response:

Thank you for pointing out this issue. We found it challenging to make a summary for the effects of a single editing event on a single trait because there were different editing alleles of *TaE3V-A1*, *TaE3V-B1*, and *TaE3V-D1* and several visible traits changed in the edited plants. We claimed the developmental process in the study only because it is consistent with the phenotype we observed in the RIL population. We provided the significant differences in different populations in the text and the genotypes in the legend. Although we attempted to separate the different editing events and traits in seven T2 populations, much more extensive experiments would be required to conclude the phenotypic effects of each editing event. Further experiments on each population are beyond the scope of the current work. We appreciate your understanding. In the discussion section, we made the following comments:

"*This study obtained seven edited events in the three homoeologous TaE3V1 genes. The stacking and pyramiding of edited alleles in the same plant without any crosses is now possible. However, the dosage-dependent effect of TaE3V1 function on the development process was unclear, as we did not observe all genetic combinations of edited genes. We did, however, observe differences in phenotypes between plants homozygous for one or two edited genes, suggesting a complex regulatory hierarchy among homoeologs and potential compensatory mechanisms. A thorough phenotypic investigation of all possible genotypes (wild type, three single mutants, three double mutants, and one triple mutant) with Cas9 transgene removed will be intriguing in future studies*."

Minor issues:

7. Line 73, Line 75 references were wrong cited.

Response: We corrected the errors.

8. Lines 106 - 113, it seems to me, that is not the introduction of the plant physiological maturity pathway. And in line 112, "a 168-bp insertion within Tavrn1 intron 1, where the repressor binding sites are also located" should be "a 168-bp insertion within its intron 1, where the MADS-box proteins binding sites are also located".

Response: Thanks for your comments. We revised the sentence per your suggestion in the revised manuscript.

9. The result of Figure 2B is contradictory with the descriptions in lines 214 - 219, which TaE3Va is the one interacts with Tavrn1-a1a and Tavrn1-a1b.

Response: Apologies for our carelessness. The TaE3V-B1b can interact with TaVRN1, while the mutant TaE3V-B1a lacking full C terminus can not. We corrected the legend for this Figure.

10. Line 294, change "TaEV3-B" to "TaE3V-B". There are two names TaE3V1 and TaE3V in the context, please keep it uniform.

Response: Sorry for our negligence. We corrected "TaEV3-B" to "TaE3V-B" in the

revised manuscript.

11. Line 305, delete "due to different combinations".

Response: We deleted "due to different combinations".

Reviewer #3 (Comments for the Author):

In wheat flowering, TaVRN1 protein play a central role as flowering promoter. In this study, the authors identified TaE3V1, a kind of E3 ligase that interacts with the TaVRN1 protein, is associated with early maturity. They focused on semi-winter cultivar Jagger with TaE3V-B1b and strong winter cultivar 2174 with TaE3V-B1b. They found that TaE3V-B1b functions as an E3 ligase but that TaE3V-B1a is unfunctional. Furthermore, they fund that TaVRN1 is a substrate for TaE3V1-mediated ubiquitination.

The objective of this study is clear, the experiments are wonderfully done, and the results are clear. It has long been recognized that the behavior of the VRN1 protein in the plant is important in wheat flower development, and this is an excellent study that provides a clue to this question. The only weakness of this study is the lack of data on the quantification of VRN1 protein. Of course, this is the next step in the research, but the importance of the variation in the amount of VRN1 protein should be mentioned in the text, e.g., in the Introduction and Discussion Since VRN1 protein is thought to directly activate the Florigen gene, the following paper also should be cited to discuss the importance of the amount of VRN1 protein.

Tanaka, C., T. Itoh, Y. Iwasaki, N. Mizuno, S. Nasuda and K. Murai (2018) Direct interaction between VRN1 protein and the promoter region of the wheat FT gene. Genes Genet. Syst. 93: 25-29.

Response: We added the critical information in the revised introduction and added the research article in the revised references.

18-Apr-2024

Dr. Liuling Yan
Oklahoma State University
Plant and Soil Sciences
Stillwater, Oklahoma 74078

RE: Regulation of developmental process by TaE3V-B1 mediated ubiquitination of TaVRN1 proteins in wheat

Dear Dr. Yan:

We have received reviews of your manuscript, which you may view at the end of this letter. On the basis of the advice received and my own evaluation, I believe that, pending revision, this article may ultimately be acceptable for publication in Plant Physiology.

In particular, please make sure to include relevant data and controls to support all your conclusions, as specified by Reviewer #2. If new data (e.g. NILs vs. RILs) cannot be provided, be sure to revise your conclusions accordingly.

When you are ready to submit your revision, click the below link and find the red arrow under your Author Tasks heading for this revision submission.

If you want to visit this version of the manuscript, you may click the link below and then click on the folder that reads "Post Decision Manuscripts".

Link Not Available

When uploading the revised version of this article, please be sure to include as complete an answer as possible to the referees' criticisms during the first round of reviews in the "response to reviewers" section.

Plant Physiology considers a revision for up to 60 days after a manuscript has been provisionally accepted; limited extension of this timeframe is at the discretion of the Monitoring Editor. A reminder notice will be sent to you 2 weeks before the deadline, at which time you may choose to contact the editorial office to request an extension; if so, please provide a justification for your request. After the expiration of this deadline, the article would need to be submitted as a resubmission and would be handled as such.

Thank you very much for giving us an opportunity to review this work. I look forward to receiving the next version.

Sincerely,

Editor
Plant Physiology

Want to add this revision deadline to your calendar? Click below!

--- Reviewer comments:
Reviewer #2 (Comments for the Author):

The authors have provided elaborate explorations and additional information regarding my main recommendation. But I still have several concerns that need to be addressed and explained.
1. There are some unclear questions of "Association of allelic variants for TaE3V-B1 with heading date" need to address. Such as, is TaE3V-B1 located in a flowering time QTL interval? What materials were used for allele effect evaluation of TaE3V-B1 in this paper? Are you using the near isogenic lines (NILs) of TaE3V-B1a and TaE3V-B1b to evaluate the effect? If not, it would be

better to use NILs to exclude the effect of the genetic background rather than RILs. Otherwise, the evidence is not enough for the conclusion in Line 280-282.
2. It would be better to add a negative control for the LCI and BIFC assay in Figure 2. Also, there is no result showing the empty vector was used as a negative control in Supplemental Figure S4.
3. The developmental stage of samples used in Figure 5I is better to be indicated. A summarized figure shows the edited alleles of TaE3V in each line in Figure 5I and Figure 6 is better to be include in the figure.
4. The author said "TaE3V-A1 transcript levels were more than ten-fold higher than those of TaE3V-B1 and TaE3V D1", however, in Figure 5I, the protein level of TaE3V in plant 1 and 2 which have TaE3V-A1 edited alleles were higher than in plant 4 and 5 which have TaE3V-B1 allele. It would be better if the author could explain it or repeat this experiment.
5. The flowering time and maturity, number of spikes per plant, and plant height data of each line in Figure 6 is better to be indicated in the Figure.
There are still some flaws need to be minor revised:
6. Line 204, add a word "interact" in the front of "with Tavrn-A1a in plant cells".
7. Line 279, $P<0.03$ is a significant level please double check it. I assume the author wants to say $P>0.03$ and $P>0.18$. Typically, most people use a p-value less than significant level 0.05 or 0.01 indicates statistically significant.
8. Line 291-293, the sgRNA sequence listed in the method is same to TaE3V-A1 and TaE3V-D1 rather than TaE3V-B1. Please double check it.
9. The five lines in Figure 5I were indicated as "TaE3V-B1 edited plant", which is not consistent with the description in the text "The A1 line carried edited alleles at TaE3V-A1 and TaE3V-D1", please correct it.
10. The PAM sequence was wrongly indicated in Supplemental Figure S11 and S12.
11. Line 769 in supplemental, "deletion" to "insertion".

Reviewer #3 (Comments for the Author):

The manuscript has been revised according to the reviewer's comments.

Reviewer #2 (Comments for the Author):

The authors have provided elaborate explorations and additional information regarding my main recommendation. But I still have several concerns that need to be addressed and explained.

The outstanding review report was very helpful in revising the manuscript.

1. There are some unclear questions of "Association of allelic variants for TaE3V-B1 with heading date" need to address. Such as, is TaE3V-B1 located in a flowering time QTL interval? What materials were used for allele effect evaluation of TaE3V-B1 in this paper? Are you using the near isogenic lines (NILs) of TaE3V-B1a and TaE3V-B1b to evaluate the effect? If not, it would be better to use NILs to exclude the effect of the genetic background rather than RILs. Otherwise, the evidence is not enough for the conclusion in Line 280-282.

Response: The population used to analyze the phenotypic effect of *TaE3V-B1* on the developmental process is a recombinant inbred line (RIL) population (lines 27—272). The effect was analyzed using a single marker for the allelic variation in *TaE3V-B1* rather than a QTL approach. We did not find a QTL in the *TaE3V-B1* region because the markers in this region are insufficient for mapping. However, the effect from a single marker is commonly applied in molecular breeding. We agree that such a phenotypic effect may not be directly from the analyzed gene but from other genes that are in the same region as *TaE3V-B1*. Therefore, we revised the conclusion in Lines 280-282 as follows.

These results indicated the development process under the condition without vernalization was associated with a genetic factor at the *TaE3V-B1* locus, suggesting that plants carrying the nonfunctional *TaE3V-B1a* allele or an unknown gene at this locus promoted the development process. If *TaE3V-B1b* was the causal allele, it encoded a repressor of the development process in wheat, possibly by degrading *Ta*VRN1.

2. It would be better to add a negative control for the LCI and BIFC assay in Figure 2. Also, there is no result showing the empty vector was used as a negative control in Supplemental Figure S4.

Response: Thanks for your comments. We performed these experiments with negative controls, but the controls could not be placed in the same leaves as shown in Figure 2. We added multiple negative controls in Supplemental Figure S6B for the interaction of *Ta*E3V-B1 and *Ta*vrn-A1b in a LCI assay presented in Fig. 2B. We also added multiple negative controls in Supplemental Figure 7B for the interaction of *Ta*E3V-B1 and *Ta*vrn-A1b in a BiFC assay presented in Fig. 2C.

We did not perform an empty vector as a control for the localization of *Ta*E3V1 in *N. benthamiana* leaves presented in Supplementary Figure S4, because we did not know that an empty vector is a requirement for the localization of a protein. We did not include this control in our several previous publications. It will take a week to add this control in a new experiment. We are sorry about it.

3. The developmental stage of samples used in Figure 5I is better to be indicated. A summarized figure shows the edited alleles of TaE3V in each line in Figure 5I and Figure 6 is better to be include in the figure.

Response: The proteins were extracted from the leaves of plants 2 weeks after planting. We added this information to the legend of the Figure and the Method in the revision.

We added the genotypes of the edited lines presented in Figure 5I and Figure 6.

4. The author said "TaE3V-A1 transcript levels were more than ten-fold higher than those of TaE3V-B1 and TaE3V D1", however, in Figure 5I, the protein level of TaE3V in plant 1 and 2 which have TaE3V-A1 edited alleles were higher than in plant 4 and 5 which have TaE3V-B1 allele. It would be better if the author could explain it or repeat this experiment.

Response: We understand your concerns on this point. It is not uncommon for the protein levels to show inconsistency with the transcriptional levels, possibly due to post-transcriptional and translational regulation, such as protein modifications and degradations. We added a sentence to describe and explain this inconsistency in the new version.

5. The flowering time and maturity, number of spikes per plant, and plant height data of each line in Figure 6 is better to be indicated in the Figure.

Response: Thanks for the suggestion. We added the genotypes and phenotypes of the edited lines in Figure 6.

There are still some flaws need to be minor revised:

6. Line 204, add a word "interact" in the front of "with Tavrn-A1a in plant cells".

Response: We changed as suggested.

7. Line 279, $P<0.03$ is a significant level please double check it. I assume the author wants to say $P>0.03$ and $P>0.18$. Typically, most people use a p-value less than significant level 0.05 or 0.01 indicates statistically significant.

Response: We agree that a p-value less than 0.05 or 0.01 indicates statistical significance. We are very sorry for a mistake in our writing here. The correct results should be that the population was vernalized for three weeks ($P = 0.07$) or six weeks ($P = 0.18$) (Figure 4B). In the previous version, we mistakenly wrote $P>0.03$ for $P = 0.07$ and $P>0.18$ for $P = 0.18$.

8. Line 291-293, the sgRNA sequence listed in the method is same to TaE3V-A1 and TaE3V-D1 rather than TaE3V-B1. Please double check it.

Response: We apologize for our carelessness. The sgRNA sequence was designed based on the sequences of *TaE3V-A1* and *TaE3V-D1* rather than *TaE3V-B1*. We have corrected the mistake.

Although TaE3V-B1 had one mismatch (T-G), the sgRNA still recognized and edited the sequence.

9. The five lines in Figure 5I were indicated as "TaE3V-B1 edited plant", which is not consistent with the description in the text "The A1 line carried edited alleles at TaE3V-A1 and TaE3V-D1", please correct it.

Response: It was a good finding. We corrected this mistake. Thanks.

10. The PAM sequence was wrongly indicated in Supplemental Figure S11 and S12.

Response: We placed the red square for PAM on the right site in the two Figures.

11. Line 769 in supplemental, "deletion" to "insertion".

Response: We really appreciate that you found this problem. We have corrected it.

21-Jun-2024

Dr. Liuling Yan
Oklahoma State University
Plant and Soil Sciences
Stillwater, 37 74078

RE: Regulation of developmental process by TaE3V-B1 mediated ubiquitination of TaVRN1 proteins in wheat

Dear Dr. Yan:

We have received reviews of your manuscript, which you may view at the end of this letter. On the basis of the advice received and my own evaluation, I believe that, pending revision, this article may ultimately be acceptable for publication in Plant Physiology.

In your revision, please pay particular attention to providing any caveats to your experiments/conclusions, and to provide possible explanations/hypotheses to explain inconsistent or lacking results needed for your model. Also provide quantitation of the various protein interaction assays where possible.

When you are ready to submit your revision, click the below link and find the red arrow under your Author Tasks heading for this revision submission.

If you want to visit this version of the manuscript, you may click the link below and then click on the folder that reads "Post Decision Manuscripts".

Link Not Available

When uploading the revised version of this article, please be sure to include as complete an answer as possible to the referees' criticisms during the first round of reviews in the "response to reviewers" section.

Plant Physiology considers a revision for up to 60 days after a manuscript has been provisionally accepted; limited extension of this timeframe is at the discretion of the Monitoring Editor. A reminder notice will be sent to you 2 weeks before the deadline, at which time you may choose to contact the editorial office to request an extension; if so, please provide a justification for your request. After the expiration of this deadline, the article would need to be submitted as a resubmission and would be handled as such.

Thank you very much for giving us an opportunity to review this work. I look forward to receiving the next version.

Sincerely,

Editor
Plant Physiology

Want to add this revision deadline to your calendar? Click below!

-- Reviewer comments:
Reviewer #2 (Comments for the Author):

Most of my previous questions and comments were clarified in the current manuscript. I have no more major questions but with few flaws.

1. The information of the population used to analyze the phenotypic effect of TaE3V-B1 should be included in the Methods.
2. It's fine without the negative control in the localization of TaE3V1. Delete the sentence "the empty vector was used as a

negative control." from line 318-319 in supplemental document.
3. Change the name of "TaE3V-D1" in Fig5H to "TaE3V-B1"

Reviewer #4 (Comments for the Author):

I have carefully read this revised manuscript and the response to the reviewer. I found this topic is interesting, but the data didn't fully support the conclusion. There are some suggestions that should be considered.
1.Fig1B-G. All bars were lost. And the intensity of overlapped signal should be calculated.
2.Fig 2B. All expressed proteins should be detected.
3.Fig 3C. The BiFC assay is not convincing. The interaction between VRN1 and E3V1b doesn't seem to be in the plasma-membrane. Further assays should be added. In addition, other experiments such as co-IP should be included.
4.The authors found that VRN1 was ubiquitinated by E3V1a. However, it remains unclear whether E3V1a meditates VRN1 ubiquitination in plants.
5.It should be noted that the flowering time of 2174 is much later than Jagger. However, the protein level of E3V1 in 2174 looks the same as in Jagger after vernalization. Why?
6.The authors need to detect the protein level of VRN1 in 2174 and Jagger before and after vernalization.

Reviewer #2 (Comments for the Author):

Most of my previous questions and comments were clarified in the current manuscript. I have no more major questions but with few flaws.

1. The information of the population used to analyze the phenotypic effect of TaE3V-B1 should be included in the Methods.

Response: The following information about the population used to analyze the phenotypic effect of TaE3V-B1 was added to the revision.

'Jagger' and '2174', two winter wheat cultivars, were crossed to generate a population of 96 F6:7 recombinant inbred lines (RILs) by single-seed descent from the F2 generation (Chen et al., 2009). This RIL population was tested for the developmental process with and without vernalization (Li et al., 2013).

2. It's fine without the negative control in the localization of TaE3V1. Delete the sentence "the empty vector was used as a negative control." from lines 318-319 in supplemental document.

Response: "the empty vector was used as a negative control" was deleted. Thanks.

3. Change the name of "TaE3V-D1" in Fig5H to "TaE3V-B1"

Response: It was corrected. Thanks.

Reviewer #4 (Comments for the Author):

I have carefully read this revised manuscript and the response to the reviewer. I found this topic is interesting, but the data didn't fully support the conclusion. There are some suggestions that should be considered.

1. Fig1B-G. All bars were lost. And the intensity of overlapped signal should be calculated.

Bars were added to Figures 1B-G.

Figures D and G were used to show the colocalization of *Ta*E3V1 and *Ta*vrn-A1a on the membrane but not the intensity of overlapped signals. We used the conventional method to show the colocalization of two proteins. We appreciate your understanding that calculating the overlapped signals with uneven distributions on membranes is a challenge.

2. Fig 2B. All expressed proteins should be detected.

Response: As requested, we used an anti-LUC antibody to show that all the nLUC and cLUC fusion proteins were expressed in the leaves. The result was provided in Supplementary Figure S6A. Therefore, the interaction of *Ta*E3V-B1 and *Ta*vrn-A1a in a luciferase complementation imaging (LCI) assay was further confirmed.

3. Fig 3C. The BiFC assay is not convincing. The interaction between VRN1 and E3V1b doesn't seem to be in the plasma membrane. Further assays should be added. In addition, other experiments, such as co-IP, should be included.

Response: We thought you had concerns about the BiFC assay presented in Fig. 2C

instead of Fig. 3C. Although *Ta*E3V-B1 was mainly localized in the plasma membrane, the *Ta*vrn-A1a was localized partly on the plasma membrane but mainly on the nucleus. The interaction of *Ta*E3V1b with *Ta*vrn-A1a was detectable. We performed co-IP assays to confirm their interaction in vivo. The result is provided in Figure 2C.

4. The authors found that VRN1 was ubiquitinated by E3V1a. However, it remains unclear whether E3V1a meditates VRN1 ubiquitination in plants.

Response: Thank you for your comments. We agree with your comment the result that TaE3V1a ubiquitinated the TaVRN1 protein should be validated in wheat plants. Since *Ta*VRN1 antibodies are needed for experiments, but no commercial antibodies for *Ta*VRN1 are available, we performed the ubiquitination assay in *N. benthamiana* leaves by co-expression of both *Ta*VRN1-Flag and *Ta*E3V1b. The result showed that *Ta*VRN1-Flag could be ubiquitinated by *Ta*E3V-B1b, not *Ta*E3V-B1a (as shown in Supplementary Figure S11. Therefore, we believe that E3V1a meditates *Ta*VRN1 ubiquitination in plants.

5. It should be noted that the flowering time of 2174 is much later than Jagger. However, the protein level of E3V1 in 2174 looks the same as in Jagger after vernalization. Why?

Response: The flowering time of 2174 is much later than that of Jagger when the two cultivars were grown under the condition without vernalization. The differential flowering time was mainly controlled by *Ta*vrn-A1 (Li et al., 2013) and partly regulated by *Ta*E3V-A1 (Fig. 4B, this study). After 6 weeks of vernalization, the protein level of *Ta*E3V1 in the two cultivars was increased (Fig. 3E-H, this study), which may cause all *Ta*VRN1 to degrade. *Ta*E3V1 was up-regulated by vernalization in wheat. The protein levels of *Ta*E3V1 in 2174 and Jagger after vernalization look similar, but these *Ta*E3V1 proteins included proteins encoded by three homoeologous *Ta*E3V1 genes. 2174 has functional *Ta*E3V-B1b, but Jagger has nonfunctional *Ta*E3V-B1a. Since these *Ta*E3V-B1 proteins could not be distinguished from the homoeologous *Ta*E3V1 proteins, it was impossible to make an association between the flowering time and the *Ta*E3V1 proteins including *Ta*E3V-A1, *Ta*E3V-B1, and *Ta*E3V-D1.

6.The authors need to detect the protein level of VRN1 in 2174 and Jagger before and after vernalization.

Response: Thank you for the excellent suggestion. We agree with the comment that the protein level of *Ta*VRN1 in 2174 and Jagger before and after vernalization should be investigated. The experiment should be performed using an anti-VRN1 antibody. However, no commercial *Ta*VRN1 is available. We cannot synthesize the *Ta*VRN1 antibody within several months. To compensate for this deficiency, we performed the ubiquitination assay in *N. benthamiana* leaves by co-expression of both *Ta*VRN1-Flag and *Ta*E3V1b. The result showed that *Ta*VRN1-Flag could be ubiquitinated by *Ta*E3V-B1b in plants (Supplementary Figure S11). I appreciate your understanding of the current situation.

15-Oct-2024
Dr. Liuling Yan
Oklahoma State University
Plant and Soil Sciences
Stillwater, 37 74078

MSID: PP2024-RA-00519DR2
MS TITLE: Regulation of developmental process by TaE3V-B1 mediated ubiquitination of TaVRN1 proteins in wheat

Dear Dr. Liuling Yan:

I am pleased to inform you that your revised manuscript "Regulation of developmental process by TaE3V-B1 mediated ubiquitination of TaVRN1 proteins in wheat" has been accepted for publication in Plant Physiology. Your manuscript will be checked for consistency with journal formatting and image standards by one of our Science Editors, who will contact you shortly. They may have a number of queries and suggestions for you. Please keep in mind that they work to increase the online visibility of your article and its attraction for readers from the widest of backgrounds. You will have the opportunity to review any revisions, and I hope you will work constructively with them to get the most out of your research. You will also have the opportunity to review all changes when you receive the author proofs for your article.

Once validated for publication, your article will be sent to a Science Editor for a final review of your manuscript and source files. After your final files are uploaded, your article will appear in the next available issue and the online Preview version of your article will be posted after your signed Copyright and Checklist forms and final source files are received.

Please be sure to include the following in your manuscript file:
Harvard style citations
List of all author contributions and funding information at the beginning of manuscript after the titles and author list.
List all supplemental materials associated with the article, with titles, at the end of the ms, before the references and acknowledgments section.
Complete Figure Legends for all figures appearing in the article listed before the Literature Cited.

LICENSE INFORMATION Shortly after your paper is sent to production, you will receive an email from our publisher's author support team, **SciPris**, with information on article processing charges, as well as the types of Open Access license you would be able to purchase. The accepted version of your manuscript will not appear on our Advance Articles page until the license is selected and signed, so please look out for an email from SciPris. For more information see the Article Publication Fees section of our author guidelines: https://academic.oup.com/plcell/pages/General-Instructions

Finally, we encourage your submission of artwork for the journal cover. If there is an image or illustration related to your paper that you would like to have considered, please email it in .pdf or .tif format to clowe@aspb.org. An image title, brief, 2-3 sentence description of the image, and the name of the person credited for the image are also encouraged. If your image is selected to appear on the online cover of the journal, you will be contacted with the scheduled issue date and further information.

Thank you and congratulations on your Plant Physiology paper!

Sincerely,
Jianhua Zhang
Associate Editor, Plant Physiology

=====
FIRST AUTHOR PROFILES
=====

We recognize the hard work that goes into being First Author of a Plant Physiology paper by publishing a "First Author Feature" for each First Author alongside your paper.

If the first author(s) would like to submit information for a feature on Facebook and Twitter, fill out the form by clicking on the link below:

Plant Physiology First Author Feature Form: https://forms.office.com/r/ZBCGZfhm8F
If you cannot access the form through the link above, please contact Rachel at rbelsky@aspb.org.

To upload an image of yourself to accompany your profile, please put your photo in the correct journal folder at the link below. You must label your image: Author First Name Last Name Article Title.

First Author Profile Image Dropbox: https://www.dropbox.com/scl/fo/xz029p7qjr2jml2rp2kmq/h?dl=0&rlkey=6fkxcaqmxx35ap152b2osy1fz

=====
IMPORTANT REMINDER: PEER REVIEW REPORTS
=====

If you opted to publish a peer review report along with your article during the original submission process, it will be prepared by the editorial staff and publicly posted with your manuscript, inside the zip file that contains any other supplemental material. As a reminder, the peer review report is a public record of all comments from editors and reviewers, as well as your prior responses, as you received them in the decision letters for each draft of your manuscript. If you agreed to publish this report and have changed your mind, or are not sure if you selected this option, please contact the editorial office as soon as possible before signing the license agreement from our publisher.

-- Reviewer comments:
